# Analyzing Structure–Activity Variations for Mn–Carbonyl Complexes in the Reduction of CO_2_ to CO

**DOI:** 10.1021/acs.inorgchem.2c03391

**Published:** 2022-12-21

**Authors:** Jacob Florian, Jacqueline M. Cole

**Affiliations:** † Cavendish Laboratory, University of Cambridge, J.J. Thomson Avenue, Cambridge CB3 0HE, U.K.; ‡ ISIS Neutron and Muon Source, STFC Rutherford Appleton Laboratory, Harwell Campus for Science and Innovation, Didcot OX11 0QX, U.K.

## Abstract

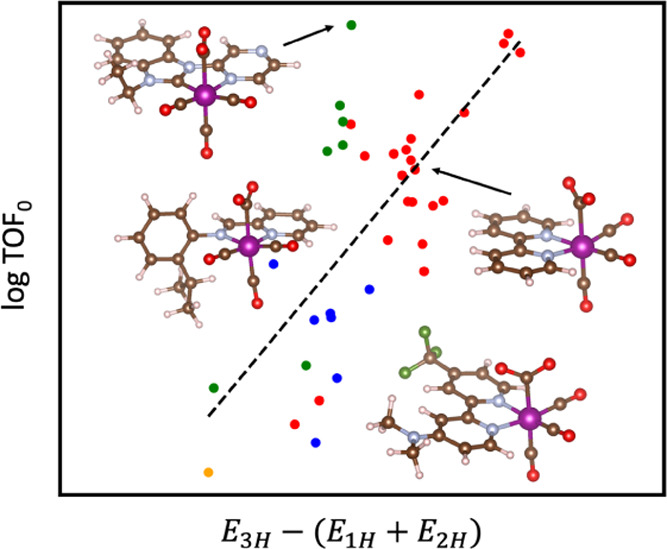

Contemporary electrocatalysts for the reduction of CO_2_ often suffer from low stability, activity, and selectivity, or a combination thereof. Mn–carbonyl complexes represent a promising class of molecular electrocatalysts for the reduction of CO_2_ to CO as they are able to promote this reaction at relatively mild overpotentials, whereby rare-earth metals are not required. The electronic and geometric structure of the reaction center of these molecular electrocatalysts is precisely known and can be tuned via ligand modifications. However, ligand characteristics that are required to achieve high catalytic turnover at minimal overpotential remain unclear. We consider 55 Mn–carbonyl complexes, which have previously been synthesized and characterized experimentally. Four intermediates were identified that are common across all catalytic mechanisms proposed for Mn–carbonyl complexes, and their structures were used to calculate descriptors for each of the 55 Mn–carbonyl complexes. These electronic-structure-based descriptors encompass the binding energies, the highest occupied and lowest unoccupied molecular orbitals, and partial charges. Trends in turnover frequency and overpotential with these descriptors were analyzed to afford meaningful physical insights into what ligand characteristics lead to good catalytic performance, and how this is affected by the reaction conditions. These insights can be expected to significantly contribute to the rational design of more active Mn–carbonyl electrocatalysts.

## Abstract 2

We derive physical models for the activity of Mn−Carbonyl catalysts by comparing their electronic descriptors to experimental kinetic data. One such model shows a positive correlation between the TOF of a catalyst at zero overpotential and the energy of HOMO orbitals of three species in the catalytic cycle. Each point represents a different catalyst, with colors representing different ligand types. Some points are labeled with their respective catalyst.

## Introduction

The electrochemical reduction of carbon dioxide (CO_2_) is a key reaction that can help significantly reduce global CO_2_ emissions and simultaneously provide a pathway to renewable fuels and value-added commodity chemicals using CO_2_ as a C1 synthon. Efficient methods for electrochemically converting CO_2_ into chemicals have not yet fully matured, and the development of electrocatalysts is at the forefront of this field.

Electrocatalysts for the reduction of CO_2_ come in many forms, including multifaceted electrodes, supported nanoparticles,^1^ as well as single-atom^2^ and molecular catalysts.^3^ Homogeneous molecular electrocatalysts provide a tunable platform where the ligand structure can be modified to achieve desired properties such as catalyst activity and selectivity. Unlike heterogeneous catalysts, where catalyst–support interactions are difficult to quantify and several different facets and adsorbates are usually present simultaneously, molecular electrocatalysts provide a controlled platform for mechanistic understanding and systematic improvements. When dealing with molecular electrocatalysts, the exact reaction site is often known and can therefore be modeled more accurately than for heterogeneous systems.

Molecular electrocatalysts for the reduction of CO_2_ are typically characterized by a single transition-metal center that acts as the active site to bind CO_2_ and other reactants. The metal center is usually coordinated by a ligand scaffold that is noninnocent, i.e., the ligand(s) actively participate in the redox process. Such noninnocent ligands can enhance the catalytic activity by acting as proton relays, electron reservoirs, or catalytic sites.^4^ Electrocatalysts for the reduction of CO_2_ are often adapted from other electrocatalysts (e.g., electrocatalysts for the reduction of H_2_ or for hydrogenation reactions), photocatalysts, or biological enzymes.^3^

Understanding the mechanisms that underpin electrocatalytically promoted reactions, especially the precise nature of the rate-limiting and other elementary steps, can be extremely beneficial and often necessary to improve the catalytic efficacy of the electrocatalyst. However, mechanistic studies are usually costly and time-consuming, generally requiring a combination of theory and experiment, controlled synthesis, and *in situ* materials characterization methods. The mechanism of a given electrochemically catalyzed reaction will often change if the composition of the electrocatalyst or the reaction conditions are changed, thus making it difficult to custom-tailor electrocatalysts based on mechanism alone. In contrast, high-throughput screening and black-box machine-learning (ML) models have become popular in this field because they enable a relatively cheap evaluation of a wide variety of different catalysts without requiring detailed mechanistic understanding. However, a problem with fast computational screening methods is that they do not usually provide information about why certain electrocatalysts perform well and thus render a systematic interpretation difficult.

By focusing on a subset of molecular electrocatalysts that share a similar structure and a common mechanism, insights can be gained into how changing different aspects of the catalyst will affect its performance. In this study, 55 Mn-centered molecular catalysts, which differ with respect to their ligand scaffold, were investigated using density functional theory (DFT) calculations to gain fundamental insights into what physicochemical factors influence catalytic activity and selectivity. The Mn–carbonyl set of molecular electrocatalysts represents a broad dataset to explore the effects of a variety of ligands while generally following a common mechanism. Mn–carbonyl catalysts consist of an octahedrally coordinated manganese center, a noninnocent ligand, and carbonyl ligands. Using molecular descriptors that are derived from individual mechanistic steps, as opposed to atomic or geometric descriptors, and correlating these to experimentally determined figures of merit to evaluate the molecular electrocatalysts, it is possible to gain insights into what features make a “good” electrocatalyst and why. These features are turned into simple and interpretable models that can be used by researchers to push the status quo of electrocatalyst development.

### Mechanistic Overview of CO_2_ Reduction by Mn–Carbonyl Complexes

**Figure 1:**
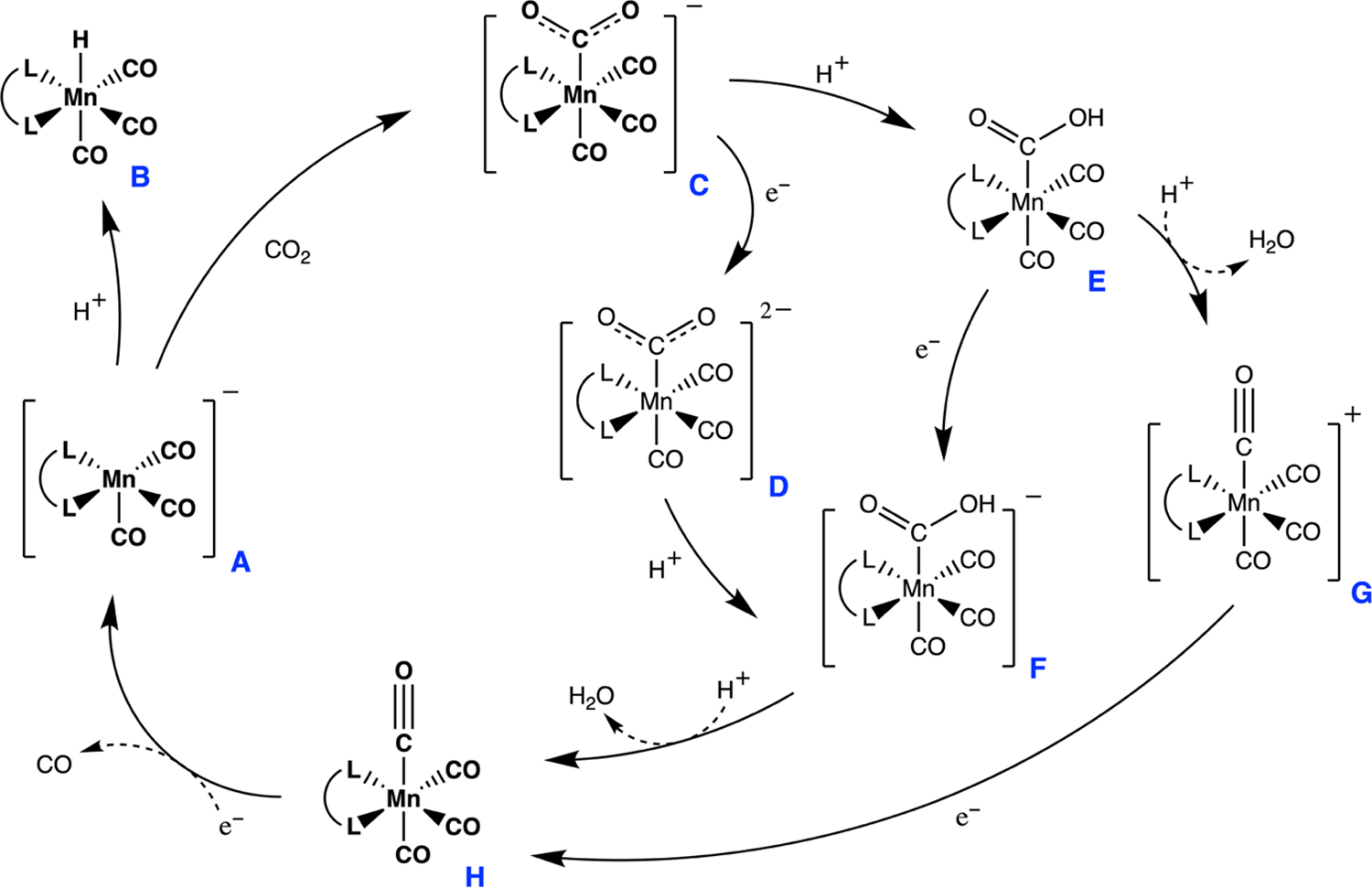
Electrocatalytic pathways for the reduction of CO_2_ with Mn–carbonyl complexes.

The current mechanistic understanding of how Mn–carbonyl complexes catalyze CO_2_ reduction will inform feature selection and is helpful for model interpretation. Mn–carbonyl complexes are synthesized as stable precatalysts, which must be reduced (i.e., gain electrons) to become coordinatively unsaturated and active for the binding of CO_2_. Mn–carbonyl precatalysts are often of the type [Mn(κ^2^-L)(CO)_3_X]^+^, where L is a noninnocent bidentate ligand and X is a monodentate ligand. These bidentate ligands commonly bind to the metal atom through N and/or C atoms, but the L convention is kept for generality. It should also be noted here that precatalysts of the type [Mn(κ^3^-L)(CO)_2_X], where L is a tridentate noninnocent ligand, have been reported,^5^ albeit for illustrative purposes, bidentate ligands will be used throughout this paper. Upon exposure to reducing potentials, the precatalyst gains electrons and loses its monodentate ligand, leading to the formation of the active complex [Mn(κ^2^-L)(CO)_3_]^−^. This occurs either through the formation of a [Mn(κ^2^-L)(CO_3_)]_2_ dimer, or the formation of a [Mn(κ^2^-L)(CO)_3_]* radical.^6^ Dimerization has been shown to increase the overpotential for the formation of the catalytically active [Mn(κ^2^-L)(CO)_3_]^−^ complex,^7^ and several studies have focused on introducing bulky ligands in an attempt to try and inhibit dimerization.^7−9^ Catalysis of CO_2_ by the active complex typically follows either a “protonation-first” or “reduction-first” mechanism,^10,11^ as shown in Figure 1.

The active catalyst (A) can follow several different pathways to catalyze the reduction of CO_2_ to CO. Depending on the reaction conditions, either H^+^ or CO_2_ can react with A to form the [Mn(κ^2^-L)(CO)_3_H] complex (B) or [Mn(κ^2^-L)(CO)_3_CO_2_]^−^ complex (C), respectively. Further reaction of H^+^ with B will lead to the evolution of H_2_. As H_2_ evolution and CO_2_ reduction reactions occur at similar potentials, it is important to consider faradaic losses from H_2_ evolution when evaluating homogeneous catalysts for the reduction of CO_2_. Subsequently, C is rapidly protonated to form a stable [Mn(κ^2^-L)(CO)_3_COOH] intermediate (E).^12^

Complex E is then further reduced following either a “protonation-first” or “reduction-first” pathway. In the protonation-first mechanism (E–G–H), E is protonated and loses water to form [Mn(κ^2^-L)(CO)_4_]^+^ (G), which is then reduced to [Mn(κ^2^-L)(CO)_4_] (H). In the reduction-first mechanism (E–F–H), E is reduced to form [Mn(κ^2^-L)(CO)_3_COOH]^−^ (F), which is then protonated to form [Mn(κ^2^-L)(CO)_4_] (H).

Complex C can also be reduced again, forming the dianion complex [Mn(κ^2^-L)(CO)_3_CO_2_]^2–^ (D) at sufficiently negative potentials, as evident from DFT calculations.^12^ However, this pathway (C–D–F) has only been reported to occur in the presence of weak acids where the free energy of forming E is unfavorable.^13,14^ With a sufficiently strong acid, Mn–carbonyl complexes can be expected to follow either the protonation-first or reduction-first pathways.

A key difference between Mn–carbonyl complexes and other molecular CO_2_ reduction catalysts is that the former require the presence of an acid (proton donor) to be catalytically active in almost all cases.^12,15^ These are usually weak Brønsted acids so as not to promote H_2_ evolution (e.g., H_2_O, trifluoroethanol (TFE), phenol, or methanol).^16^ The concentration and type of acid play an important role in the activity of the Mn–carbonyl complex. In addition to protonating key intermediates, acids have been shown to stabilize CO_2_ binding through intermolecular hydrogen bonding and second-sphere ligand interactions.^12,17−19^ The acid can also greatly increase the kinetics of an otherwise slow electron-transfer step by coupling it with a proton transfer in a proton-coupled electron-transfer (PCET) step.^20^ In the absence of a proton source, CO_2_ reduction can still occur via reductive disproportionation, which affords CO and CO_3_^2–^, or via the formation of oxalate (C_2_O_4_^2–^). Even though this is rare among Mn–carbonyl catalysts, reductive disproportionation has been reported for some Mn–carbonyl catalysts under aprotic conditions when the Brønsted acid is replaced by a Lewis acid.^7,8,16^ Some Mn–carbonyl catalysts are able to reduce CO_2_ under protic and aprotic conditions, whereby the reported turnover frequencies (TOFs) are significantly higher under protic conditions.^21^

## Model Design Considerations

### Data Acquisition of Mn–Carbonyl Complexes

Cyclic voltammograms for 55 experimentally synthesized Mn–carbonyl complexes were gathered from the literature to extract figures of merit that quantify their activity. Enhancements in catalytic activity for these Mn–carbonyl complexes have been reported by the addition of electron-donating or -withdrawing groups,^22^ intramolecular proton donors,^23,24^ bulky substituents,^7,8^ and additional ligands that change the first coordination sphere of Mn.^21,25,26^ The Mn–carbonyl complexes can be classified into three categories depending on the coordinating ligand: bipyridine derivatives, di-imine-based complexes, and *N*-heterocyclic carbenes. The full list of Mn complexes explored in this study is given in Table 1.^27,28,30,32−45^

**Table 1:**
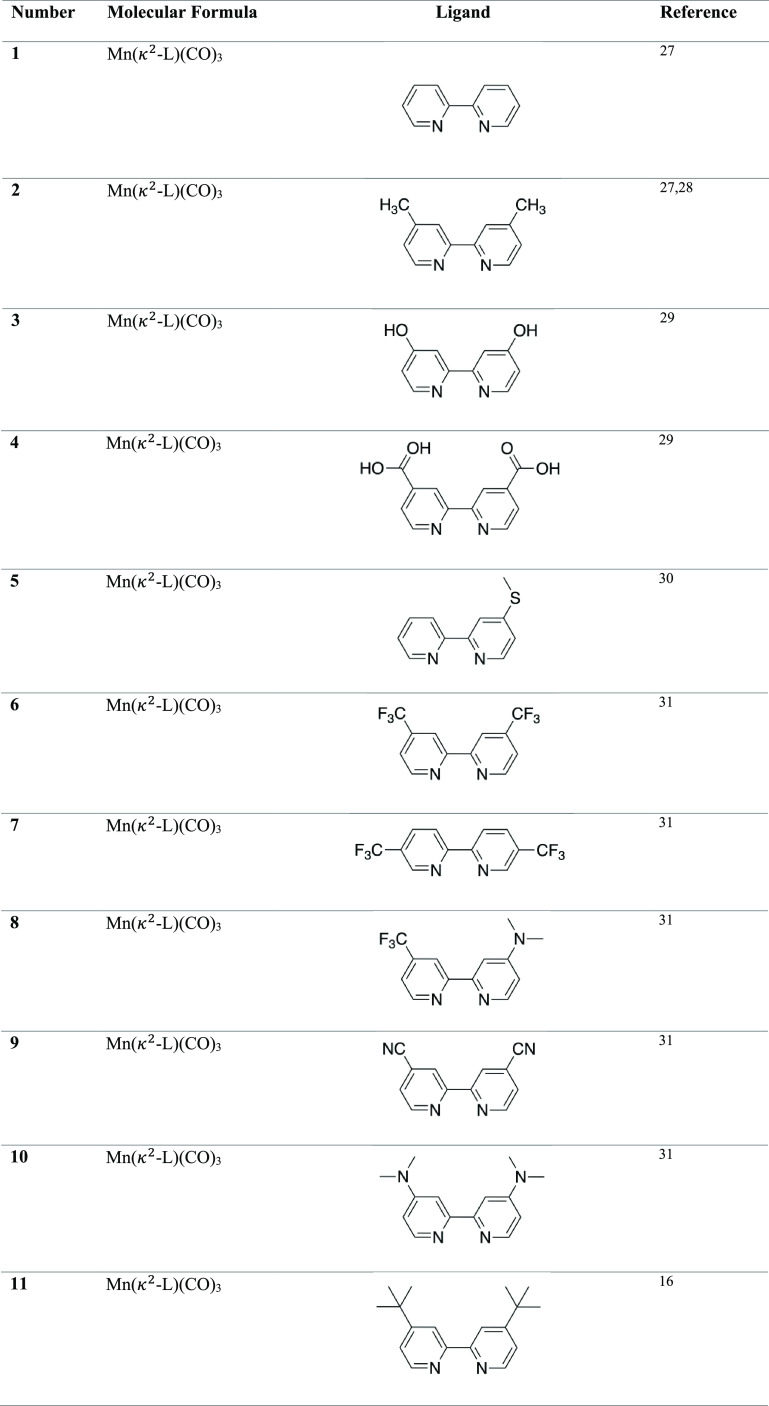

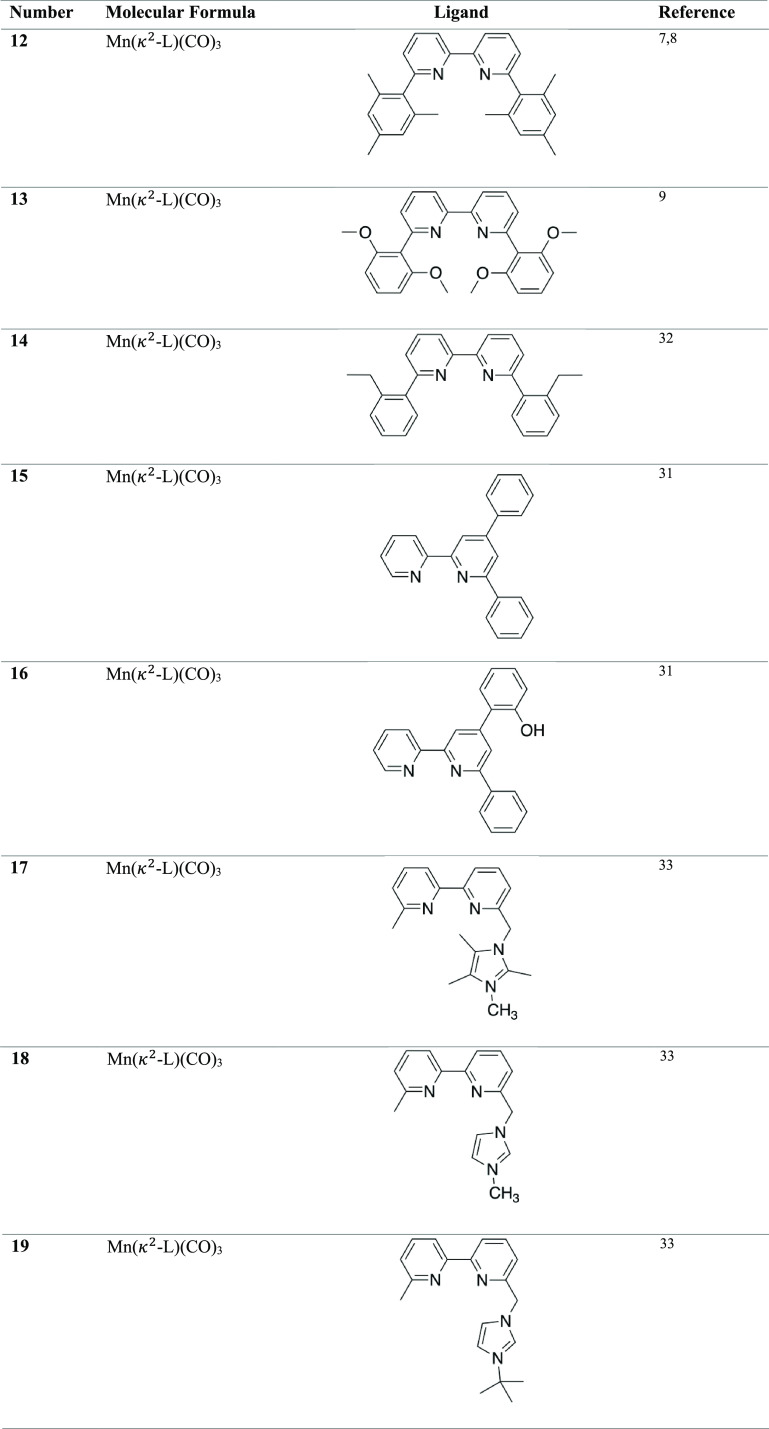

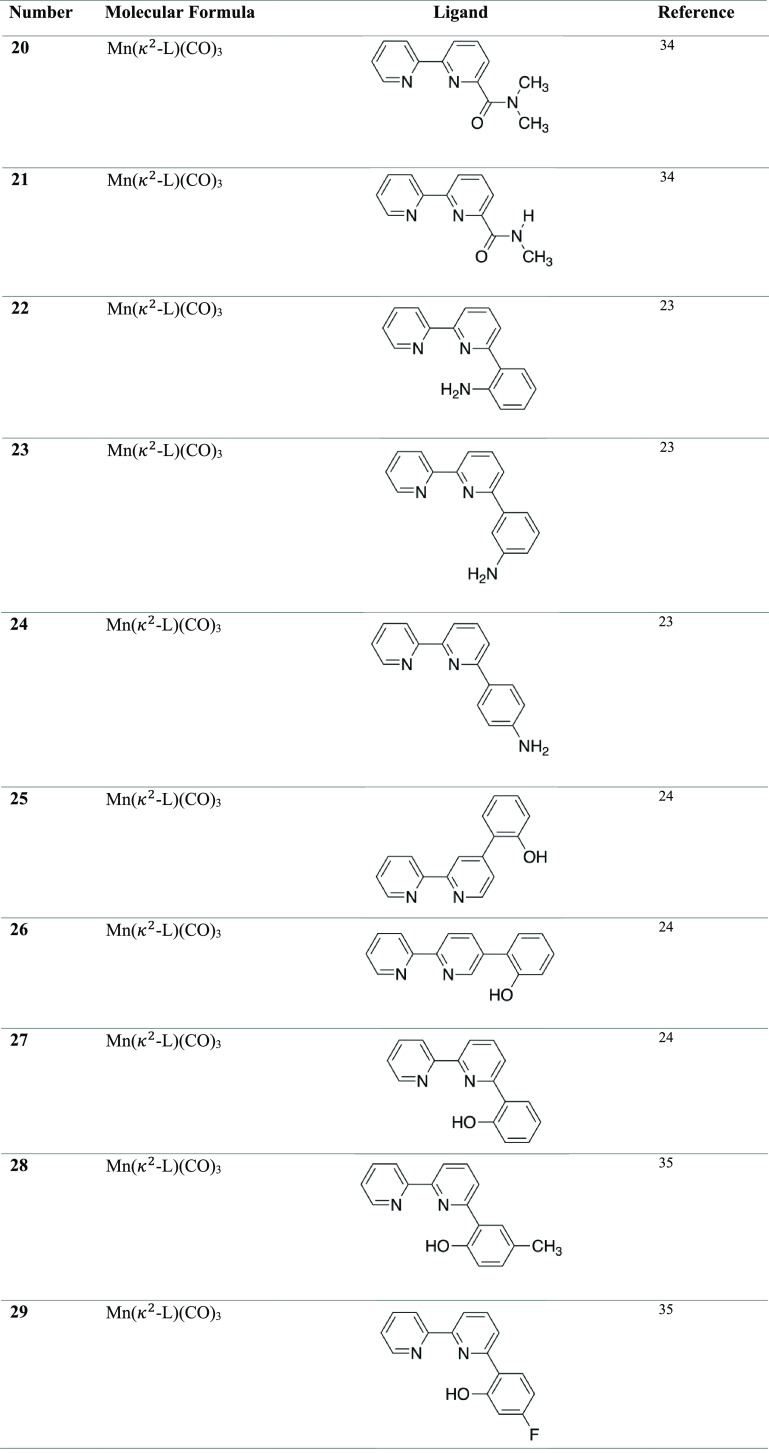

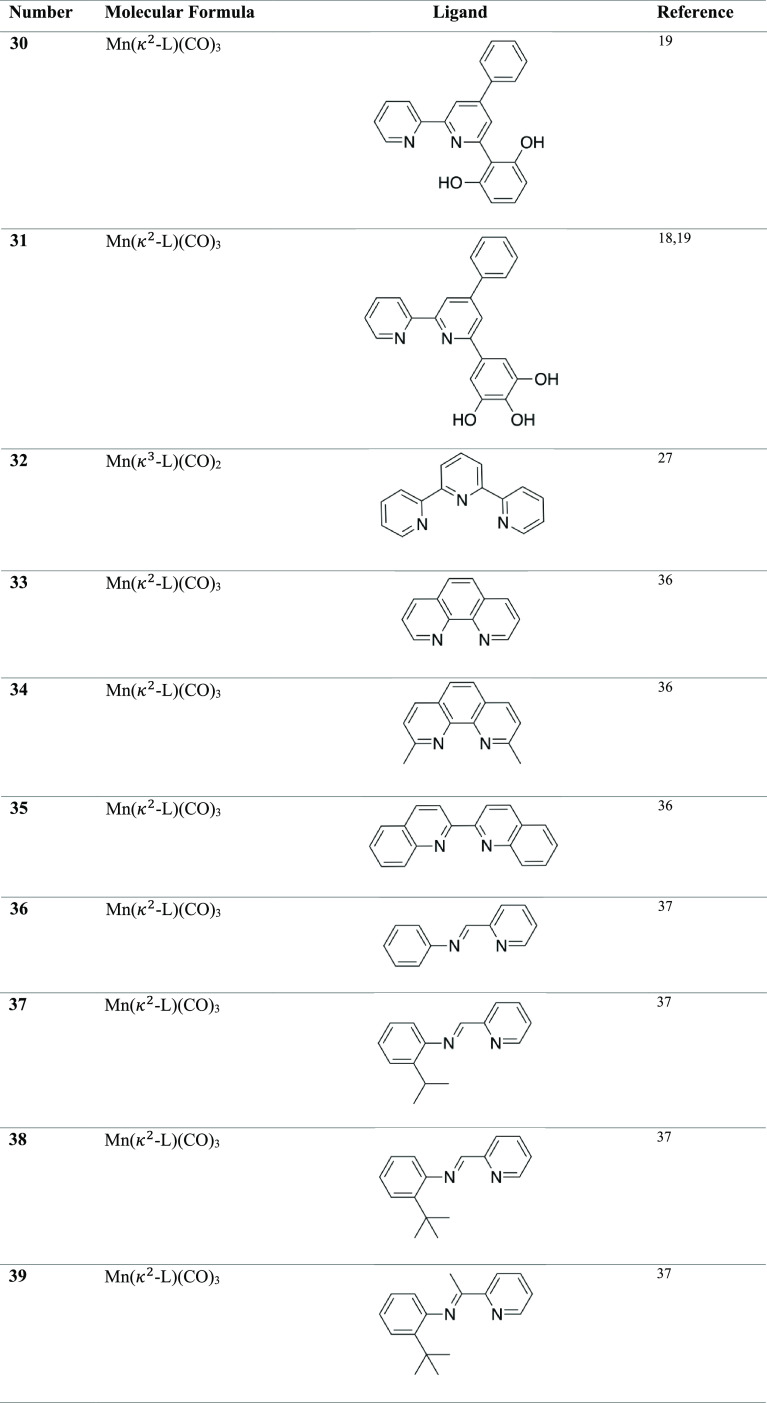

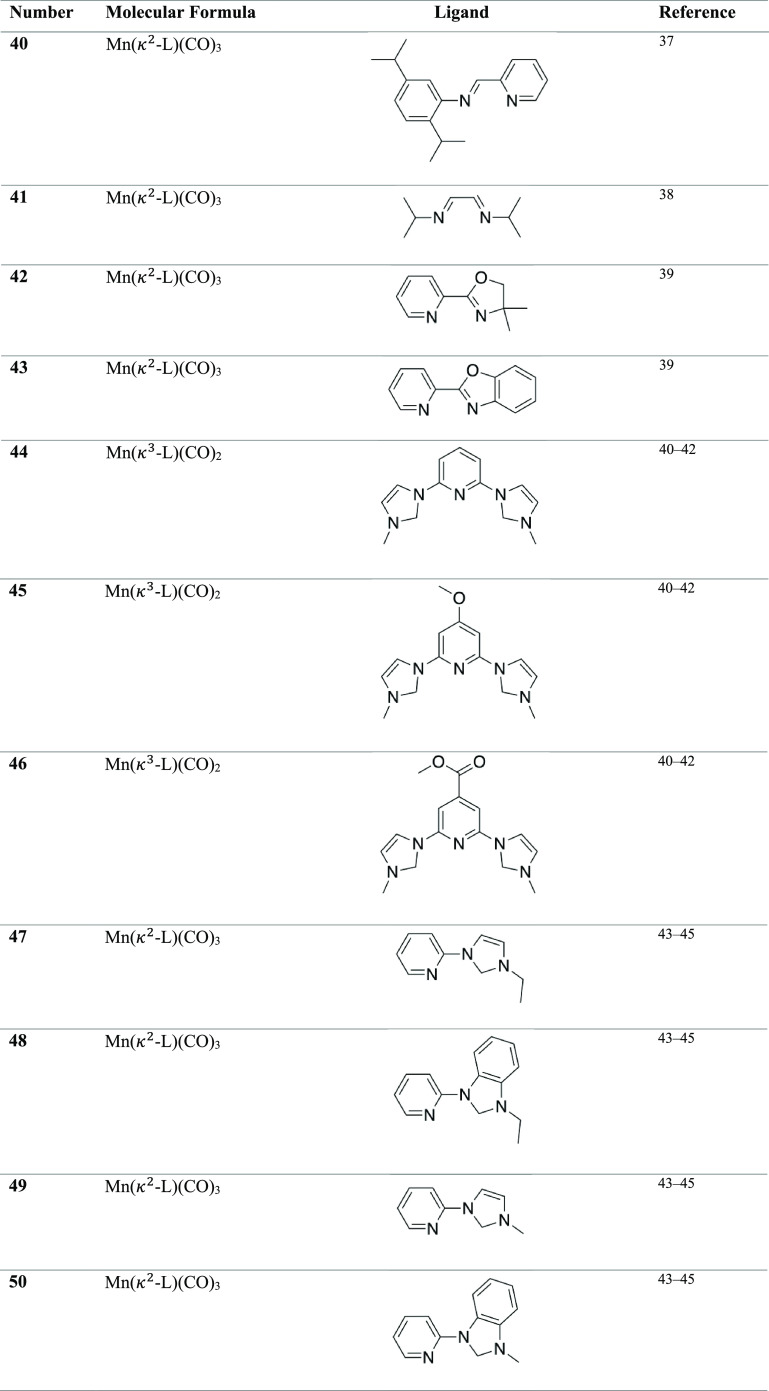

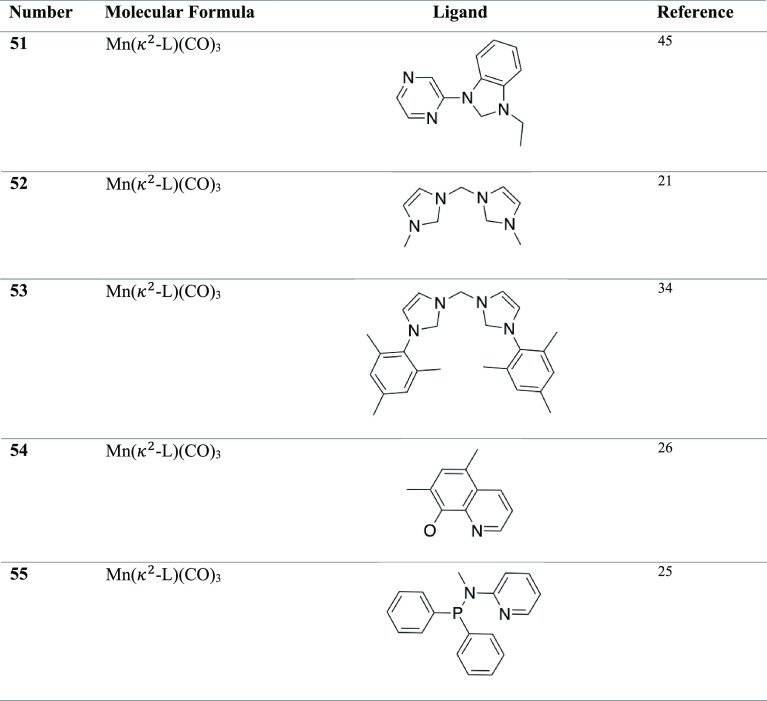
55 Mn–Complexes Used to Explore Correlations between Structure and Electrocatalytic Activity^a^

a Complexes **1**–**43** coordinate to Mn via N atoms, complexes **44**–**51** coordinate to Mn via N and C atoms, complexes **52**–**53** coordinate to Mn via C atoms, complex **54** via N and O atoms, and complex **55** via N and P atoms.

### Defining Figures of Merit for Electrocatalytic Performance

Objective metrics for evaluating the performance of molecular electrocatalysts are necessary to accurately compare catalysts. Historically, parameters to describe electrocatalytic activity for molecular catalysts have been reported under different conditions and with different standards, making comparison challenging.^46^ Thus, benchmarking the activity of molecular catalysts requires a systematic redefinition and reevaluation of these parameters. In this section, we will define three figures of merit that will be used for the evaluation of the Mn–carbonyl molecular catalysts. These are the overpotential (η), maximum turnover frequency (TOF_max_), and faradaic efficiency for the formation of CO.

The overpotential (η) is defined as the difference between the thermodynamic potential required for CO_2_ to be reduced to CO and the potential at which the catalyst is operated (eq 1). These are both a function of the type of solvent used and the p*K*_a_ of the solution. As the operating potential is not restrictive to any particular feature of the catalyst, we define the operating potential as the half-wave potential *E*_cat/2_.
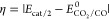
1*E*_cat/2_ is loosely defined as the potential where the catalytic wave reaches half of its maximum current; however, this definition is only valid under pure kinetic conditions,^46^ which only occur when (i) there is no substrate depletion nearby, (ii) there are no side reactions in which the cyclic voltammogram is S-shaped, and (iii) the forward and reverse waves overlap completely. These conditions do not occur in all Mn–carbonyl catalysts that are investigated within the present study, and therefore, the half-wave potential is taken as the inflection point of the forward catalytic wave (Figure S1).

The maximum turnover frequency (TOF_max_) is a measure of the activity of a catalyst under a certain set of reaction conditions and describes the maximum number of catalytic cycles occurring per unit of time. The TOF of a catalyst depends on the number of molecules of catalyst in the active state, which is a potential-dependent parameter. TOF_max_ is the turnover frequency at potentials that are sufficiently negative that 100% of the catalyst molecules are in their active reduced state. Foot-of-the-wave (FOTW) analysis is used to calculate TOF_max_ from cyclic voltammograms (Figures S2–S4).

It should be kept in mind that TOF_max_ does not account for differences in CO_2_ concentration and the nature of the proton donor between different catalytic setups. The definition of this dataset, which contains only 55 Mn–carbonyl complexes, was designed to account for this shortcoming and allow comparisons between TOF_max_ values for two reasons. First, all Mn–carbonyl complexes considered were measured in acetonitrile under CO_2_ saturation, and the solubility of CO_2_ in acetonitrile is the same for all catalysts. Second, small differences in the concentration of proton donors should not affect the qualitative trends that are discussed here. Catalysts in which the type of proton donor is different, most commonly H_2_O or trifluoroethanol, are treated as separate datasets and independent conclusions are drawn for each.

The turnover frequency at zero overpotential (TOF_0_) is a metric designed to evaluate catalytic performance by considering the impact of both TOF_max_ and the overpotential. To calculate TOF_0_, the TOF_max_ is extrapolated to the thermodynamic potential for CO_2_ reduction (*E*_CO_2_/CO_^0^) under the relevant conditions. This is described by eq 2, where η = (*E*_CO_2_/CO_^0^ – *E*_cat/2_).^46^
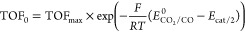
2As TOF_0_ strongly depends on the value of *E*_CO_2_/CO_^0^, it is not a good metric for comparing catalyst systems that differ with respect to type or concentration of substrate. When evaluating molecular catalysts for CO_2_ reduction with selectivity for different products, differences in thermodynamic potential may skew the conclusions gained from TOF_0_. Also, changing the concentration of substrate (e.g., CO_2_) will affect the thermodynamic potential via the Nernst equation. Mn–carbonyl electrocatalysts present a good-use case for TOF_0_ because they are operated under the same CO_2_ concentration and are, aside from a few exceptions, selective toward one product (CO/H_2_O).

### Features and Descriptor Generation

Understanding what properties of the Mn–carbonyl complex result in a high TOF_max_ and low overpotential is crucial for advancing the development of molecular electrocatalysts by rational design approaches. Table 2 describes 15 features that were used to obtain a physical understanding of what features of the ligand affect TOF_max_ and the overpotential. The first three features (Δ*G*_1_, Δ*G*_2_, Δ*G*_3_) represent energies for specific mechanistic steps in Figure 1. The remaining features represent the energies of the highest occupied molecular orbital (HOMO) and the lowest unoccupied molecular orbital (LUMO) orbitals and partial charges for complexes A, B, C, and H in Figure 1. Features of the Mn–Mn dimer complex are not included, and we assume that the electronic properties of the dimer will scale with the features of the monomeric complexes. The relative importance of these features in determining electrocatalytic properties will give insight into what mechanistic steps are most relevant. The mechanistic insights gained from this analysis rely on the assumption that scaling relations are present between the electronic descriptors in Figure 1 and transition-state energies. By restricting our focus to only Mn–carbonyl catalysts with similar electronic properties, such scaling relations are likely to be present, as in previous studies of homogeneous catalysts.^47^

**Table 2:** Primary Features Included in SISSO Models^a^

feature label	description
Δ*G*_1_	free energy for CO_2_ binding

Δ*G*_2_	free energy for H^+^ dissociation

Δ*G*_3_	free energy for CO dissociation

*E*_1H_	energy of the HOMO orbital of [Mn(κ^2^-L)(CO)_3_H]^0^
*E*_1L_	energy of the LUMO orbital of [Mn(κ^2^-L)(CO)_3_H]^0^
*E*_2H_	energy of the HOMO orbital of [Mn(κ^2^-L)(CO)_3_CO_2_]^−^
*E*_2L_	energy of the LUMO orbital of [Mn(κ^2^-L)(CO)_3_CO_2_]^−^
*E*_3H_	energy of the HOMO orbital of [Mn(κ^2^-L)(CO)_3_]^−^
*E*_3L_	energy of the LUMO orbital of [Mn(κ^2^-L)(CO)_3_]^−^
*E*_4H_	energy of the HOMO orbital of [Mn(κ^2^-L)(CO)_4_]^0^
*E*_4L_	energy of the LUMO orbital of [Mn(κ^2^-L)(CO)_4_]^0^
δ_1_	mulliken charge on Mn in [Mn(κ^2^-L)(CO)_3_H]^0^
δ_2_	mulliken charge on Mn in [Mn(κ^2^-L)(CO)_3_CO_2_]^−^
δ_3_	mulliken charge on Mn in [Mn(κ^2^-L)(CO)_3_]^−^
δ_4_	mulliken charge on Mn in [Mn(κ^2^-L)(CO)_4_]^0^

a all energies are given in kJ/mol.

The sure independence screening and sparsifying operator (SISSO)^48^ was applied to the dataset of the 55 Mn–carbonyl electrocatalysts, and the 15 primary features from Table 2 were fed into the SISSO framework. These primary features are used as the starting point for SISSO, and a feature space is constructed by recursively applying a combination of mathematical operators {*I*,+, −, ×,÷,|−|}.

SISSO is a very useful method for understanding the relationship between the features of a catalyst and its turnover frequency, overpotential, and selectivity. The mathematical models generated by SISSO allow for easy inspection and interpretation, and they can be used to test the generalizability of the model. A dimensional analysis was performed so that only meaningful combinations of features and operators were kept. In this case, all energies have units of kJ/mol, and combinations adding an energy to a charge are not considered given that the resulting units would be unphysical. Even with a relatively small number of catalysts and a large number of highly correlated features, the performance of SISSO does not suffer.^48^ The analysis was restricted to one-dimensional models with a maximum of three features to find simple descriptors that are physically interpretable. However, stronger correlations can be observed toward higher-dimensional and more complex models.

Further, the dataset containing 55 Mn–carbonyl molecular electrocatalysts was divided into 16 subgroups to understand the applicability of the SISSO models to datasets containing different ligand types, solvent types, and solvent concentrations. Three main groups of ligands were identified in the dataset: bipyridine ligands (bpy), di-imine ligands apart from bipyridine (nn), and *N*-heterocyclic carbenes (NHCs). Data were further stratified by proton-donor type (H_2_O or TFE) and concentration because the nature of the proton donor affects catalytic figures of merit.^12,16^ Specific correlations with SISSO models within a single ligand type and general correlations that encompass multiple ligand types were identified and are discussed in the following section.

## Results and Discussion

SISSO models are trained separately on the three experimental parameters of catalytic activity: TOF_max_, overpotential, and TOF_0_. The top three SISSO models for each data subset are listed in Tables S1–S3. There is no single best model that predicts catalytic activity across all groups of ligands and solvents, albeit important ligand properties could be inferred by looking at the prevalence of certain features in the SISSO models.

### Descriptors for TOF_max_

The highest-performing two-parameter SISSO models for TOF_max_ are given in Table 3.

**Table 3:** The Three Best SISSO Models for TOF_max_ Trained on Datasets Split According to Solvent and Ligand Inclusion Criteria^a^

model inclusion criteria			SISSO models		
ligand type	solvent type	solvent concentration (M)	SISSO 1	SISSO 2	SISSO 3
bpy	H_2_O	2.7	*E*_4H_ × δ_4_ (0.75)	*E*_3H_ × δ_4_ (0.71)	*E*_2H_ × δ_4_ (0.69)
bpy	H_2_O	0.79–7.2	*E*_4H_ × δ_4_ (0.81)	*E*_1H_ × δ_4_ (0.78)	δ_4_ (0.75)
nn	H_2_O	2.7	Δ*G*_1_ – *E*_2H_ (0.70)	*E*_4H_ – *E*_2H_ (0.66)	|*E*_3H_ – *E*_4H_| (0.64)
nn	H_2_O	0.17–7.2	*E*_4H_ × δ_1_ (0.66)	*E*_4H_ × δ_4_ (0.65)	δ_4_ + δ_3_ (0.65)
nhc	H_2_O	0.55–2.7	*E*_4H_ × δ_1_ (0.95)	*E*_4H_/Δ*G*_3_ (0.95)	*E*_4H_ × δ_4_ (0.93)
all	H_2_O	2.7	Δ*G*_3_ – *E*_4H_ (0.49)	Δ*G*_1_ × *E*_4L_ (0.47)	*E*_3H_ *– E*_2H_ (0.46)
all	H_2_O	0.17–7.2	*E*_1L_ × δ_1_ (0.56)	*E*_2L_ × δ_1_ (0.53)	*E*_1L_ × δ_4_ (0.52)
bpy	TFE	0.5–1.9	*E*_3H_ – *E*_2L_ (0.99)	*E*_2H_ – *E*_2L_ (0.99)	*E*_1H_ (0.97)
nn	TFE	0.5–2.5	Δ*G*_1_ – *E*_1L_ (0.72)	*E*_4L_ × δ_4_ (0.68)	*E*_2H_/*E*_3H_ (0.67)
all	TFE	0.5–3.1	*E*_3H_ – *E*_2H_ (0.78)	δ_4_/Δ*G*_3_ (0.74)	*E*_4H_ × δ_4_ (0.74)

a the Pearson correlation coefficient of each model is shown in parentheses after the models.

**Figure 2:**
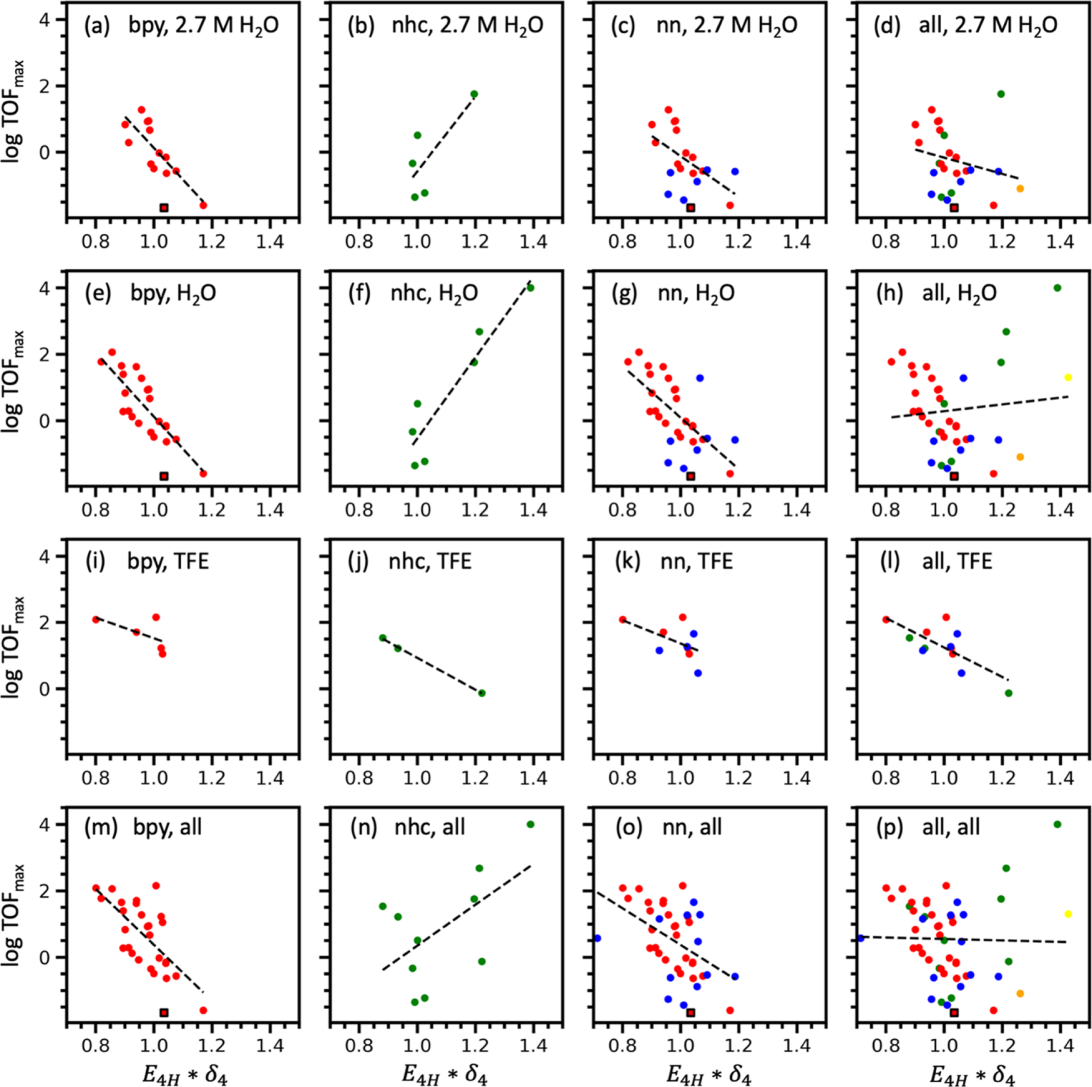
Performance of SISSO model 1 (*E*_4H_ × δ_4_) for the prediction of TOF_max_ of Mn–carbonyl complexes. Each plot represents different inclusion criteria for ligand type, solvent, and solvent concentration: (a) bpy ligands in 2.7 M H_2_O, (b) NHC ligands in 2.7 M H_2_O, (c) nn ligands in 2.7 M H_2_O, (d) all ligands in 2.7 M H_2_O, (e) bpy ligands in any concentration of H_2_O, (f) all NHC ligands in any concentration of H_2_O, (g) nn ligands in any concentration of H_2_O, (h) all ligands in any concentration of H_2_O, (i) bpy ligands in any concentration of TFE, (j) NHC ligands in any concentration of TFE, (k) nn ligands in any concentration of TFE, (l) all ligands in any concentration of TFE, (m) bpy ligands in all solvents types, (n) NHC ligands in all solvent types, (o) nn ligands in all solvent types, (p) all ligands in all solvent types. Outliers are labeled using a square marker with black edges and are excluded from the linear fit shown.

Features containing information about the [Mn(κ^2^-L)(CO)_4_]^0^ intermediate correlate highest to TOF_max_ across different subgroups in the dataset. The model *E*_4H_ × δ_4_ is prevalent among top-performing SISSO models across multiple subgroups in the dataset as shown in Table 3. *E*_4H_ × δ_4_ was plotted as a function of TOF_max_ to assess its predictive performance across broad and specific subgroups (Figure 2). The *x*-axis is normalized by the value of *E*_4H_ × δ_4_ for bipyridine complex **1**. The colors of the data points in Figure 2 correspond to the ligand types of the Mn–carbonyl complex, i.e., red = bpy, green = NHC, blue = nn, orange = P−N-coordinating ligands, and yellow = O–N-coordinating ligands.

The organization of Figure 2 is such that one can see under what conditions the predictive ability of the model breaks down. The most controlled subgroups are given in the top left quadrant of Figure 2, while the most general subgroups are given in the bottom right quadrant. Figure 2a–d represents individual ligand types that are specifically in 2.7 M H_2_O. The model (*E*_4H_ × δ_4_) exhibits high correlation coefficients with Mn–carbonyl complexes of a single ligand type under these conditions because any variation in TOF_max_ must be explained by the intrinsic properties of the ligand. Figure 2e–h builds on the first row by including Mn–carbonyl complexes that have been reported in any concentration of H_2_O. Figure 2i–l represents Mn–carbonyl complexes reported using TFE as the solvent at any concentration. Models for different ligand types in TFE are less certain on account of the limited number of data points in this subgroup. Finally, Figure 2m–p represents Mn–carbonyl complexes in all solvent types (H_2_O, TFE, and phenol). Models for these subgroups tend to exhibit weaker correlation coefficients due to the effect of the solvent. While the *E*_4H_ × δ_4_ model is robust for predicting TOF_max_ across a single ligand type in a single solvent, it breaks down when applied to many ligand types in different solvent environments.

TOF_max_ is negatively correlated with (*E*_4H_ × δ_4_) for nn ligands. The strongest correlations are observed for bpy ligands in H_2_O with a Pearson correlation coefficient of *r* = 0.75 and *r* = 0.81 for Figure 2a,e, respectively. The predictive ability of (*E*_4H_ × δ_4_) can be rationalized by looking at the last step of the electrocatalytic pathway as species H connects to species A in Figure 1. When looking at a series of bpy ligands, a lower value of (*E*_4H_ × δ_4_) results in a higher TOF_max_. Because both *E*_4H_ and δ_4_ are negative numbers, their product is positive. Low values of (*E*_4H_ × δ_4_) correspond to a high energy level of the HOMO orbital and a less negative partial charge. This understanding could be used to design and screen for catalysts with a favorable value of (*E*_4H_ × δ_4_) to optimize TOF_max_. For example, our data shows that adding electron-donating groups to bipyridine ligands increases the energy of the [Mn(κ^2^-L)(CO)_4_]^0^ HOMO orbital (*E*_4H_), leading to lower values of the descriptor (*E*_4H_ × δ_4_). This observation is confirmed by a previous study showing that electron-donating groups increase TOF_max_ at the expense of a higher overpotential.^31^

A high-energy HOMO orbital (*E*_4H_) corresponds to a less stable [Mn(κ^2^-L)(CO)_4_]^0^ intermediate with a lower activation energy for the CO-dissociation step. In the same way, a less negative partial charge δ_4_ on the Mn atom suggests that the [Mn(κ^2^-L)(CO)_4_]^0^ intermediate will have a lower barrier to gain an electron in the following reduction step that reforms the active [Mn(κ^2^-L)(CO)_3_]^−^ complex and releases CO. Mechanistically, this suggests that the electron-mediated dissociation of CO may be important in the reduction of CO_2_ using Mn–carbonyl complexes with bpy-based ligands. Previous evidence points to C–(OH) bond cleavage to form the [Mn(κ^2^-L)(CO)_4_]^0^ complex being the rate-determining step for di-imine ligands,^49^ and it is also possible that a catalyst with a low value of (*E*_4H_ × δ_4_) represents a [Mn(κ^2^-L)(CO)_4_]^0^ intermediate which is easily formed.

One notable outlier in Figure 2a is the dihydroxy-substituted bpy ligand in complex **3** (Table 1). Despite having an *E*_4H_ × δ_4_ value of 1.04, it has a considerably lower TOF_max_ than that predicted by the linear fit (log TOF_max_ = −1.63). The top-performing SISSO models with complex **3** removed are given in Table S1. The redox behavior of this complex differs from that typically seen for Mn–carbonyl catalysts, with four redox peaks in the cyclic voltammogram.^29^ It also shows a much larger current enhancement in anhydrous acetonitrile, which decreases upon the addition of H_2_O as a proton donor; this behavior stands in contrast to that of all other Mn–carbonyl complexes.^29^ Complex **3** also exhibits a very low faradaic efficiency for CO formation (FE_CO_ = 6%) and is more selective toward H_2_ evolution (FE_H_2__ = 45%).^29^ It is clear that this complex shows very different electrocatalytic behavior from complexes with other bipyridine ligands and Mn–carbonyl complexes in general; it seems feasible to assume that this complex follows a different mechanism.

The NHC-ligated complexes with H_2_O as a proton donor follow a different trend than the complexes coordinated by bpy ligands. The NHC ligands tend to have more negative values of δ_4_ which reflect the stronger electron-donating properties of NHC ligands. TOF_max_ is positively correlated with *E*_4H_ × δ_4_ in the corresponding Mn–carbonyl complexes (Figure 2b,f). This means that a more stable [Mn(κ^2^-L)(CO)_4_]^0^ intermediate gives rise to higher values of TOF_max_ when L is a NHC ligand and suggests that electron-mediated dissociation is not the rate-determining step. This is consistent with a previous DFT study that has shown that the barriers for CO liberation can be expected to be smaller than the barriers for C–O bond cleavage for Mn–carbonyl catalysts coordinated by NHC ligands.^50^ Interestingly, the CO-dissociation step was predicted to occur via a pyridine-dissociation mechanism, which differs from the mechanism in bpy-based Mn–carbonyl catalysts.^50^ The trend for NHC-coordinated complexes appears to reverse with TFE as the proton donor (Figure 2j), albeit conclusions based on only three data points should be considered cautiously.

### Descriptors for the Overpotential

A similar analysis using SISSO to generate predictive models for the overpotential was carried out, and the top-performing models are given in Table 4 for each subgroup.

**Table 4:** The Best Three SISSO Models for the Overpotential Trained on Datasets Split According to Solvent and Ligand Inclusion Criteria^a^

model inclusion criteria			SISSO models		
ligand type	solvent type	solvent concentration (M)	SISSO 1	SISSO 2	SISSO 3
bpy	H_2_O	2.7	δ_4_/Δ*G*_3_ (0.81)	δ_4_/*E*_3H_ (0.79)	δ_4_/*E*_1H_ (0.77)
bpy	H_2_O	0.79–7.2	*E*_1H_ + *E*_2H_ (0.61)	*E*_3L_ *– E*_4L_ (0.61)	*E*_3L_ – *E*_1L_ (0.61)
nn	H_2_O	2.7	*E*_1H_ + *E*_2H_ (0.67)	*E*_1H_ + *E*_3L_ (0.61)	*E*_1H_ (0.56)
nn	H_2_O	0.17–7.2	*E*_1H_ + *E*_2H_ (0.59)	*E*_2H_ (0.52)	*E*_1H_ (0.43)
nhc	H_2_O	0.55–2.7	*E*_4H_ *– E*_4L_ (0.98)	Δ*G*_2_/δ_4_ (0.98)	Δ*G*_3_ (0.97)
all	H_2_O	2.7	Δ*G*_3_ + *E*_2H_ (0.66)	Δ*G*_3_ + *E*_1H_ (0.61)	Δ*G*_3_ (0.56)
all	H_2_O	0.17–7.2	Δ*G*_3_ + *E*_2H_ (0.54)	*E*_4H_ – *E*_1L_ (0.51)	*E*_2H_ (0.47)
bpy	TFE	0.5–1.9	*E*_3H_ × *E*_4L_ (0.97)	*E*_1L_ × *E*_4H_ (0.97)	*E*_1L_(0.96)
nn	TFE	0.5–2.5	*E*_3H_ + *E*_4H_ (0.98)	Δ*G*_3_ × *E*_1L_ (0.97)	*E*_1L_ (0.94)
all	TFE	0.5–3.1	Δ*G*_1_ – *E*_3H_ (0.97)	*E*_2L_/Δ*G*_2_ (0.97)	*E*_3H_ + *E*_4H_ (0.95)

a the Pearson correlation coefficient of each model is shown in parentheses after the models.

**Figure 3:**
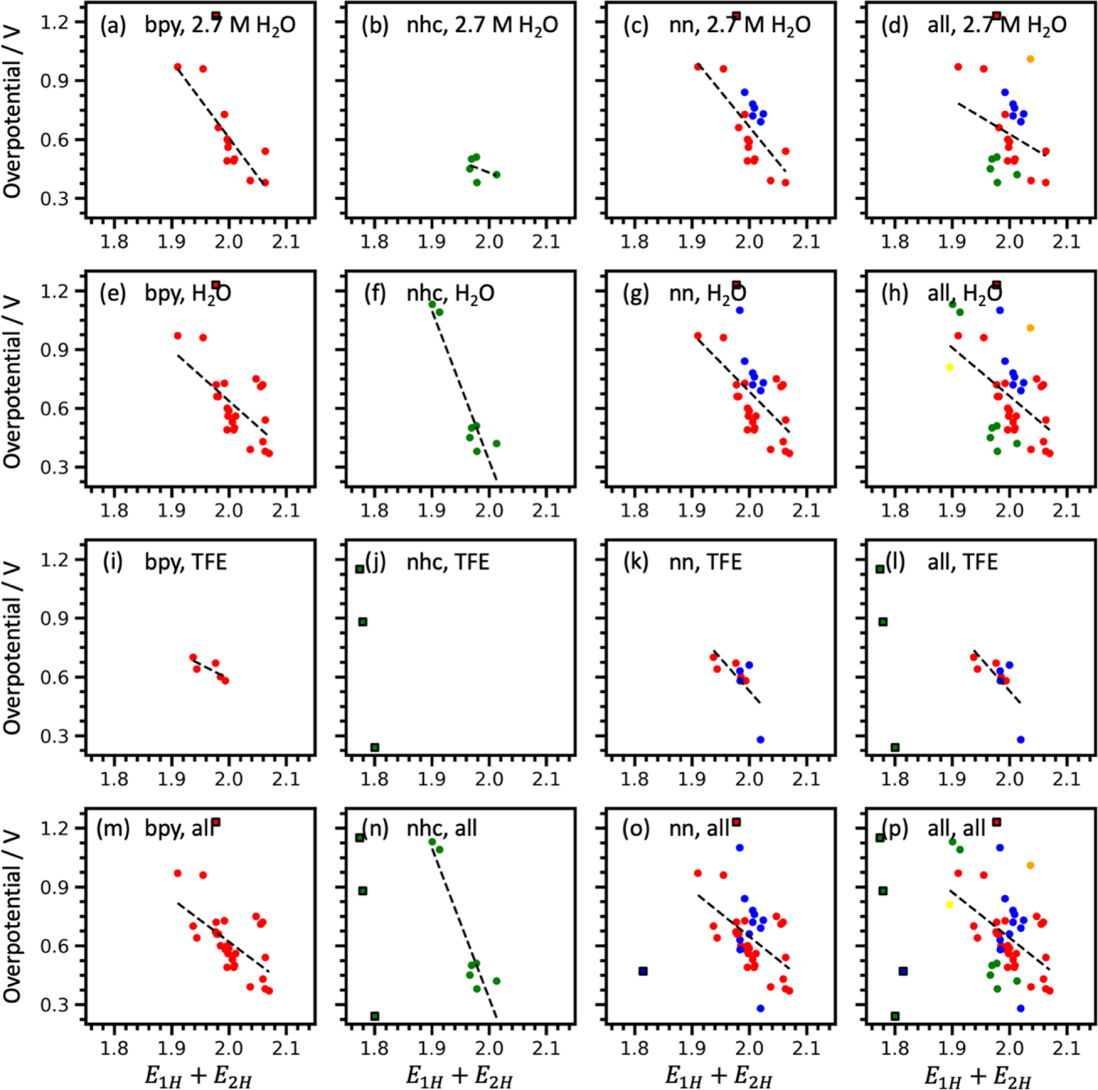
Performance of SISSO Model 2 (*E*_1H_ + *E*_2H_) for the prediction of the catalytic overpotential of Mn–carbonyl complexes. Each plot represents different inclusion criteria for ligand type, solvent, and solvent concentration: (a) bpy ligands in 2.7 M H_2_O, (b) NHC ligands in 2.7 M H_2_O, (c) nn ligands in 2.7 M H_2_O, (d) all ligands in 2.7 M H_2_O, (e) bpy ligands in any concentration of H_2_O, (f) all NHC ligands in any concentration of H_2_O, (g) nn ligands in any concentration of H_2_O, (h) all ligands in any concentration of H_2_O, (i) bpy ligands in any concentration of TFE, (j) NHC ligands in any concentration of TFE, (k) nn ligands in any concentration of TFE, (l) all ligands in any concentration of TFE, (m) bpy ligands in all solvents types, (n) NHC ligands in all solvent types, (o) nn ligands in all solvent types, (p) all ligands in all solvent types. Outliers are labeled using a square marker with black edges and are excluded from the linear fit shown.

To understand the predictive ability of these features in more detail, the catalytic overpotential was plotted as a function of the best-performing SISSO model *E*_1H_ + *E*_2H_ for 16 different subgroups of ligands, solvents, and solvent concentrations in Figure 3. These models were normalized by the value of *E*_1H_ + *E*_2H_ for the bipyridine-ligated complex **1** for readability.

The negative trends for all plots in Figure 3 are evidence that the value of *E*_1H_ + *E*_2H_ is negatively correlated with the overpotential for all ligand and solvent types. These HOMO orbital energies, *E*_1H_ and *E*_2H_, each have negative values (Table S6). Thus, higher values of *E*_1H_ + *E*_2H_ correspond to more negative HOMO orbital energies and therefore more stable [Mn(κ^2^-L)(CO)_3_H]^0^ and [Mn(κ^2^-L)(CO)_3_CO_2_]^−^ intermediates. When these intermediates are more stable, a lower overpotential is required to initiate the electrocatalysis of CO_2_. One interpretation of this result would be that the overpotential of the catalytic reduction depends on the binding of H^+^ and CO_2_ to the Mn–carbonyl catalyst. At high values of *E*_1H_ + *E*_2H_, Mn has a higher affinity for protons and CO_2_, and the protonation and/or CO_2_ binding to Mn triggers the subsequent catalytic steps. Previously, the electrocatalytic overpotential has been proposed to be the result of the stability of the [Mn–COOH] intermediate (E in Figure 1), and that the reduction of [Mn–COOH] is required for the reduction of CO_2_.^8^ It is also possible that *E*_1H_ + *E*_2H_ correlates negatively with the stability of [Mn–COOH].

The dihydroxy-bipyridine-ligated complex **3** is an outlier in Figure 3 because it has a much higher overpotential (1.22 V) than that predicted by the trendline. As discussed before, complex **3** exhibits much different redox behavior from complexes that carry other bipyridine ligands, which may explain this discrepancy. Similarly, the terpyridine-ligated complex **32** is another notable outlier, as shown in Figure 3o; terpyridine-ligated Mn–carbonyl complex **32** has a much lower catalytic overpotential (0.47 V) than predicted by the trendline. This could be a result of two factors: (1) the terpyridine complex is the only one in our dataset that has been studied with phenol as a proton donor, and (2) terpyridine is a unique ligand type in this dataset as it coordinates to the Mn center via three nitrogen atoms. The top-performing SISSO models with complex **3** and complex **32** removed are given in Table S2. Interestingly, the four Mn–carbonyl complexes **32**, **44**, **45**, and **46** that are well separated in Figure 3p (left) are all coordinated with tridentate ligands.

The trends for overpotential present in Figure 3 hold much better when generalized to different ligand types and solvents than the trends for TOF_max_. Specifically, Figure 3h,o shows that *E*_1H_ + *E*_2H_ is generalizable to different ligand types in H_2_O and nn ligands in H_2_O and TFE, respectively.

### Descriptors for TOF_0_

The ideal electrocatalyst for the reduction of CO_2_ has a high TOF_max_ and a low overpotential, which is characterized by the TOF_0_. The top three one-dimensional SISSO models for TOF_0_ are given in Table 5 for each subgroup of ligand, solvent, and solvent concentration.

**Table 5:** The Three Best SISSO Models for TOF_0_ Trained on Datasets Split According to Solvent and Ligand Inclusion Criteria^a^

model inclusion criteria			SISSO models		
ligand type	solvent type	solvent concentration (M)	SISSO 1	SISSO 2	SISSO 3
bpy	H_2_O	2.7	δ_4_/Δ*G*_3_ (0.86)	δ_4_/*E*_1H_ (0.80)	δ_4_/*E*_3H_ (0.79)
bpy	H_2_O	0.79–7.2	*E*_1H_ + *E*_2H_ (0.61)	δ_4_/*E*_1L_ (0.57)	*E*_2H_ (0.54)
nn	H_2_O	2.7	*E*_1H_ + *E*_2H_ (0.62)	*E*_1H_ + *E*_3L_ (0.54)	*E*_2H_ (0.52)
nn	H_2_O	0.17–7.2	*E*_1H_ + *E*_2H_ (0.59)	*E*_2H_ (0.56)	Δ*G*_1_ + *E*_1H_ (0.50)
nhc	H_2_O	0.55–2.7	*E*_4H_/*E*_4L_ (0.99)	δ_4_/Δ*G*_2_ (0.99)	*E*_1H_ (0.98)
all	H_2_O	2.7	Δ*G*_3_ + *E*_2H_ (0.64)	Δ*G*_3_ (0.52)	*E*_4H_ *– E*_4L_ (0.51)
all	H_2_O	0.17–7.2	Δ*G*_3_ + *E*_2H_ (0.50)	*E*_2H_ (0.46)	*E*_1H_ + *E*_2H_ (0.42)
bpy	TFE	0.5–1.9	*E*_1H_ + *E*_2L_ (0.98)	*E*_4L_ × *E*_2H_ (0.97)	Δ*G*_1_ + *E*_1H_ (0.94)
nn	TFE	0.5–2.5	*E*_3H_ + *E*_4H_ (0.96)	Δ*G*_2_ + *E*_4H_ (0.96)	*E*_1L_ + *E*_2H_ (0.94)
all	TFE	0.5–3.1	Δ*G*_1_ – *E*_1L_ (0.96)	Δ*G*_2_ + *E*_1L_ (0.96)	*E*_1L_ (0.91)

a the Pearson correlation coefficient of each model is shown in parentheses after the models.

High-performing models for TOF_0_ emphasize features similar to models for the catalytic overpotential. This is likely because the range of overpotentials in the dataset is very large, whereas changes in TOF_max_ between individual catalysts are less important for the determination of TOF_0_. *E*_1H_ and *E*_2H_ appear to be important in predicting the TOF_0_ of Mn–carbonyl electrocatalysts. Δ*G*_3_ is also a prominent feature as the models are generalized to different ligand types (Table 5). Complexes **3** and **32** were identified as outliers in the analysis of the models for both TOF_max_, catalytic overpotential, and TOF_0_. To assess their impact on the correlation coefficients of the SISSO models, they were excluded from the SISSO training sets (Table S3); while the top-performing models remain generally the same, the correlation coefficients increase considerably because of this removal.

### Effect of Increasing Feature Complexity of SISSO Models for TOF_0_

Increasing the feature complexity may give better-performing models, albeit it is important to understand the tradeoff between complexity/interpretability and performance. When the maximum number of features in each SISSO model is increased from two to three, the correlation coefficients of the models for TOF_0_ increase, as shown in Table 6.

**Table 6:** The Best Two 3-Feature SISSO Models for TOF_0_ Trained on Datasets Split According to Solvent and Ligand Inclusion Criteria^a^

model inclusion criteria			SISSO models	
ligand type	solvent type	solvent concentration (M)	SISSO 1	SISSO 2
bpy	H_2_O	2.7	2*E*_1H_ + *E*_2H_ (0.91)	*E*_1H_ × δ*G*_1_/δ_4_ (0.89)
bpy	H_2_O	0.79–7.2	Δ*G*_1_ + *E*_1H_ + *E*_4L_ (0.77)	*E*_3H_ – *E*_1H_ *– E*_2H_ (0.77)
nn	H_2_O	2.7	Δ*G*_1_ + *E*_1H_ + *E*_4L_ (0.84)	*E*_3H_ – *E*_1H_ – *E*_2H_ (0.81)
nn	H_2_O	0.17–7.2	*E*_3H_ – *E*_1H_ – *E*_2H_ (0.81)	Δ*G*_1_ + 2*E*_1H_ (0.80)
all	H_2_O	2.7	*E*_4H_ – *E*_4L_ *– E*_1H_ (0.79)	Δ*G*_3_/(*E*_4H_ – *E*_4L_) (0.76)
all	H_2_O	0.17–7.2	*E*_3H_ – *E*_1H_ – *E*_2H_ (0.71)	*E*_4H_ – *E*_1H_ – *E*_2H_ (0.67)
all	TFE	0.5–3.1	Δ*G*_1_ – *E*_1L_ (0.96)	Δ*G*_2_ + *E*_1L_ (0.96)

a Ligand (3) and Ligand (32) are removed as outliers. Up to three features are allowed per model. Parentheses after the models indicate the Pearson correlation coefficient of that model.

Specifically, the models become better generalizable to different ligand types in H_2_O and the Pearson correlation coefficient increases from *r* = 0.50 to *r* = 0.71. This is important because H_2_O is the most common proton donor for Mn–carbonyl complexes and molecular catalysts for the reduction of CO_2_ in general. The effect of adding an additional feature in the SISSO models for TOF_max_ and overpotential was also explored in Tables S4 and S5. The simple two-feature SISSO models are sufficient in explaining trends with TOF_max_ and overpotential. Adding a third feature does not greatly improve the generalizability of these models across different ligand types, so further analysis is not performed. For TOF_0_, the model *E*_3H_ – (*E*_1H_ + *E*_2H_) stands out in its performance across all subgroups of Mn–carbonyl catalysts.

**Figure 4:**
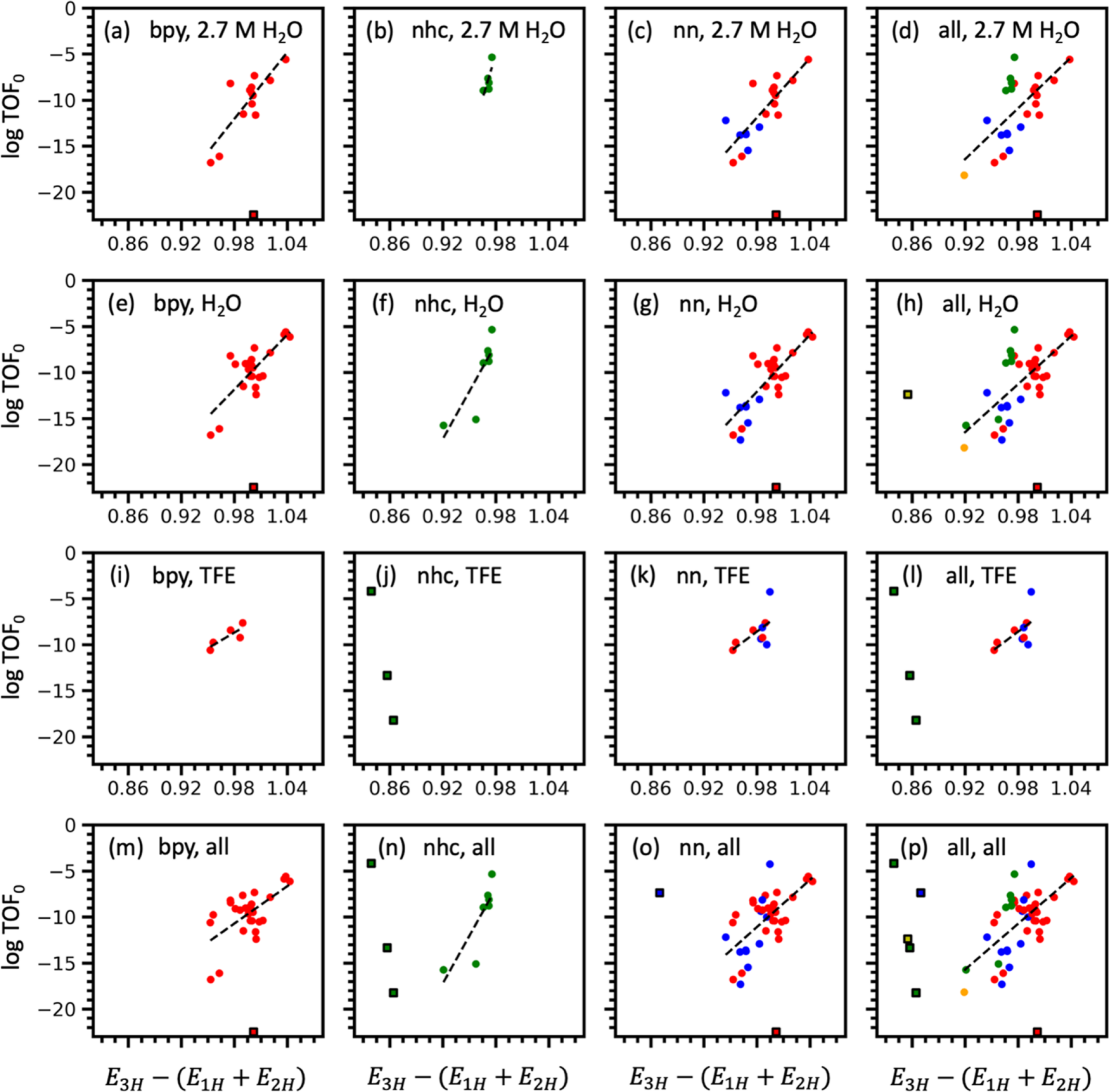
Performance of SISSO model 3 (*E*_3H_ – (*E*_1H_ + *E*_2H_)) for the prediction of TOF_0_ of Mn–carbonyl complexes. Each plot represents different inclusion criteria for ligand type, solvent, and solvent concentration: (a) bpy ligands in 2.7 M H_2_O, (b) NHC ligands in 2.7 M H_2_O, (c) nn ligands in 2.7 M H_2_O, (d) all ligands in 2.7 M H_2_O, (e) bpy ligands in any concentration of H_2_O, (f) all NHC ligands in any concentration of H_2_O, (g) nn ligands in any concentration of H_2_O, (h) all ligands in any concentration of H_2_O, (i) bpy ligands in any concentration of TFE, (k) nn ligands in any concentration of TFE, (l) all ligands in any concentration of TFE, (m) bpy ligands in all solvents types, (n) NHC ligands in all solvent types, (o) nn ligands in all solvent types, (p) all ligands in all solvent types. Outliers are labeled using a square marker with black edges and are excluded from the linear fit shown.

To further refine this model, we performed an analysis of outliers and removed complexes **3**, **32**, **44**–**46**, and **54**. The justification for the removal of these outliers is based on their structures and proton-donor environments, which are substantially different from the rest of the Mn–carbonyl complexes. Complex **3**, ligated by the dihydroxy-substituted bipyridine, was removed because its redox behavior is significantly different from that of all other Mn–carbonyl complexes. Complexes **32** and **44**–**46** were removed because they coordinate to the Mn center via three atoms as opposed to all of the other Mn–carbonyl catalysts, which carry bidentate ligands. Complex **32** is also the only catalyst that uses phenol as the proton donor. Finally, Complex **54** was removed because it has an unusual coordination environment (N–O) and the proton donor concentration is 0.17 M H_2_O, i.e., the smallest in the dataset. To visualize the performance of the model with these outliers removed, we plotted TOF_0_ as a function of the SISSO model *E*_3H_ – (*E*_1H_ + *E*_2H_) for subgroups of ligands, solvents, and solvent concentrations in Figure 4. These models are normalized by the value of *E*_3H_ – (*E*_1H_ + *E*_2H_) for Complex **1** for readability.

TOF_0_ correlates positively with *E*_3H_ – (*E*_1H_ + *E*_2H_) across all ligand types in Figure 4. Higher values of *E*_3H_ – (*E*_1H_ + *E*_2H_) lead to a higher intrinsic activity as measured by the TOF extrapolated to zero overpotential. Ideally, the value *E*_1H_ + *E*_2H_ is minimized such that the HOMO energies *E*_1H_ and *E*_2H_ are very low leading to stable [Mn(κ^2^-L)(CO)_3_H]^0^ and [Mn(κ^2^-L)(CO)_3_CO_2_]^−^ intermediates. This is the same condition as that which was found to minimize the overpotential in Figure 3. At the same time, *E*_3H_ should be maximized such that the HOMO energy *E*_3H_ is high. A high *E*_3H_ value describes an [Mn(κ^2^-L)(CO)_3_]^−^ intermediate that can be oxidized easily. A high HOMO energy for this active complex likely correlates with a higher TOF_0_ because electrons would be more easily transferred from [Mn(κ^2^-L)(CO)_3_]^−^ to CO_2_ to weaken the C–O bond. Cleavage of the C–O bond, assisted by the proton donor, should thus occur more readily.

Remarkably, these correlations hold across multiple different ligand types and solvent environments. The Pearson correlation coefficients for bpy ligands in H_2_O are *r* = 0.83 and *r* = 0.77 for Figure 4a,e, respectively. For nn ligands in H_2_O, *r* = 0.81 for Figure 4c,g. For NHCs, *r* = 0.77 and *r* = 0.84 for Figure 4b,f, respectively. These values are near the limits for accuracy considering possible measurement errors from experiments or FOTW analysis. Even when all ligand types are considered together in H_2_O, the trend holds well with *r* = 0.71 for Figure 4d. Positive correlations are also seen for Mn–carbonyl catalysts using TFE as the proton donor, albeit the lack of data makes it hard to draw reliable conclusions. Nevertheless, it can be confidently concluded that *E*_3H_ – (*E*_1H_ + *E*_2H_) is a good model for predicting TOF_0_ across a series of Mn–carbonyl complexes with bidentate ligands in similar electrochemical environments.

## Conclusions

Mn–carbonyl complexes present attractive molecular electrocatalysts for the reduction of CO_2_ to CO. To date, dozens of Mn–carbonyl complexes with different noninnocent ligands have been investigated with respect to their ability to catalyze the reduction of CO_2_ at low overpotentials and high turnover frequencies (TOFs). A thorough understanding of what ligand properties can lead to Mn–carbonyl catalysts with good performance has been elusive, thereby limiting progress in the systematic design of molecular electrocatalysts. To address this problem, the electronic features of 55 Mn–carbonyl complexes were calculated and correlated to experimental figures of merit to establish physical models for catalyst activity. The dataset of 55 Mn–carbonyl catalysts consists of catalysts with different ligand structures that have previously been examined experimentally. The cyclic voltammograms of these catalysts were manually extracted from the literature and analyzed using the foot-of-the-wave (FOTW) method to give information on half-wave potentials and turnover frequencies (TOFs). These activity metrics were derived from cyclic voltammograms in a consistent manner, which allows for a direct comparison of the catalysts.

The electronic features of the relevant catalytic intermediates for each of the 55 Mn–carbonyl complexes were examined using density functional theory (DFT) calculations. These four intermediates were selected based on their commonality in the currently accepted catalytic mechanisms for the electrocatalytic reduction of CO_2_. Energies of the HOMO/LUMO orbitals, partial charges, and Gibbs free energies for key mechanistic steps were calculated as the primary features for the electronic structure of the Mn–carbonyl catalysts. Using the SISSO method, combinations of primary features with mathematical operators were constructed and screened based on correlation with the experimentally derived activity metrics. These mathematical models allowed for easy data-analytical interpretation, which provided insights into what ligand features make for an active Mn–carbonyl catalyst for the electrocatalytic reduction of CO_2_. The following models were found to accurately predict changes in TOF_max_ (eq 3), overpotential (eq 4), and TOF_0_ (eq 5) across a series of Mn–carbonyl electrocatalysts.
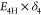
3
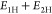
4
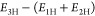
5The features that constitute these models expose relationships between ligand features and electrocatalytic activity. TOF_max_ for Mn–carbonyl catalysts is highly correlated with the ligand properties of the [Mn(κ^2^-L)(CO)_4_]^0^ intermediate, but optimal ligand criteria depend on the ligand type (eq 3). The reduction of [Mn(κ^2^-L)(CO)_4_]^0^ to reform the active complex may limit the TOF_max_ for bipyridine derivatives, whereas the opposite is true for *N*-heterocyclic carbenes. The overpotential is minimized with low-energy HOMOs for the [Mn(κ^2^-L)(CO)_3_H]^0^ and [Mn(κ^2^-L)(CO)_3_CO_2_]^−^ intermediates (eq 4). This reflects an ease of binding CO_2_ and H^+^ to the Mn center, triggering the reduction of CO_2_. An ideal catalyst has both a high TOF_max_ and a low overpotential, which are both considered with the TOF_0_ figure of merit. TOF_0_ can be maximized with the same criteria as for low overpotential, with the inclusion of a high-energy HOMO for the active [Mn(κ^2^-L)(CO)_3_]^−^ complex (eq 5). The less stable [Mn(κ^2^-L)(CO)_3_]^−^ can more easily transfer electrons to CO_2_ to break the C–O bond with the assistance of the proton donor.

Prior computational studies of Mn–carbonyl catalysts have focused primarily on structure and mechanism prediction for no more than a handful of different ligand types at a time. In contrast, this work provides calculated electronic features of mechanistically relevant intermediates for a relatively large group of molecular electrocatalysts, and these features were correlated to experimentally derived activity metrics. These models can be expected to provide valuable information for the rational design of electrocatalysts by identifying important catalytic intermediates and their features that correlate with improved activity. The dataset itself (Table S6) can be built upon and used for other tasks as new Mn–carbonyl catalysts are characterized.

Future applications of this work will focus on using these models to design ligand types with favorable properties that maximize TOF and minimize overpotential. The experimental synthesis and characterization of such catalysts may subsequently be carried out to validate the descriptors identified in this work. To improve the predictive accuracy of the SISSO models, additional primary features can be introduced to give information on the geometry of the ligand. This workflow could also be extended to molecular electrocatalysts with other transition-metal centers (e.g., Fe, W, Re, or Cr), molecular photocatalysts, and homogeneous catalysts for other reaction types (i.e., H_2_ reduction or O_2_ reduction).

## Computational Methods

All DFT calculations were performed using NWChem computational chemistry software version 6.8.^51^ For geometry optimizations and vibrational frequencies, the B3LYP functional was used with a D3 dispersion correction.^52,53^ To model the Mn–carbonyl complexes, a mixed basis set was used with the all-electron Def2-TZVPD basis set for Mn and the Def2-SVPD basis set for C, O, N, H, P, F, and S.^54^ The B3LYP functional was chosen for its ability to accurately quantify ligand-dissociation energies and bond energies for 3d-transition-metal carbonyl complexes.^55,56^ The addition of diffuse basis functions is important to correctly describe long-range electron interactions in negatively charged ions, as is the case for some of the Mn–carbonyl complexes considered here.^57^ Implicit solvation was included using the COSMO model with a dielectric constant of 35.6 corresponding to acetonitrile.^58,59^ Electronic energies of molecules were corrected for enthalpy and entropy contributions at 298 K based on a calculation of harmonic vibrational frequencies. The Gibbs free energies of the Mn–carbonyl complexes were calculated and used to derive mechanistic descriptors, i.e., the binding energy of CO_2_ to the active complex (Δ*G*_CO_2__), the p*K*_a_ of the active complex (p*K*_a_), and the potential where CO dissociates to reform the active complex (*U*_CO_).

## Supporting Information Available

Details of determining overpotential from cyclic voltammograms; derivation of foot-of-the-wave (FOTW) method for calculating TOF_max_ from experimental cyclic voltammograms; relative uncertainty in TOF_max_ values; primary feature correlation maps; supplemental SISSO models with added features and outliers removed; and full dataset of 55 Mn–carbonyl complexes (PDF)
