# Supplementary material for: Analyzing Structure–Activity Variations for Mn–Carbonyl Complexes in the Reduction of CO2 to CO
Source: Inorg Chem. 2022 Dec 21;62(1):318–35. doi: 10.1021/acs.inorgchem.2c03391 (PMC9832541; doi:10.1021/acs.inorgchem.2c03391)
Supplement: Supplementary file 1 — ic2c03391_si_001.pdf [file ic2c03391_si_001.pdf]

Supporting Information

Analyzing Structure-Activity Variations for Mn-Carbonyl Complexes in the Reduction of CO_2 to CO.

Jacob Florian[a] *and Jacqueline M. Cole** [a,b]

[a] Cavendish Laboratory, University of Cambridge, J.J. Thomson Avenue, Cambridge, CB3 0HE, UK.

[b] ISIS Neutron and Muon Source, STFC Rutherford Appleton Laboratory, Harwell Campus for Science and Innovation, Didcot, OX11 0QX, UK.

*Email: jmc61@cam.ac.uk (J. M. Cole)

Table of Contents

***Overpotential from Experimental Cyclic Voltammograms* 2**

***Foot of the Wave (FOTW) Analysis for Calculating TOF_{max} from Experimental Cyclic Voltammograms* 3**

***Relative Uncertainty from FOTW Analysis* 9**

***Comparing Reported and Calculated TOF_{max} Values* 10**

***Feature Correlations* 12**

***List of SISSO Models* 16**

***Full Dataset of 55 Mn-Carbonyl Catalysts* 19**

***FOTW Analysis Plots for the Mn-Carbonyl Catalysts* 26**

***References* 53**

Overpotential from Experimental Cyclic Voltammograms

The $E_{cat/2}$ values for each catalytic wave (**Figure S1a**) were determined based on the local maxima in the first derivative plots in **Figure S1b**. This is a method similar to the one used previously to evaluate half-wave potentials for molecular cobalt complexes for H_2 evolution.[1] When multiple local maxima are present, as is the case in **Figure S1**, the maximum associated with greater catalytic enhancement (usually the reduction-first pathway) is chosen as the $E_{cat/2}$ of the catalyst. While there may be some ambiguity as to how accurately $E_{cat/2}$ represents the true half-wave potential of the catalyst, it is a consistent method for finding $E_{cat/2}$ for the purpose of evaluating previously reported catalysts. Furthermore, it has been demonstrated that $E_{cat/2}$ values estimated from non-catalytic waves show only a small variance from the true $E_{cat/2}$ values.[2]

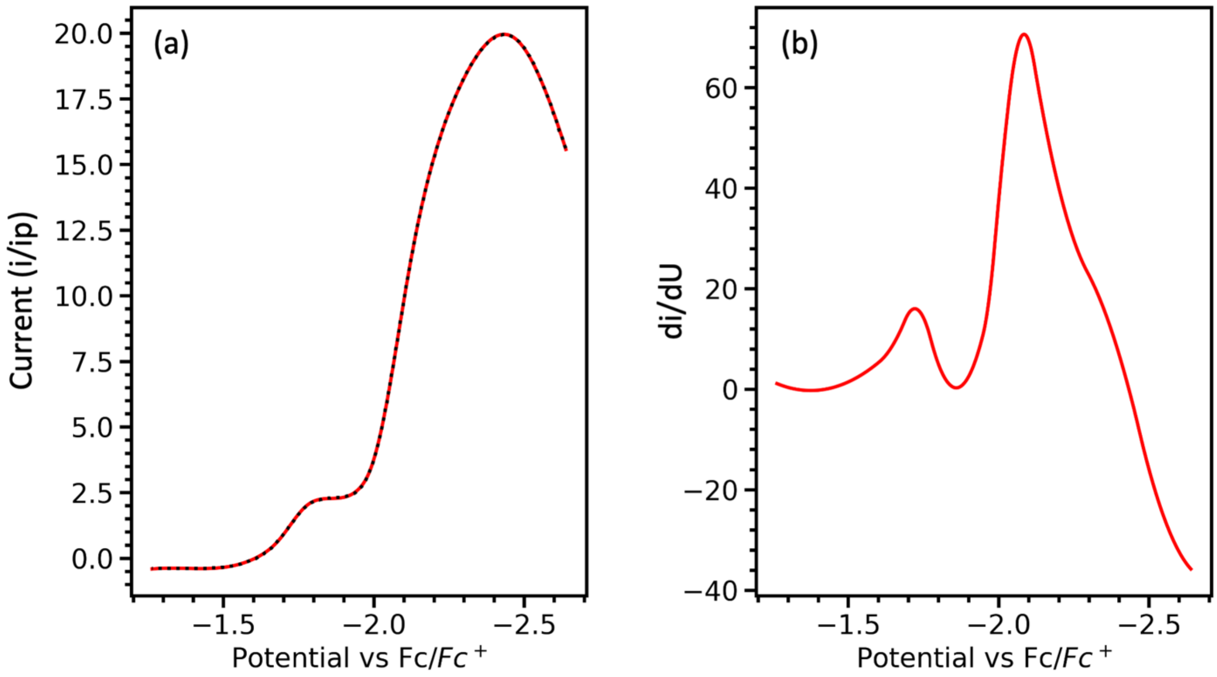

Figure S1. (a) Forward trace of the cyclic voltammogram of [Mn(bpy)$(CO)_3$Br] under CO_2 and 2 M trifluoroethanol.[3] The black dotted line represents the individual data points mined from ref. 11, while the red line represents a spline fitting. (b) First derivative of the current, i.e., the red line in (a). The peaks represent inflection points and half-wave potentials for the two catalytic waves in (a). The half-wave potential for this catalyst is -2.08 V vs Fc/Fc^+.

The thermodynamic potential for the reduction of CO_2, $E^0_{CO_2/CO}$, is also required to evaluate overpotential using **Equation 1**. To determine $E^0_{CO_2/CO}$ vs SHE in acetonitrile, **Equation S1** was used, which has been developed by following a thermodynamic cycle[4,5] and by adding a conversion factor of -0.63 V to convert from SHE to Fc/Fc$^+$.[6]

$$E^0_{CO_2/CO} = 0.287 - \left(\frac{2.303RT}{F}\right)pK_a - 0.63 \quad \text{(S1)}$$

The pKa values of water, phenol, and trifluoroethanol in acetonitrile are 31.4 (in DMSO),[7] 29.2,[8] and 35.4,[9] respectively. The pKa value of CO_2 in acetonitrile is 17.03,[4] which is the strongest acid present in all cases. Therefore, $E^0_{CO_2/CO}$ = -1.35 V vs Fc/Fc$^+$ was chosen for all Mn-carbonyl catalysts considered in the present study.

Foot of the Wave (FOTW) Analysis for Calculating TOF_{max} from Experimental Cyclic Voltammograms

Calculating the TOF_{max} for Mn-carbonyl catalysts from different kinetic studies is not trivial. In some cases, catalyst TOF_{max}, TOFs at certain potentials, or catalytic-current enhancements have already been reported, albeit that often differences in how these figures are calculated make it challenging to use them for accurate comparisons. Furthermore, in a majority of the 55 Mn-carbonyl catalysts considered here, no kinetic figures are reported, and only the cyclic voltammogram itself is given as a measure of catalyst performance. Therefore, only the shape of the cyclic voltammogram can be used to calculate the TOF_{max}.

To get an understanding of how the shape of a cyclic voltammogram can yield kinetic information about a catalyst, we derive the expression for the catalytic current following a simplified reaction scheme. The reduction of CO_2 to CO by Mn-carbonyl catalysts can be described generally by **Equations S2-S4**. It should be noted here that several electron transfers and chemical steps are omitted for simplicity, and the full mechanism is described in more detail in **Figure 1**.

$$[MnCO]^+ + 2e^- \rightleftarrows [Mn]^- + CO \quad \text{(S2)}$$

$$[Mn]^- + CO_2 \rightarrow [MnCO_2]^- \quad \text{(S3)}$$

$$[MnCO_2]^- + 2H^+ \rightarrow [MnCO]^+ + H_2O \quad \text{(S4)}$$

For clarity, $[MnCO]^+$will be abbreviated to P, while $[Mn]^-$ will be abbreviated to Q in order to match **Scheme 1**. A key approximation made here is that the rate of electron transfer between P/Q at the

electrode is fast, such that the Nernst equation can be used. Using the steady-state approximation for the $[Mn - CO_2]^-$ intermediate, the rate of production of the products (CO and H_2O) can be expressed using a general rate constant for catalysis, k_{cat}, given in **Equations S5-S6**.[10]

$$\frac{dC_{CO}}{dt} = k_1 C_{CO_2} C_Q \tag{S5}$$

$$k_{cat} = k_1 C_{CO_2} \tag{S6}$$

Then, k_{cat} can be considered as a pseudo-first-order rate constant, because the concentration of CO_2 far exceeds the concentration of the catalyst in all cases and can be considered constant. The concentration of CO_2 is kept constant at the saturation point, which is 0.28 M CO_2 in acetonitrile.[11] The Nernst equation is given by **Equation S7**, and it can be rearranged to give an expression for the concentration of Q at the electrode surface in **Equation S8**.

$$E = E^0 + \frac{RT}{F} \ln\left(\frac{C_{Q,0}}{C_{P,0}}\right) \tag{S7}$$

$$C_{Q,0} = \frac{C_P^0}{1 + \exp\left[\frac{F}{RT}(E - E^0)\right]} \tag{S8}$$

The total concentration of catalyst C_P^0 remains constant, such that $C_P^0 = C_Q + C_P$ (**Equation S8**). During the catalytic reaction, Q is only found between the surface (x = 0) in the reaction-diffusion layer (0 < x < μ), which has a thickness μ.[12] In the reaction-diffusion layer, the concentration profile of Q can be expressed by the differential equation in **Equation S9** assuming all mass transport is driven by diffusion. The concentration profile of Q is affected by diffusion of Q from the electrode surface, where P gains electrons to form Q, and by consumption of Q by reaction with molecules of CO_2 that are near the surface.

$$D\frac{d^2C_Q}{dx^2} - k_{cat}C_Q = 0 \tag{S9}$$

The boundary conditions $C_Q(x = \mu)$ = 0 and $\left(\frac{dC_Q}{dx}\right)_{x=\mu}$ = 0 apply to **Equation S9**, such that the concentration of Q decays to zero at μ and remains zero after the edge of the reaction-diffusion layer. **Equation S10** gives the general solution using these boundary conditions.

$$C_Q(x) = C_{Q,0}\, exp\left(-\sqrt{\frac{k_{cat}}{D}}x \right) \quad \text{(S10)}$$

Now that the concentration profile of Q is known, the current flowing through the electrode surface can be related to the concentration gradient of Q. **Equation S11** expresses the current density at the electrode surface (x = 0) in terms of the fluxes of P and Q.[10] By substituting **Equation S10** for $C_Q(x)$, **Equation S12** is obtained.

$$\frac{i}{A} = nFD\left(\frac{dC_P}{dx}\right)_{x=0} = -nFD\left(\frac{dC_Q}{dx}\right)_{x=0} \quad \text{(S11)}$$

$$\frac{i}{A} = C_{Q,0}nF\sqrt{k_{cat}D} \quad \text{(S12)}$$

In **Equation S12**, A is the electrode surface area, F is the Faraday constant, and D is the diffusion coefficient of P and Q. Using the Nernst equation to express the concentration of Q as a function of potential (**Equation S8**), the current can be expressed instead as a function of total catalyst concentration, C_P^0 (**Equation S13**).

$$i = \frac{nFAC_P^0\sqrt{k_{cat}D}}{1 + exp\left[\frac{F}{RT}(E - E^0)\right]} \quad \text{(S13)}$$

Adhering to the assumptions described above, **Equation S13** should exactly define the current-potential relationship of the experimental cyclic voltammograms under purely kinetic conditions. This gives rise to a sigmoid shape, where at sufficiently positive potentials $i = 0$, and at sufficiently negative potentials $i = i_{plateau}$. To evaluate kinetic parameters and gain mechanistic insight of Mn-carbonyl catalysts, catalytic-plateau-current analysis is most often used in practice, because diffusion coefficients and electrochemically active surface areas are unknown. The catalytic plateau current is that current, where at sufficiently negative potentials all catalyst molecules are in the active state; it is also the plateau of the sigmoid response. For an electrochemical reduction followed by an irreversible chemical step (EC' mechanism), the plateau of the catalytic current is given by **Equation S14**.[13]

$$i_{plateau} = nFAC_P^0\sqrt{D}\sqrt{TOF_{max}} \quad \text{(S14)}$$

In this equation, F is the Faraday constant, A is the electrochemically active surface area, D is the diffusion coefficient of catalyst P, C_P^0 is the total concentration of catalyst P in solution, and TOF_{max} is the

maximum turnover frequency, i.e., the observed rate constant. In practice, $i_{plateau}$ can be easily determined from the plateau current of an ideal S-shaped CV curve.

The plateau current is often normalized by the current under inert conditions. Under inert conditions, the diffusion coefficients and electrochemically active surface area should be the same as when the substrate (here: CO_2) is introduced. Dividing $i_{plateau}$ by the peak current in the absence of substrate, i_p, is useful because it enables the evaluation of TOF_{max} without needing to know the electrochemically active surface area and diffusion coefficients.[13] **Equation S15** gives an expression for i_p assuming that the electron transfer between P and Q is Nernstian, while **Equation S16** gives an expression for the normalized plateau current.

$$i_p = 0.4463zFAC_P^0\sqrt{\frac{zFvD}{RT}} \tag{S15}$$

$$\frac{i_{plateau}}{i_p} = \frac{2.24n}{z^{3/2}}\sqrt{\frac{RT}{Fv}}\sqrt{TOF_{max}} \tag{S16}$$

In these equations, v is the scan rate that measures how fast the voltage is swept up and down in the cyclic voltammogram. While n describes the number of electrons needed to convert one molecule of substrate into one molecule of product in the catalytic reaction, z describes the number of electrons transferred per unit catalyst in the non-catalytic response.

In practice, the TOF_{max} of a catalyst can be calculated with just two measurements: (1) The plateau current from the catalytic cyclic voltammogram, and (2) the peak current in the non-catalytic cyclic voltammogram. However, plateau-current analysis is only applicable to S-shaped CV curves where the reverse scan exactly overlaps with the forward scan. This means that catalysis is purely limited by kinetics and there is mass-transport limits caused by substrate consumption.[14] The plateau current must also be independent of the scan rate for the TOF_{max} calculated from this method to be valid. These strict conditions are rarely met for molecular catalysts. Often, apparent rate constants and TOFs will be reported from imperfectly shaped cyclic voltammograms, or the kinetics are discussed only qualitatively using the ratio of peak current in the presence of substrate to the peak current in the absence of substrate.

The presence of side reactions and substrate depletion distort the shape of the cyclic voltammogram, making it harder to estimate kinetic parameters. FOTW analysis is a technique that allows determining the TOF_{max} of a molecular catalyst from cyclic-voltammogram curves without requiring a kinetically limiting regime with a well-defined plateau current. This makes it particularly useful for the

purpose of consistently evaluating activity metrics across multiple Mn-based molecular catalysts for the reduction of CO_2. The FOTW method has been described in detail for two-electron-transfer processes,[5,15–18] and has been previously used to determine rate constants for the electrochemical reduction of CO_2 promoted by molecular Mn-carbonyl catalysts.[19–21]

At the "foot" of the catalytic cyclic voltammogram, i.e., at low overpotentials, substrate depletion and other side phenomena are less likely to occur. Using data near the foot of the wave and extrapolating **Equation S13** to higher potentials allows for a more accurate determination of TOF_{max}. More importantly, this allows for consistent method of calculating TOF_{max} for catalysts that have been reported in the literature without relying on individualized kinetic analysis that may be absent.

To evaluate TOF_{max} for all 55 Mn-carbonyl catalysts considered here, cyclic voltammograms were mined from individual papers and normalized according to peak current. Two cyclic voltammograms were captured for each catalyst from the publication using DataThief.[22] DataThief is a program that generates data points from images of graphs, which can then be re-plotted and used for further analysis. One cyclic voltammogram shows the current as a function of potential under catalytic conditions, i.e., in the presence of CO_2 and a proton donor. The second cyclic voltammogram shows the redox behavior of the catalyst under an inert atmosphere (Argon or N_2), and whose peak current is used to normalize the catalytic current. Both cyclic voltammograms should be performed using the same scan rate and the same concentration of catalyst. The catalytic current is then normalized by the peak current, and the expected functional form (for a kinetically limiting regime) is given in **Equation S17**.

$$\frac{i}{i_p} = \frac{\frac{2.24n}{z^{3/2}}\sqrt{\frac{RT}{Fv}}\sqrt{TOF_{max}}}{1 + \exp\left(\frac{F}{RT}\left(E - E_{cat/2}\right)\right)} \tag{S17}$$

Here, z is the number of electrons transferred per catalyst in the non-catalytic response (1 or 2), while n is the number of electrons required for catalytic turnover (2), and i_p is the peak of the non-catalytic reduction wave under inert atmosphere.

The ideal S-shaped curve has the functional form $\left(1 + \exp\left(\frac{F}{RT}\left(E - E_{cat/2}\right)\right)\right)^{-1}$. When the normalized catalytic current $\left(\frac{i}{i_p}\right)$ is plotted as a function of the sigmoid response curve $\Big(1 +$

$\exp\left(\frac{F}{RT}\left(E - E_{cat/2}\right)\right)\Big)^{-1}$, the slope (m) of the corresponding line can be used to determine the maximum turnover frequency, which is given by **Equation S18**.

$$TOF_{max} = \left(\frac{m}{2.24}\right)^2 \left(\frac{Fv}{RT}\right)\left(\frac{z^3}{n^2}\right) \tag{S18}$$

Deviations from the ideal behavior will appear as nonlinearities far from the FOTW, when $|E| \gg |E_{cat/2}|$. Side-phenomena will be minimized at potentials near $E_{cat/2}$, and the slope near $E_{cat/2}$ will give information about the kinetics.

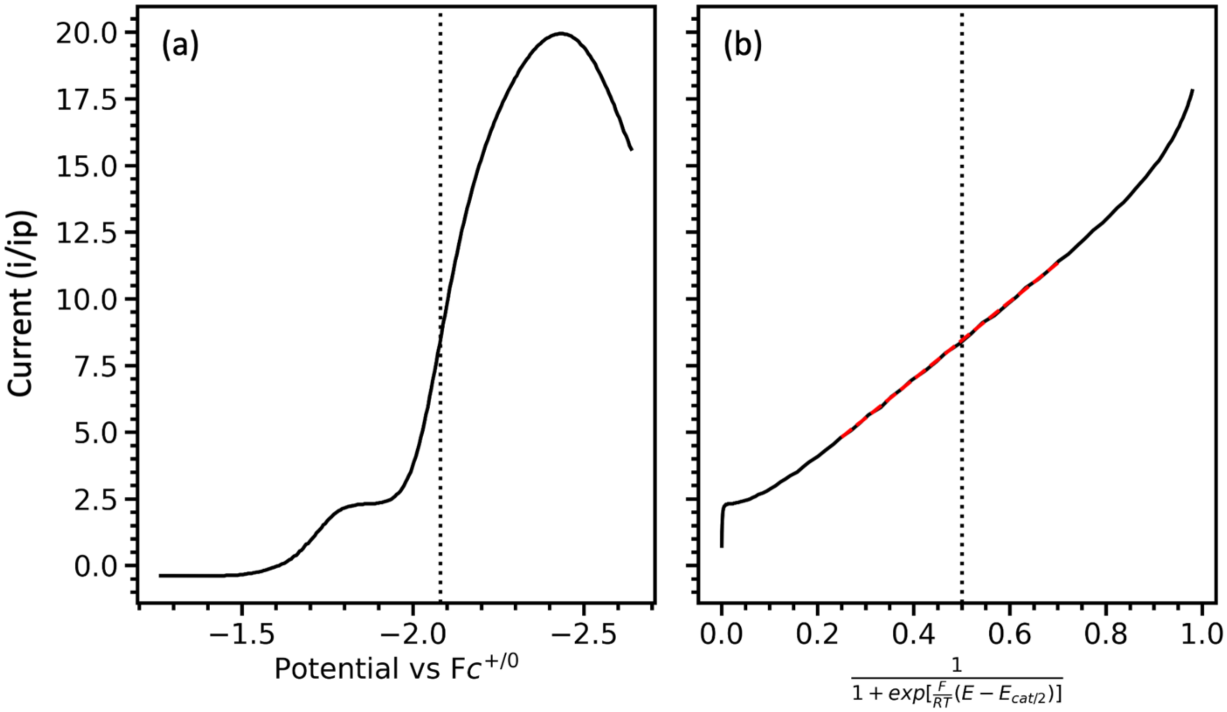

Figure S2. (a) Forward trace of the cyclic voltammogram of [Mn(bpy)(CO)$_3$Br] under CO_2 and 2 M trifluoroethanol[3] plotted as a function of (a) potential and (b) the sigmoid function centered at $E_{cat/2}$. The dotted vertical lines in each plot represent the location of $E_{cat/2}$. The dashed red line in (b) is a FOTW linear fit near $E_{cat/2}$, where the impact of side phenomena is not significant.

Figure S2 represents the extension of **Figure S1** to calculate TOF_{max} using FOTW analysis. The catalytic half-wave potential $E_{cat/2}$ = -2.08 V corresponds to a value of 0.5 in **Figure S2b**. Near the half-

wave potential, the FOTW plot is linear. At very low potentials, there are deviations from nonlinearity that arise from an overlap of another wave at lower overpotentials. At high potentials, there are deviations from linearity as substrate depletion results in a peak of the current. The slope of the red dashed line in **Figure S2b** is calculated using a linear fit from x = 0.25 to x = 0.70 and **Equation S18** is used to calculate TOF_{max}.

Relative Uncertainty from FOTW Analysis

The relative uncertainty in TOF_{max} between different Mn-carbonyl catalysts was calculated to assess the confidence of values calculated using FOTW analysis. To assess the uncertainty in the TOF_{max} determined from the FOTW analysis, the difference between the slope in the strictly linear region and the slope at low values of (i/ip) in **Figure S3b** is examined. If the slope at low values of (i/ip) is higher (lower) than in the strictly linear region, the catalysis is faster (slower) at the FOTW than expected. In these cases, it is likely that the wave shape in **Figure S3a** is a result of a superposition of several waves, which may correspond to current from different mechanisms or side reactions with different onset potentials. FOTW analysis assumes that peaks corresponding to redox events are well-separated, but this requirement is not strictly met in all cases. The peak overlap leads to an uncertainty in the TOF_{max} extracted from the linear region using FOTW analysis.

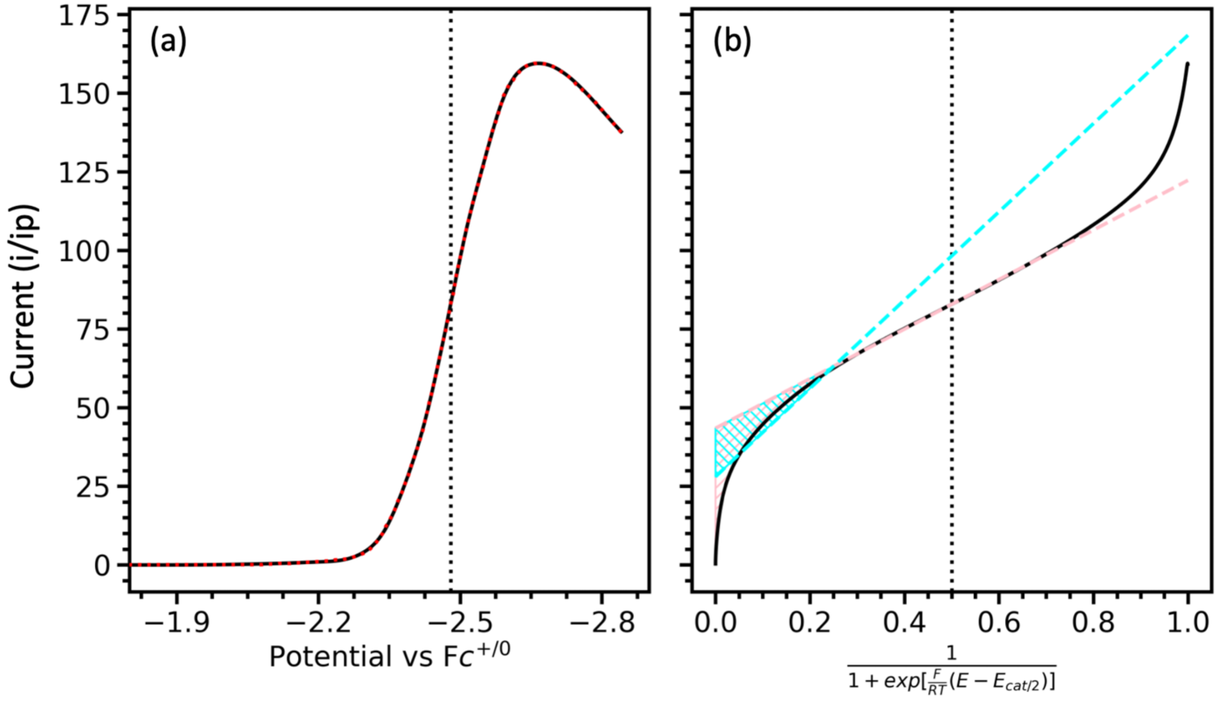

Figure S3. Assessing the relative uncertainty for TOF_{max} values by area deviation near the FOTW. (a) Forward trace of the cyclic voltammogram of complex **52** under CO_2 and 0.56 M H_2O.[23] The black dotted

line represents the individual data points mined from ref. 27 and the red line represents a spline fitting. (b) FOTW plot showing deviations from linearity near the FOTW.

To quantify the impact of these side phenomena in the cyclic voltammograms, the magnitude of the area above or below the ideal linear FOTW region is measured in the region x = 0 to x = 0.25 (pink shaded region between the forward trace of cyclic voltammogram (black line) and the pink dashed in **Figure S3b**). The greater this area is, the more the FOTW region is overshadowed by additional redox events that are not related to the catalytic wave at $E_{cat/2}$. To give meaning to this area, a line of slope m is constructed that intersects the FOTW pink dashed line at x = 0.25, and that has the same magnitude of area in the pink shaded region (cyan dashed line and cyan shaded in **Figure S3b**). Finally, **Equation S18** is used to calculate a revised TOF_{max} using the slope of the new cyan dashed line (m). The difference between the revised TOF_{max} and the TOF_{max} calculated using the linear region (pink line) gives a relative uncertainty in the TOF_{max} for a given Mn-carbonyl catalyst.

Comparing Reported and Calculated TOF_{max} Values

It is important to validate the accuracy of the FOTW analysis in determining accurate values of TOF_{max} from cyclic voltammograms. For cases where TOF_{max} is reported, it is usually calculated via plateau current analysis using **Equation S16**. However, this is the case for only 22 out of the 55 Mn-carbonyl catalysts in our dataset. A plot showing the calculated TOF_{max} values as a function of the reported TOF_{max} values for these 22 catalysts is given in **Figure S4**.

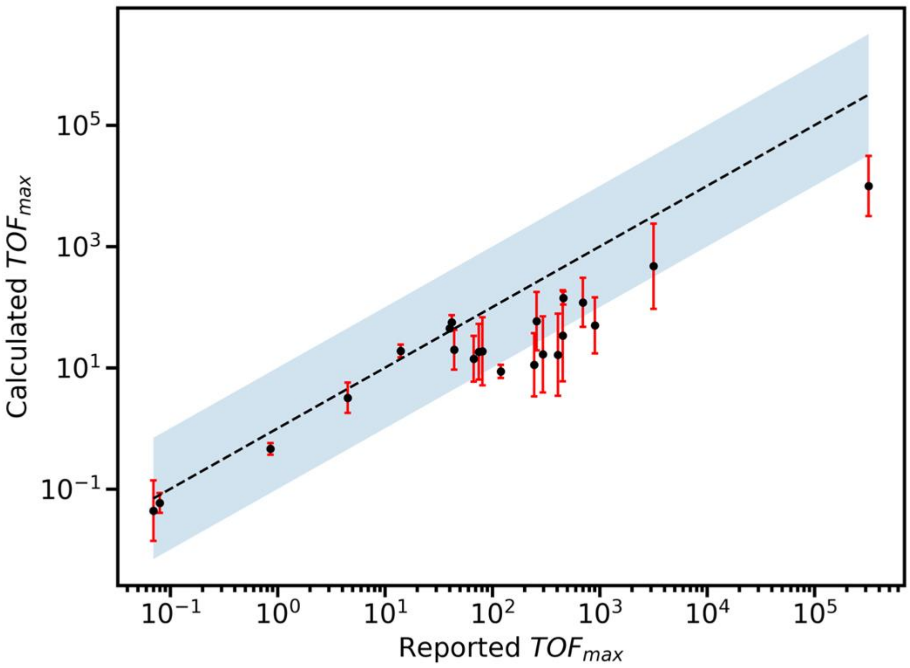

Figure S4. TOF_{max} values calculated using FOTW analysis plotted as a function of reported TOF_{max} values (where available). The black dotted line represents an equal value for calculated and reported TOF_{max}, values, and the blue shaded area represents deviations within one order of magnitude. The red error bars are a measure of relative uncertainty in the calculated TOF_{max} values.

The calculated TOF_{max} values using FOTW analysis appear to be a good approximation for the actual TOF_{max} values of the catalysts at low TOFs. At higher TOFs, FOTW analysis appears to systematically underestimate the TOF_{max} for the catalyst. This is a drawback of the present implementation of the FOTW analysis because the electron-transfer kinetics between the electrode and the catalyst are not considered. This becomes increasingly important with active catalysts that display high TOF_{max} values. Electron transfer between the Mn-carbonyl catalyst and the substrate (e.g., CO_2) becomes so fast that electron transfer from the electrode to the catalyst limits the overall kinetics.[17] The FOTW can be extended to these cases by including an additional term that uses the rate constant of electron transfer between the catalyst and electrode (k_s in **Scheme 1**), which can be derived from the cyclic voltammogram of the catalyst redox behavior under inert conditions; however, these electrode-catalyst rate constants are not reported for any of the Mn-carbonyl catalysts considered here. Instead, the current implementation assumes that electron transfer between electrode and catalyst is infinitely fast, and it should be noted here that this may lead to an underestimation of TOF_{max} for very active catalysts.

The inclusion of a "fudge factor" was considered by fitting a curve to **Figure S4** to correct this underestimation. As k_s depends on the properties of the catalyst and electrode material, such a universal

correction may capture incorrect behavior when extrapolated to other catalysts that are not in **Figure S4**. In either case, there is a clear positive correlation between TOF_{max} calculated using FOTW analysis and the reported TOF_{max}, which is adequate for comparing the underlying trends across a series of these catalysts.

Feature Correlations

Some of the features in **Table 2** are strongly correlated to each other. Relationships between each of the features are illustrated using a heatmap of the pairwise-correlation coefficients in **Figure S5**. The intensity of the color and the size of the squares in **Figure S5** quantify the degree to which two features are correlated. ΔG_1 and ΔG_2 are negatively correlated with a Pearson correlation coefficient of -0.75. Generally, a highly negative adsorption energy for CO_2, i.e., a strong Mn-CO_2 bond, also gives rise to a highly positive energy for dissociation energy for H^+ with the same Mn-complex (i.e., strong Mn-H bond and high pKa). This negative correlation describes the adsorbate scaling relation for Manganese with H and C atoms. Ligand modifications that are designed to stabilize the Mn-CO_2 reaction intermediate via intramolecular hydrogen bonding can break this correlation. Another interesting correlation that is present in **Figure S5** is between E_{1L} and E_{2L}. These two are almost perfectly correlated (r = 0.99), which means that the LUMO orbitals of the H-bound and CO_2-bound Mn intermediates are influenced the same way by any changes in the ligand structure. The energy of the LUMOs of the complexes correlate to the partial charge for a given complex. In general, the LUMOs tend to correlate strongly with each other whereas the HOMOs don't.

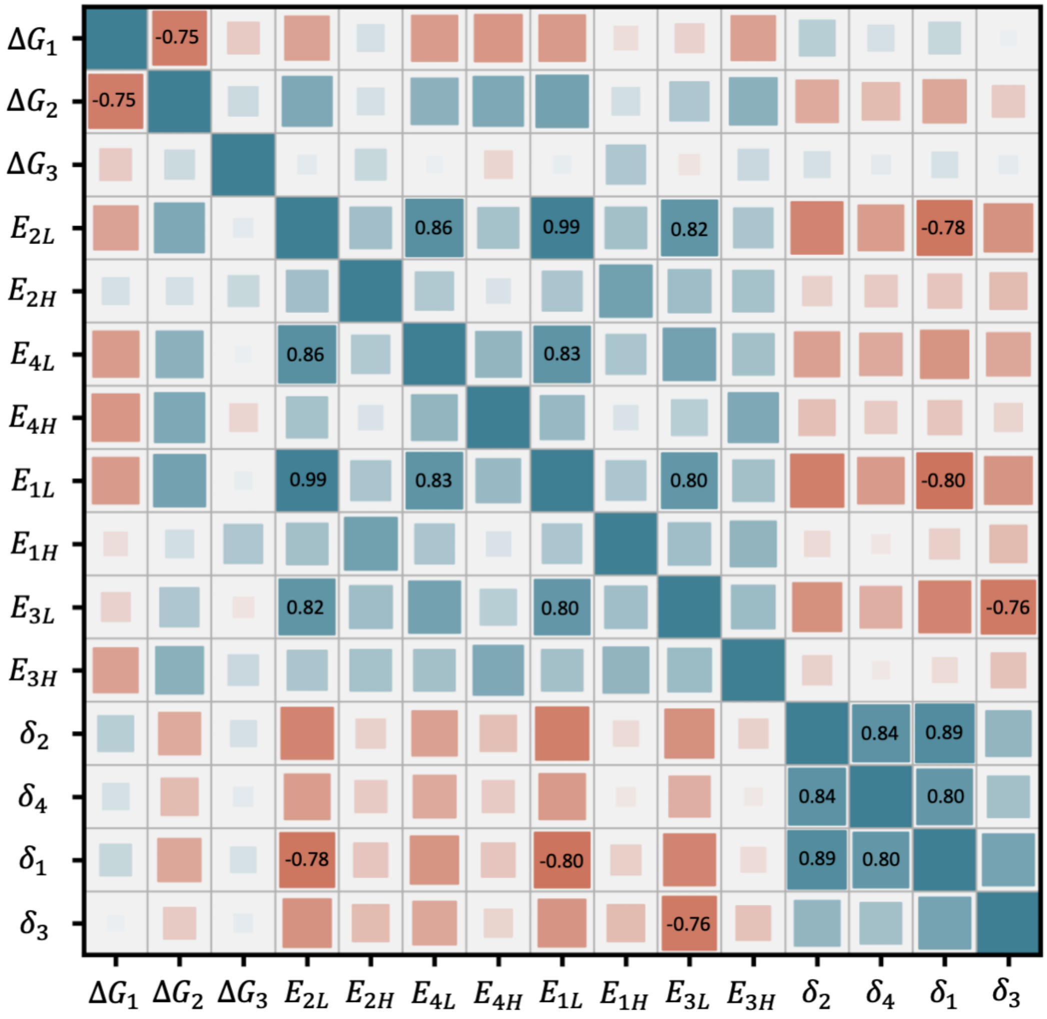

Figure S5. Heatmap showing pairwise correlations between features in Table 2. The Pearson correlation coefficient is reported for each pairwise correlation if it is greater than 0.75. Red represents a negative correlation, while blue represents a positive correlation. The intensity of the color and the size of the squares quantify how strongly the two features are correlated.

Figures S6-S8 show correlations between the individual primary features in **Table 2** and experimental figures of merit (TOF_{max}, overpotential, and TOF_0, respectively). For simplicity, only Mn-carbonyl catalysts with cyclic voltammograms reported in H_2O are shown. For each figure, the color of the point gives the ligand type of the Mn-carbonyl catalyst such that red = bpy, blue = nn (ex. bipyridine), green = NHC, orange = P-N ligands, and yellow = O-N ligands. This is the same color scheme used throughout the rest of the paper.

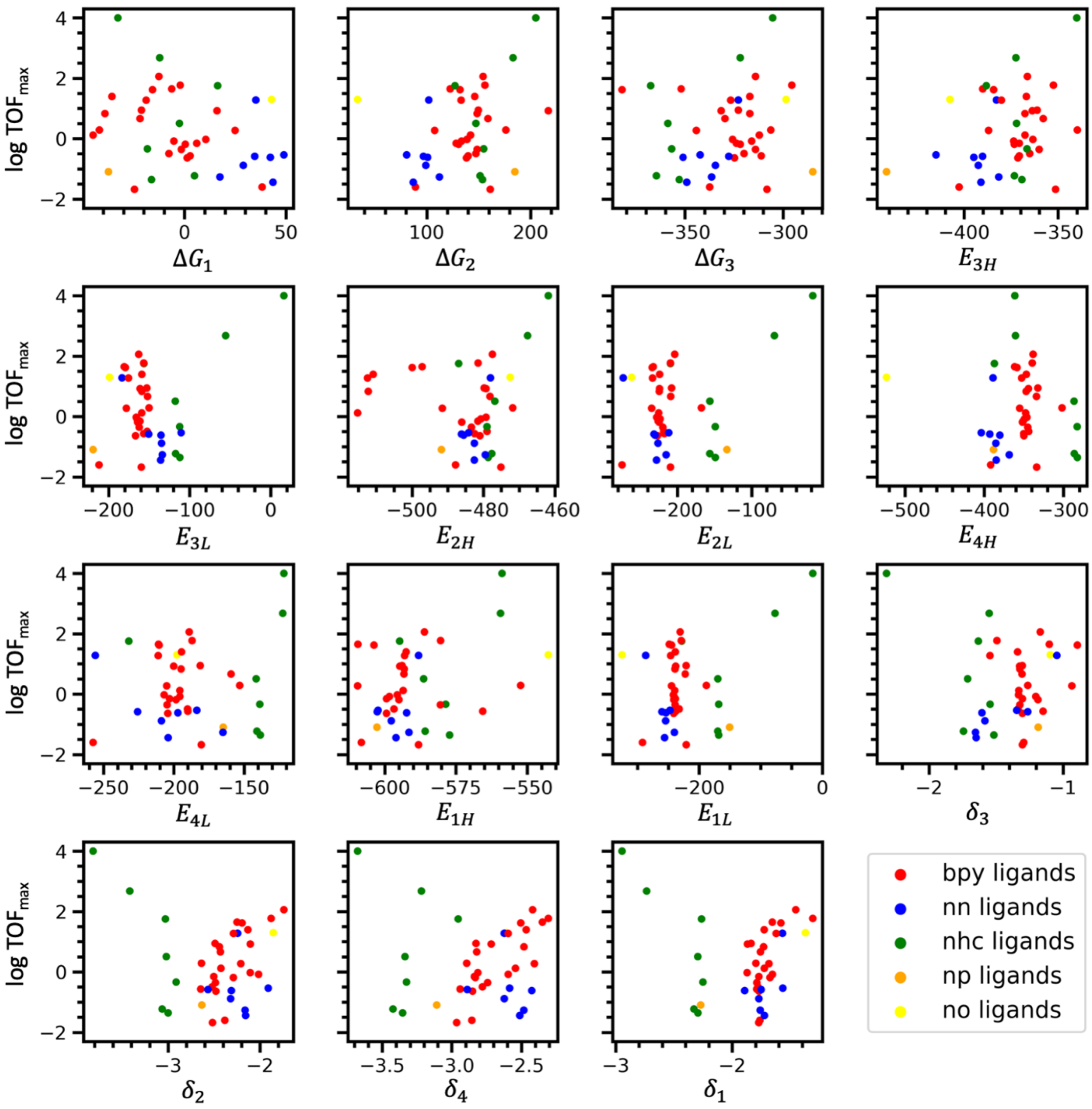

Figure S6. TOF_{max} plotted as a function of the 15 primary features. All energies are given in kJ/mol and all partial charges are multiples of the charge of one electron.

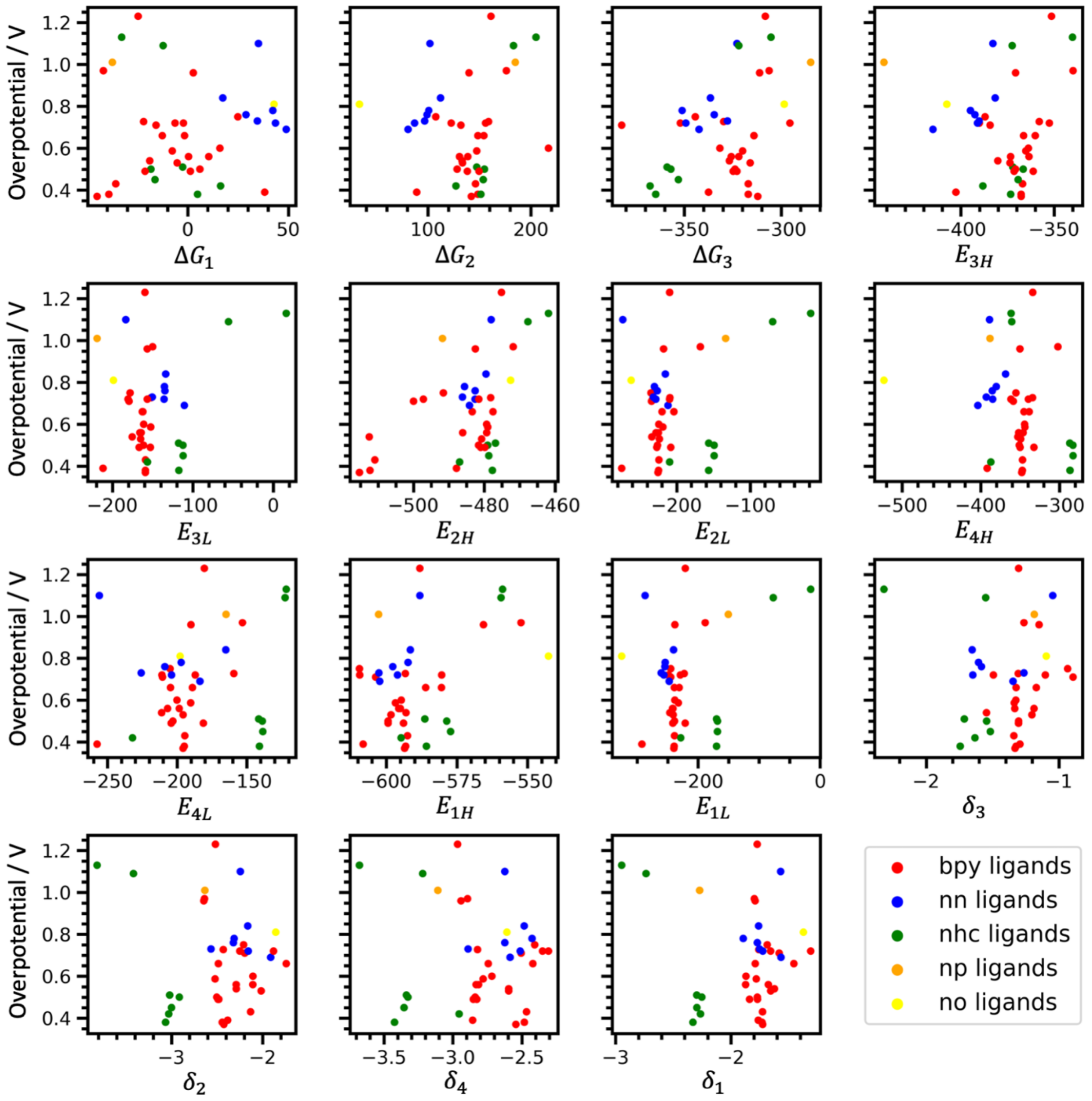

Figure S7. Overpotential plotted as a function of the 15 primary features. All energies are given in units of kJ/mol and all partial charges are multiples of the charge of one electron.

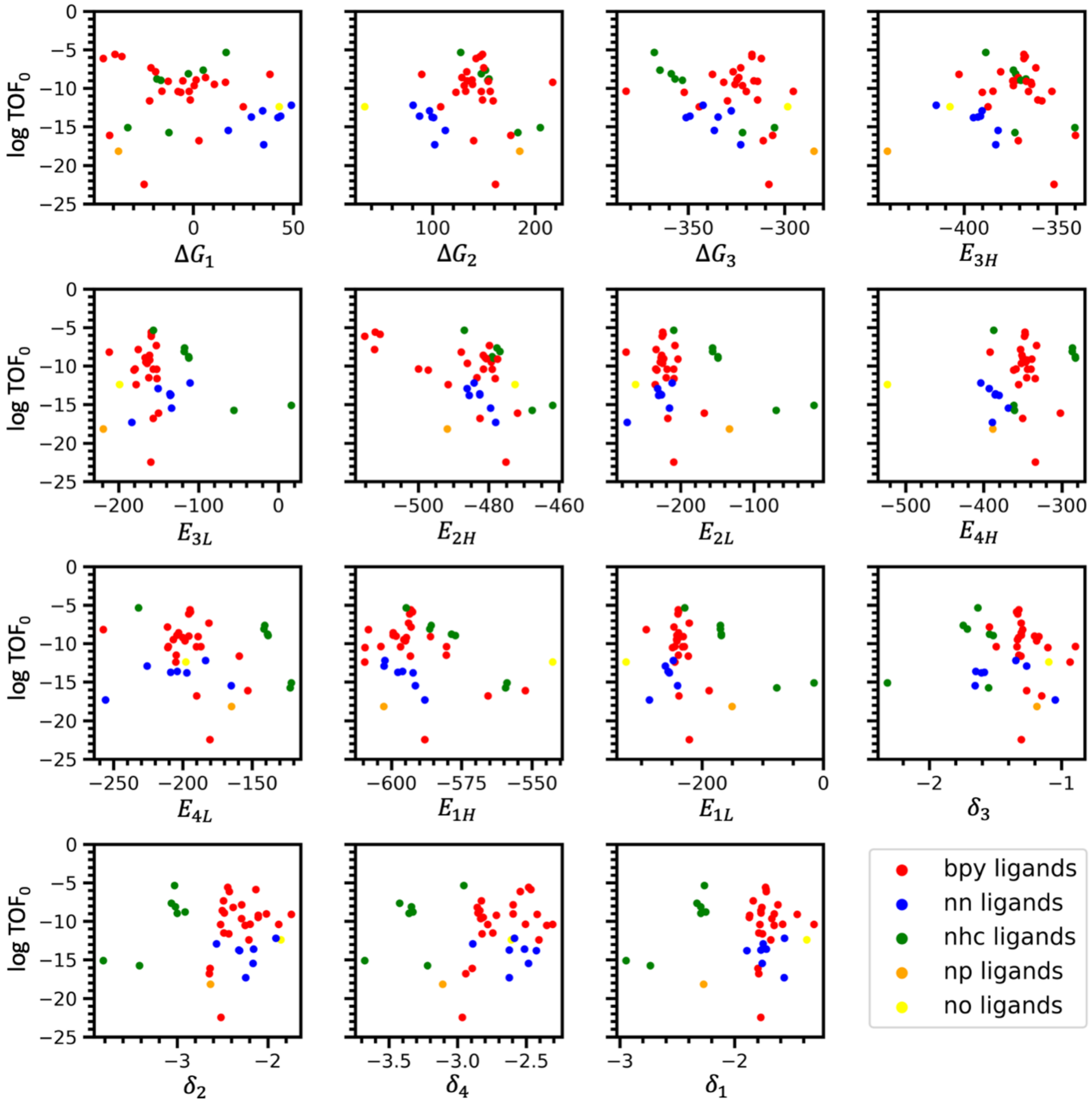

Figure S8. TOF_0 plotted as a function of the 15 primary features. All energies are given in kJ/mol and all partial charges are multiples of the charge of one electron.

List of SISSO Models

The top three SISSO models for each data subset are listed in **Tables S1-S3**. Increasing the feature complexity and removing outliers gives better performing models, as shown in **Tables S4-S5** for TOF_0.

Table S1. Best three SISSO models for TOF_{max} trained on datasets split according to solvent and ligand inclusion criteria. Ligand **3** is removed as an outlier. Parenthesis after the models indicate the Pearson correlation coefficient of that model.

Model inclusion criteria			SISSO models		
Ligand Type	Solvent Type	Solvent Concentration	SISSO 1	SISSO 2	SISSO 3
bpy	H_2O	2.7 M	$\boldsymbol{E_{4H} * \delta_4}$ **(0.81)**	$E_{4H} - E_{2H}$ (0.78)	δ_3/E_{3L}(0.77)
bpy	H_2O	0.79 – 7.2 M	$\boldsymbol{E_{4H} * \delta_4}$ **(0.82)**	$E_{3H} * \delta_4$ (0.80)	$E_{1L} * \delta_4$ (0.78)
nn	H_2O	2.7 M	$\Delta G_2 - E_{2H}$ (0.81)	$\Delta G_1 - \Delta G_2$ (0.77)	$E_{4H} - E_{2H}$ (0.77)
nn	H_2O	0.17 – 7.2 M	$E_{4H} * \delta_1$ (0.72)	$\delta_1/\Delta G_2$ (0.69)	$\boldsymbol{E_{4H} * \delta_4}$ **(0.66)**
nhc	H_2O	0.55 – 2.7 M	$E_{4H} * \delta_1$ (0.95)	$E_{4H}/\Delta G_3$ (0.95)	$\boldsymbol{E_{4H} * \delta_4}$ **(0.93)**
all	H_2O	2.7 M	$\lvert\Delta G_3 - E_{4H}\rvert$ (0.55)	$E_{3H} - E_{2H}$ (0.55)	$\Delta G_2 * E_{2L}$ (0.54)
all	H_2O	0.17 – 7.2 M	$E_{1L} * \delta_1$ (0.58)	$E_{2L} * \delta_1$ (0.54)	$\Delta G_1/E_{2L}$ (0.54)
bpy	TFE	0.5 – 1.9 M	$E_{3H} - E_{2L}$ (0.99)	$E_{2H} - E_{2L}$ (0.99)	E_{1H} (0.97)
nn	TFE	0.5 – 2.5 M	$\Delta G_1 - E_{1L}$ (0.72)	$E_{4L} * \delta_4$ (0.68)	E_{2H}/E_{3H} (0.67)
all	TFE	0.5 – 3.1 M	$E_{3H} - E_{2H}$ (0.78)	$\delta_4/\Delta G_3$ (0.74)	$\boldsymbol{E_{4H} * \delta_4}$ **(0.74)**

Table S2. Best three SISSO models for overpotential trained on datasets split according to solvent and ligand inclusion criteria. Ligand **3** and Ligand **32** are removed as outliers. Parenthesis after the models indicate the Pearson correlation coefficient of that model.

Model inclusion criteria			SISSO models		
Ligand Type	Solvent Type	Solvent Concentration	SISSO 1	SISSO 2	SISSO 3
bpy	H_2O	2.7 M	E_{1H} (0.89)	$\Delta G_3 - E_{1H}$ (0.88)	$\boldsymbol{E_{1H} + E_{2H}}$ **(0.88)**
bpy	H_2O	0.79 – 7.2 M	$E_{4H} - E_{2L}$ (0.65)	$\boldsymbol{E_{1H} + E_{2H}}$ **(0.65)**	$E_{3H} - E_{2L}$ (0.65)
nn	H_2O	2.7 M	$E_{1H} + E_{3L}$ (0.79)	$\Delta G_3 - E_{1H}$ (0.75)	$\boldsymbol{E_{1H} + E_{2H}}$ **(0.74)**
nn	H_2O	0.17 – 7.2 M	$\boldsymbol{E_{1H} + E_{2H}}$ **(0.60)**	$\Delta G_1 + E_{1H}$ (0.59)	$\Delta G_3 - E_{1H}$ (0.53)
nhc	H_2O	0.55 – 2.7 M	$E_{4H} - E_{4L}$ (0.98)	$\Delta G_2/\delta_4$ (0.98)	ΔG_3 (0.97)
all	H_2O	2.7 M	$E_{4H} - E_{4L}$ (0.63)	$\Delta G_3 + E_{2H}$ (0.62)	$\Delta G_3 + E_{1H}$ (0.59)
all	H_2O	0.17 – 7.2 M	$E_{4H} - E_{2L}$ (0.59)	$E_{4H} - E_{1L}$ (0.59)	E_{2L}/E_{4H} (0.53)

bpy	TFE	0.5 – 1.9 M	$E_{3H} * E_{4L}$ (0.97)	$E_{1L} * E_{4H}$ (0.97)	E_{1L} (0.96)
nn	TFE	0.5 – 2.5 M	$E_{3H} + E_{4H}$ (0.98)	$\Delta G_3 * E_{1L}$ (0.97)	E_{1L} (0.94)
all	TFE	0.5 – 3.1 M	$\Delta G_1 - E_{3H}$ (0.97)	$E_{2L}/\Delta G_2$ (0.97)	$E_{3H} + E_{4H}$ (0.95)

Table S3. Best three SISSO models for TOF_0 trained on datasets split according to solvent and ligand inclusion criteria. Ligand **3** and Ligand **32** are removed as outliers. Parenthesis after the models indicate the Pearson correlation coefficient of that model.

Model inclusion criteria			SISSO models		
Ligand Type	Solvent Type	Solvent Concentration	SISSO 1	SISSO 2	SISSO 3
bpy	H_2O	2.7 M	$\boldsymbol{E_{1H} + E_{2H}\ (0.89)}$	δ_4/E_{1H} (0.88)	E_{1H} (0.84)
bpy	H_2O	0.79 – 7.2 M	$\boldsymbol{E_{1H} + E_{2H}\ (0.70)}$	$\Delta G_1 + E_{2L}$ (0.64)	E_{1H} (0.57)
nn	H_2O	2.7 M	$\Delta G_3 - E_{1H}$ (0.74)	E_{2L}/E_{1L} (0.72)	$\boldsymbol{E_{1H} + E_{2H}\ (0.67)}$
nn	H_2O	0.17 – 7.2 M	$\Delta G_1 + E_{1H}$ (0.73)	$E_{3H} - E_{1H}$ (0.61)	$\boldsymbol{E_{1H} + E_{2H}\ (0.60)}$
nhc	H_2O	0.55 – 2.7 M	E_{4H}/E_{4L} (0.99)	$\delta_4/\Delta G_2$ (0.99)	E_{1H} (0.98)
all	H_2O	2.7 M	$E_{4H} - E_{4L}$ (0.65)	$\Delta G_1 + E_{2H}$ (0.59)	$E_{3H} - E_{1H}$ (0.57)
all	H_2O	0.17 – 7.2 M	$\lvert E_{4H} - E_{2H}\rvert$ (0.50)	$E_{3H} - E_{2L}$ (0.49)	E_{2H} (0.46)
bpy	TFE	0.5 – 1.9 M	$E_{1H} + E_{2L}$ (0.98)	$E_{4L} * E_{2H}$ (0.97)	$\Delta G_1 + E_{1H}$ (0.94)
nn	TFE	0.5 – 2.5 M	$E_{3H} + E_{4H}$ (0.96)	$\Delta G_2 + E_{4H}$ (0.96)	$E_{1L} + E_{2H}$ (0.94)
all	TFE	0.5 – 3.1 M	$\Delta G_1 - E_{1L}$ (0.96)	$\Delta G_2 + E_{1L}$ (0.96)	E_{1L} (0.91)

Table S4. Best two SISSO models for TOF_{max} trained on datasets split according to solvent and ligand inclusion criteria. Ligand **3** is removed as an outlier. Up to three features are allowed per model. Parenthesis after the models indicate the Pearson correlation coefficient of that model.

Model inclusion criteria			SISSO models	
Ligand Type	Solvent Type	Solvent Concentration	SISSO 1	SISSO 2
bpy	H_2O	2.7 M	$E_{2H} + \lvert\Delta G_3 - E_{4H}\rvert$ (0.94)	$\Delta G_3 + E_{2H} - E_{4H}$ (0.92)
bpy	H_2O	0.79 – 7.2 M	$\delta_4/(\Delta G_3 - \Delta G_2)$ (0.85)	$(E_{4H} * \delta_4)/\Delta G_3$ (0.83)
nn	H_2O	2.7 M	$(E_{4H} - E_{2H})/\delta_2$ (0.82)	$\Delta G_2 - E_{2H}$ (0.81)

nn	H_2O	0.17 – 7.2 M	$\delta_1/(\Delta G_3 - \Delta G_2)$ (0.80)	$\delta_1/(\Delta G_2 - E_{2H})$ (0.79)
all	H_2O	2.7 M	$\Delta G_1/(E_{4H} - E_{4L})$ (0.65)	$\Delta G_1 + \lvert\Delta G_3 - E_{4H}\rvert$ (0.63)
all	H_2O	0.17 – 7.2 M	$(\Delta G_2 - E_{4H})/E_{3H}$ (0.67)	$\delta_4 * \delta_1 * E_{1H}$ (0.66)
all	TFE	0.5 – 3.1 M	$(E_{3H} + E_{4H})/\Delta G_2$ (0.81)	$\delta_4 * E_{4L}/E_{1H}$ (0.81)

Table S5. Best two SISSO models for overpotential trained on datasets split according to solvent and ligand inclusion criteria. Ligand **3** and Ligand **32** are removed as outliers. Up to three features are allowed per model. Parenthesis after the models indicate the Pearson correlation coefficient of that model.

Model inclusion criteria			SISSO models	
Ligand Type	Solvent Type	Solvent Concentration	SISSO 1	SISSO 2
bpy	H_2O	2.7 M	$\Delta G_1 + E_{1H} + E_{4L}$ (0.93)	$E_{1H} - E_{1L} + E_{4L}$ (0.90)
bpy	H_2O	0.79 – 7.2 M	$\Delta G_3 - E_{2L} - E_{1H}$ (0.74)	$\Delta G_1 + E_{1H} + E_{4L}$ (0.74)
nn	H_2O	2.7 M	$E_{2L} * E_{1H}/E_{1L}$ (0.86)	$E_{1H} - E_{1L} + E_{2L}$(0.85)
nn	H_2O	0.17 – 7.2 M	$\Delta G_3 - E_{2L} - E_{1H}$ (0.70)	$E_{3H} - E_{2H} - E_{1H}$ (0.69)
all	H_2O	2.7 M	$E_{4H} - E_{4L} - E_{1H}$ (0.79)	$\Delta G_3/(E_{4H} - E_{4L})$ (0.77)
all	H_2O	0.17 – 7.2 M	$\delta_1 * E_{2L}/E_{4H}$ (0.67)	$E_{1L} * \lvert E_{4H} - E_{2H}\rvert$ (0.66)
all	TFE	0.5 – 3.1 M	$E_{4H} * (\Delta G_1 - E_{2L})$ (0.98)	$E_{4H} * (\Delta G_1 - E_{1L})$ (0.98)

Full Dataset of 55 Mn-Carbonyl Catalysts

The full dataset containing the ligand identifiers, experimental figures of merit, relative uncertainty, and primary features is given in the table below. The table reads from right to left, and each row continues on the following page with the ligand number corresponding to the ligand structures given in **Table 1**. All energies are given in kJ/mol. A legend for the table headers is given in **Table S6**.

Table S6. Heading legend for the full dataset.

Column Heading	Explanation
ligand_number	Ligand Number (**Table 1**) for the Mn-Carbonyl complex
ligand_name	Name of the ligand in the Mn-Carbonyl complex

ligand_type	Type of ligand in the Mn-Carbonyl complex. bpy (nn) = bipyridine-based ligands, nn = di-imine ligands (ex. bipyridine), nc = N-heterocyclic carbene ligands, no = bidentate ligands coordinating to Mn via N and O atoms, and np = bidentate ligands coordinating to Mn via N and P atoms.
relative_uncertainty	Relative uncertainty in the TOF_{max} discussed in Section 2.3.1. This number represents how different the revised TOF_{max} is from the calculated TOF_{max} (in multiples of the calculated TOF_{max}). Low numbers correspond to lower uncertainty (1 = no uncertainty), whereas higher numbers correspond to higher uncertainty (e.g. 5 = 4 * TOF_{max} of uncertainty).
E_cat2	Half-wave potential (V) of the catalyst
FE_CO	Faradaic efficiency (%) of the catalyst for CO
proton_donor_name	Type of proton donor reported for the experimental cyclic voltammograms
reported_TOFmax	TOF_{max} reported for the Mn-Carbonyl catalyst if available (nr = not reported)
G_CO2	Feature ΔG_1
pKa_H	pKa corresponding to the proton dissociation energy ΔG_2
G_H+	Feature ΔG_2
E_CO	Equilibrium electrode potential (V) corresponding to ΔG_3
G_CO	Feature ΔG_3
Mn_3CO_partial_charge	Feature δ_3
Mn_4CO_partial_charge	Feature δ_4
Mn_H_partial_charge	Feature δ_1
Mn_CO2_partial_charge	Feature δ_2
Mn_3CO_homo	Feature E_{3H}
Mn_3CO_lumo	Feature E_{3L}
Mn_4CO_homo	Feature E_{4H}
Mn_4CO_lumo	Feature E_{4L}
Mn_H_homo	Feature E_{1H}
Mn_H_lumo	Feature E_{1L}
Mn_CO2_homo	Feature E_{2H}
Mn_CO2_lumo	Feature E_{2L}

ligand_number	ligand_name	ligand_type	TOFmax	relative_uncertainty	E_cat2	log_TOF0	FE_CO	proton_donor_name
1	bpy	bpy (nn)	0.320	1.727	-1.937	-10.422	100.000	H2O
2	2ch3-bpy	bpy (nn)	4.600	1.796	-2.077	-11.632	100.000	H2O
3	2oh-bpy	bpy (nn)	0.021	6.603	-2.580	-22.479	6.000	H2O
4	2cooh-bpy	bpy (nn)	0.025	4.111	-1.740	-8.198	nr	H2O
5	sme-bpy	bpy (nn)	142.000	1.285	-1.930	-7.656	100.000	TFE
6	4cf3-bpy	bpy (nn)	0.000	nr	nr	nr	nr	H2O
7	5cf3-bpy	bpy (nn)	0.000	nr	nr	nr	nr	H2O
8	nme2cf3-bpy	bpy (nn)	0.270	3.626	-2.310	-16.804	84.000	H2O
9	cn-bpy	bpy (nn)	0.000	nr	nr	nr	nr	H2O
10	nme2-bpy	bpy (nn)	1.930	1.839	-2.320	-16.119	90.000	H2O
11	tbu-bpy	bpy (nn)	8.740	1.286	-1.840	-7.345	nr	H2O
12	mes-bpy	bpy (nn)	114.000	1.645	-2.010	-9.105	98.000	H2O
13	meoph-bpy	bpy (nn)	58.700	3.030	-2.070	-10.408	61.000	H2O
14	6etph-bpy	bpy (nn)	120.000	2.541	-2.020	-9.251	94.000	TFE
15	ph2-bpy	bpy (nn)	0.940	1.303	-1.910	-9.497	72.000	H2O
16	ph2oh-bpy	bpy (nn)	8.350	1.306	-1.950	-9.225	64.000	H2O
17	im3-bpy	bpy (nn)	1.870	2.907	-2.100	-12.412	73.000	H2O
18	im1-bpy	bpy (nn)	41.600	2.631	-2.060	-10.388	70.000	H2O
19	im4-bpy	bpy (nn)	44.300	1.715	-2.070	-10.530	72.000	H2O
20	conme2-bpy	bpy (nn)	0.830	1.654	-1.880	-9.044	87.000	H2O
21	conhme-bpy	bpy (nn)	0.650	1.041	-1.910	-9.657	90.000	H2O
22	anl1-bpy	bpy (nn)	50.300	2.897	-1.950	-8.445	83.000	TFE
23	anl2-bpy	bpy (nn)	11.200	3.323	-1.990	-9.774	76.000	TFE
24	anl3-bpy	bpy (nn)	16.700	4.247	-2.050	-10.615	93.000	TFE
25	4ohph-bpy	bpy (nn)	0.700	1.402	-1.850	-8.611	57.000	H2O
26	5ohph-bpy	bpy (nn)	0.230	1.191	-1.840	-8.925	nr	H2O
27	6ohph-bpy	bpy (nn)	6.750	2.797	-1.730	-5.597	77.000	H2O
28	meohph-bpy	bpy (nn)	24.800	1.375	-1.780	-5.877	nr	H2O
29	fohph-bpy	bpy (nn)	1.310	1.801	-1.720	-6.140	64.000	H2O
30	pd-bpy	bpy (nn)	18.700	3.642	-1.890	-7.860	90.000	H2O
31	pt-bpy	bpy (nn)	0.440	3.267	-2.010	-11.518	74.000	H2O
32	k3-tpy	nn	3.690	1.795	-1.820	-7.381	93.000	PhOH
33	phen	nn	18.400	2.874	-1.980	-9.389	64.000	TFE
34	me-phen	nn	14.150	2.383	-2.010	-10.011	15.000	TFE
35	bqn	nn	18.940	1.267	-2.450	-17.325	98.000	H2O
36	imp	nn	0.260	1.948	-2.080	-12.930	nr	H2O
37	ipimp	nn	0.130	2.695	-2.110	-13.739	nr	H2O
38	tbimp	nn	0.036	3.383	-2.070	-13.620	nr	H2O
39	tbiep	nn	0.054	2.564	-2.190	-15.473	nr	H2O
40	dipimp	nn	0.240	4.018	-2.130	-13.811	nr	H2O
41	ipr-dab	nn	0.290	2.758	-2.040	-12.206	nr	H2O
42	pyrox	nn	44.800	1.024	-1.930	-8.157	99.000	TFE
43	benzox	nn	2.950	2.116	-1.630	-4.265	nr	TFE
44	cnc	nc	33.800	5.639	-2.230	-13.353	94.000	TFE
45	ome-cnc	nc	16.400	4.763	-2.500	-18.233	107.000	TFE
46	coome-cnc	nc	0.740	1.029	-1.590	-4.190	nr	TFE
47	et-impy	nc	0.460	1.246	-1.850	-8.793	67.000	H2O
48	et-bimpy	nc	3.200	1.785	-1.860	-8.120	48.000	H2O
49	me-impy	nc	0.044	3.156	-1.800	-8.967	35.000	H2O
50	me-bimpy	nc	0.059	1.451	-1.730	-7.655	nr	H2O
51	et-bimnpy	nc	56.400	1.311	-1.770	-5.352	72.000	H2O
52	bis-menhc	nc	9960.000	3.131	-2.480	-15.112	98.000	H2O
53	mes-nhc	nc	475.000	5.042	-2.440	-15.757	95.000	H2O
54	2me-oqn	no	19.800	2.130	-2.160	-12.402	88.000	H2O
55	k2-pn	np	0.080	3.571	-2.360	-18.177	96.000	H2O

ligand_number	proton_donor_concentration	reported_TOFmax	G_CO2	pKa_H	G_H+	E_CO	G_CO
1	2.700	nr	-7.675	25.600	147.476	-1.770	-319.867
2	2.700	nr	-21.913	27.600	158.997	-1.670	-329.610
3	2.700	nr	-24.639	28.000	161.302	-1.890	-308.175
4	2.700	nr	38.272	15.460	89.062	-1.590	-337.405
5	0.500	460.000	-2.918	23.670	136.357	-1.750	-321.816
6	2.700	nr	32.718	17.500	100.814	-1.720	-324.739
7	2.700	nr	17.490	27.400	157.845	-1.820	-314.995
8	2.700	nr	2.817	24.300	139.987	-1.860	-311.098
9	2.700	nr	46.956	13.600	78.346	-1.570	-339.353
10	2.700	nr	-41.907	30.600	176.280	-1.910	-306.227
11	2.650	120.000	-21.206	26.000	149.780	-1.740	-322.790
12	7.200	nr	-12.623	26.800	154.389	-1.830	-314.021
13	6.300	258.000	-2.100	27.100	156.117	-2.020	-295.509
14	2.000	700.000	6.746	23.200	133.650	-1.810	-315.970
15	2.700	nr	10.502	24.100	138.835	-1.710	-325.713
16	2.700	nr	16.046	37.700	217.181	-1.650	-331.559
17	4.300	nr	24.942	18.700	107.726	-1.520	-344.225
18	4.300	nr	-15.753	22.940	132.152	-1.130	-382.223
19	5.050	nr	-6.301	21.300	122.704	-1.440	-352.019
20	5.500	nr	-5.251	23.200	133.650	-1.810	-315.970
21	5.500	nr	0.434	22.700	130.770	-1.750	-321.816
22	1.900	901.000	-14.642	24.500	141.139	-1.740	-322.790
23	0.790	245.000	-9.432	27.100	156.117	-1.860	-311.098
24	1.900	296.000	-10.330	28.400	163.606	-1.800	-316.944
25	2.700	nr	6.079	22.300	128.465	-1.730	-323.764
26	2.700	nr	1.373	24.020	138.374	-1.720	-324.739
27	2.700	nr	-39.181	25.800	148.628	-1.800	-316.944
28	1.700	nr	-35.747	25.400	146.324	-1.800	-316.944
29	1.700	nr	-45.037	24.700	142.291	-1.850	-312.072
30	2.700	81.000	-18.883	23.130	133.247	-1.700	-326.687
31	2.700	nr	-1.545	25.800	148.628	-1.830	-314.021
32	2.400	nr	7.503	21.300	122.704	-1.910	-306.227
33	2.000	75.000	-3.595	25.000	144.019	-1.740	-322.790
34	2.500	67.000	-7.230	25.500	146.900	-1.660	-330.584
35	0.170	14.000	35.172	17.700	101.966	-1.740	-322.790
36	2.600	nr	34.636	16.800	96.781	-1.690	-327.661
37	2.600	nr	28.981	17.200	99.085	-1.620	-334.482
38	2.600	nr	43.624	15.100	86.988	-1.470	-349.096
39	2.600	nr	17.470	19.500	112.335	-1.600	-336.430
40	2.600	nr	42.311	17.500	100.814	-1.450	-351.045
41	2.700	nr	48.976	14.000	80.651	-1.540	-342.276
42	1.000	40.000	1.323	25.100	144.595	-1.730	-323.764
43	1.000	nr	12.724	22.100	127.313	-1.760	-320.841
44	3.100	454.000	-2.424	27.500	158.421	-1.750	-321.816
45	3.100	408.000	-15.652	29.700	171.095	-1.790	-317.918
46	3.100	nr	34.636	19.100	110.031	-2.040	-293.560
47	2.700	0.860	-18.278	26.900	154.965	-1.390	-356.891
48	2.700	4.500	-2.474	25.600	147.476	-1.370	-358.839
49	2.700	0.070	-16.258	26.700	153.813	-1.430	-352.994
50	2.700	0.080	4.938	26.300	151.508	-1.310	-364.685
51	2.700	42.000	16.359	22.100	127.313	-1.280	-367.608
52	0.560	320000.000	-32.819	35.600	205.083	-1.920	-305.252
53	0.550	3180.000	-12.229	31.800	183.193	-1.750	-321.816
54	0.170	44.100	42.917	5.800	33.412	-1.990	-298.432
55	2.700	nr	-37.454	32.110	184.978	-2.130	-284.792

ligand_number	Mn_3CO_partial_charge	Mn_4CO_partial_charge	Mn_H_partial_charge	Mn_CO2_partial_charge	Mn_3CO_homo	Mn_3CO_lumo
1	-1.336	-2.780	-1.780	-2.520	-365.136	-152.389
2	-1.306	-2.821	-1.758	-2.430	-357.938	-152.009
3	-1.303	-2.965	-1.772	-2.516	-351.354	-159.638
4	-1.293	-2.856	-1.763	-2.382	-402.692	-211.726
5	-1.238	-2.791	-1.759	-2.554	-367.611	-158.904
6	-1.235	-2.882	-1.760	-2.390	-395.265	-168.831
7	-1.219	-2.825	-1.709	-2.430	-390.306	-188.744
8	-1.149	-2.940	-1.790	-2.644	-370.646	-156.721
9	-1.263	-2.838	-1.726	-2.373	-406.890	-199.674
10	-1.263	-2.893	-1.797	-2.636	-339.775	-150.100
11	-1.304	-2.826	-1.838	-2.488	-361.172	-152.843
12	-1.170	-2.420	-1.455	-1.740	-366.359	-162.853
13	-1.493	-2.307	-1.309	-1.879	-352.512	-156.690
14	-1.583	-2.247	-1.681	-1.874	-361.454	-165.332
15	-1.332	-2.813	-1.872	-2.106	-363.455	-165.915
16	-1.324	-2.717	-1.869	-2.105	-363.771	-161.169
17	-0.934	-2.407	-1.685	-2.208	-387.033	-178.079
18	-0.893	-2.505	-1.582	-2.195	-384.333	-179.368
19	-1.102	-2.350	-1.656	-2.249	-390.111	-180.693
20	-1.203	-2.595	-1.655	-2.015	-373.649	-164.978
21	-1.187	-2.831	-1.676	-2.289	-373.326	-164.306
22	-0.998	-2.619	-1.755	-2.397	-371.107	-160.185
23	-1.250	-2.877	-1.762	-2.423	-363.526	-155.035
24	-1.288	-2.874	-1.756	-2.418	-362.167	-153.654
25	-1.303	-2.838	-1.771	-2.502	-370.673	-161.377
26	-1.303	-2.851	-1.770	-2.479	-371.425	-166.802
27	-1.322	-2.482	-1.731	-2.441	-367.661	-159.092
28	-1.339	-2.466	-1.726	-2.131	-366.900	-158.998
29	-1.330	-2.543	-1.725	-2.424	-367.641	-159.126
30	-1.545	-2.595	-1.623	-2.288	-380.189	-175.443
31	-1.322	-2.743	-1.789	-2.484	-360.092	-162.200
32	-0.850	-2.050	-1.151	-1.830	-353.241	-169.334
33	-1.212	-2.913	-1.798	-2.674	-368.073	-175.695
34	-0.947	-2.679	-1.667	-2.378	-370.328	-165.926
35	-1.046	-2.623	-1.569	-2.243	-382.823	-183.444
36	-1.263	-2.889	-1.752	-2.565	-390.212	-150.572
37	-1.583	-2.623	-1.771	-2.319	-392.491	-134.733
38	-1.648	-2.512	-1.724	-2.156	-391.081	-135.811
39	-1.653	-2.483	-1.760	-2.162	-381.601	-133.790
40	-1.605	-2.426	-1.893	-2.311	-394.884	-135.355
41	-1.345	-2.585	-1.566	-1.910	-415.022	-110.518
42	-1.387	-2.875	-1.823	-2.584	-364.669	-139.650
43	-1.381	-2.764	-1.722	-2.410	-378.363	-173.519
44	-1.883	-2.563	-2.133	-2.570	-341.367	-72.782
45	-1.924	-2.696	-2.168	-2.651	-333.597	-83.342
46	-1.710	-3.231	-2.168	-2.463	-365.742	-144.905
47	-1.544	-3.326	-2.251	-2.912	-366.566	-112.290
48	-1.712	-3.337	-2.295	-3.019	-372.108	-117.747
49	-1.516	-3.354	-2.294	-2.999	-369.288	-112.058
50	-1.743	-3.423	-2.328	-3.064	-373.294	-117.371
51	-1.632	-2.954	-2.263	-3.029	-388.224	-156.501
52	-2.318	-3.677	-2.946	-3.815	-340.081	16.176
53	-1.551	-3.219	-2.735	-3.416	-372.465	-55.622
54	-1.095	-2.608	-1.372	-1.855	-407.572	-198.780
55	-1.185	-3.108	-2.270	-2.632	-441.392	-219.140

ligand_number	Mn_4CO_homo	Mn_4CO_lumo	Mn_H_homo	Mn_H_lumo	Mn_CO2_homo	Mn_CO2_lumo
1	-343.875	-190.206	-596.745	-232.356	-478.989	-218.678
2	-334.124	-159.247	-593.248	-222.581	-478.153	-209.175
3	-333.878	-180.416	-588.132	-220.857	-475.101	-209.542
4	-391.828	-257.461	-608.150	-292.131	-487.797	-274.581
5	-345.146	-193.706	-586.140	-233.495	-484.633	-210.342
6	-386.425	-233.497	-608.000	-274.254	-486.729	-257.086
7	-396.430	-246.879	-606.461	-282.313	-484.466	-262.572
8	-350.136	-190.016	-565.595	-238.156	-482.472	-217.721
9	-400.675	-259.020	-612.282	-301.370	-488.773	-277.156
10	-301.907	-153.091	-552.338	-188.202	-471.803	-167.735
11	-332.361	-181.160	-593.890	-221.013	-479.791	-207.991
12	-338.399	-189.004	-586.061	-230.893	-477.490	-203.769
13	-339.438	-186.975	-580.354	-229.181	-481.495	-209.615
14	-340.791	-192.020	-585.870	-230.707	-476.591	-203.741
15	-346.130	-206.892	-595.579	-242.097	-479.267	-227.987
16	-344.234	-200.138	-594.682	-238.254	-479.282	-224.267
17	-355.256	-205.092	-609.458	-244.954	-491.499	-234.827
18	-358.621	-210.504	-603.732	-244.947	-499.972	-234.089
19	-361.619	-210.908	-609.359	-248.717	-497.189	-232.626
20	-349.237	-195.686	-598.322	-241.013	-480.707	-223.412
21	-351.745	-198.439	-594.996	-241.388	-486.055	-224.811
22	-343.289	-190.947	-575.471	-232.080	-489.079	-218.754
23	-342.294	-191.237	-566.387	-232.031	-476.593	-217.475
24	-341.027	-190.077	-563.104	-229.458	-476.190	-215.144
25	-350.989	-203.061	-599.348	-238.724	-481.553	-226.054
26	-349.959	-204.276	-599.351	-241.533	-480.968	-226.916
27	-347.143	-194.816	-593.136	-239.346	-512.259	-224.619
28	-346.889	-194.464	-592.474	-238.767	-510.881	-224.114
29	-347.807	-195.724	-593.507	-239.788	-515.241	-225.372
30	-352.826	-211.132	-593.014	-246.393	-512.464	-233.629
31	-345.052	-204.787	-580.427	-239.045	-483.349	-219.856
32	-332.717	-203.024	-518.347	-226.409	-453.121	-206.500
33	-335.805	-188.920	-595.603	-231.006	-472.134	-214.975
34	-330.795	-187.566	-591.637	-223.478	-483.116	-199.704
35	-388.852	-255.844	-588.123	-286.924	-478.018	-273.051
36	-392.906	-225.802	-602.576	-260.626	-486.122	-231.638
37	-385.308	-208.821	-597.677	-254.574	-482.535	-226.293
38	-384.837	-204.021	-596.020	-256.243	-482.552	-228.259
39	-368.412	-165.029	-591.477	-240.365	-479.466	-215.394
40	-380.284	-197.102	-592.289	-254.083	-485.525	-230.206
41	-403.700	-183.578	-602.297	-247.495	-484.141	-211.774
42	-347.704	-170.761	-583.320	-228.539	-482.204	-200.929
43	-366.770	-209.787	-597.024	-259.647	-488.263	-234.266
44	-328.662	-171.546	-498.304	-160.419	-452.305	-142.532
45	-331.115	-155.966	-497.121	-138.760	-450.720	-121.323
46	-361.722	-192.144	-505.535	-240.899	-456.605	-224.774
47	-282.803	-138.834	-578.569	-168.143	-479.002	-149.081
48	-286.731	-141.396	-586.347	-169.584	-476.803	-156.327
49	-282.601	-138.451	-577.202	-168.180	-478.678	-149.015
50	-286.432	-140.861	-585.807	-169.643	-477.670	-156.408
51	-387.152	-232.035	-594.735	-228.393	-486.921	-209.553
52	-361.404	-121.675	-558.853	-15.406	-461.836	-18.060
53	-360.578	-122.473	-559.373	-76.762	-467.646	-69.683
54	-523.216	-197.835	-542.634	-325.333	-472.521	-261.710
55	-388.256	-164.833	-602.685	-150.391	-491.750	-133.402

FOTW Analysis Plots for the Mn-Carbonyl Catalysts

This section presents the forward trace of the cyclic voltammogram of each of the 55 Mn-carbonyl complexes under catalytic conditions, taken from the references given in **Table 1** for each complex. In addition, all relevant experimental conditions are given for each catalyst. The optimized structure of the $[Mn(CO)_3L_2(CO_2)]^-$ intermediate is also shown. Using FOTW analysis, the slopes were used to calculate the TOF_{max} using **Equation 33**. For each complex, the cyclic voltammograms were copied from the literature using DataThief.[22]

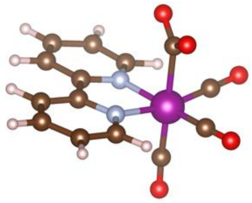

Active Catalyst	[Mn]	[CO_2]	Solvent	Proton Donor	Electrolyte	Ref.	FE_{CO}
$[Mn(CO)_3(bpy)]^-$	1 mM	Sat.	MeCN	5% H_2O	0.1 M Bu_4NClO_4	Ag/$AgNO_3$ (10 mM)	100 %

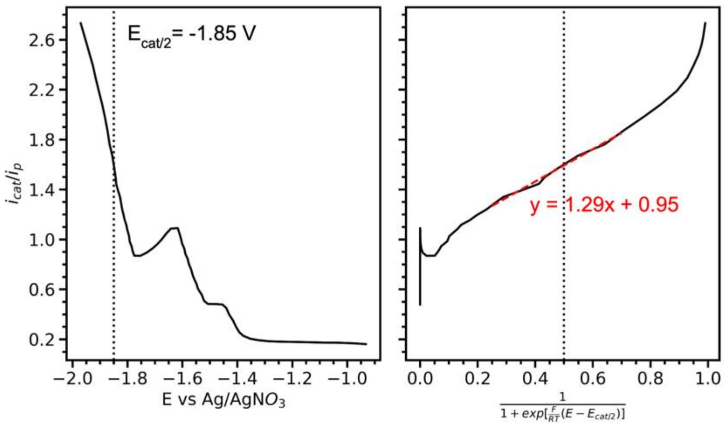

FOTW slope	1.29
$E_{cat/2}$	-1.85 V
$E^0_{CO/CO2}$	n.r.
i_p	n.r.
Scan Rate	0.1 V/s
Reported TOF_{max}	n.r.
Calc. TOF_{max}	0.32 /s

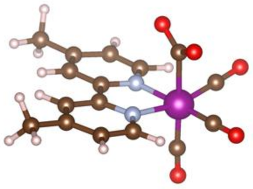

Active Catalyst	[Mn]	[CO_2]	Solvent	Proton Donor	Electrolyte	Ref.	FE_{CO}
$[Mn(CO)_3((dm\text{-}bpy))]^-$	1 mM	Sat.	MeCN	5% H_2O	0.1 M Bu_4NClO_4	Ag/$AgNO_3$ (10 mM)	100%

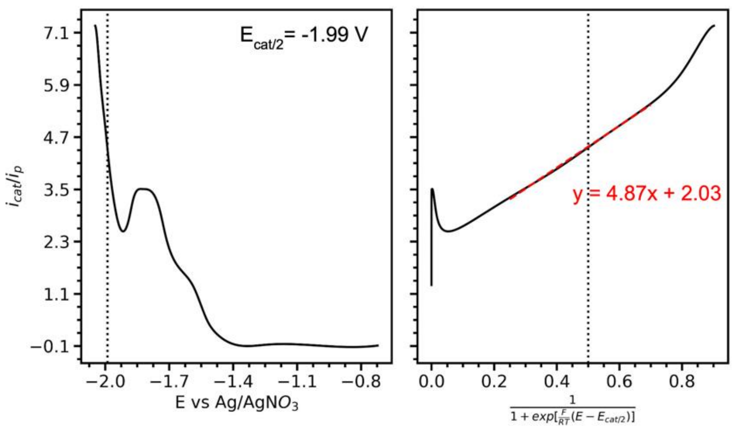

FOTW slope	4.87
$E_{cat/2}$	-1.99 V
$E^0_{CO/CO2}$	n.r.
i_p	n.r.
Scan Rate	0.1 V/s
Reported TOF_{max}	n.r.
Calc. TOF_{max}	4.6 /s

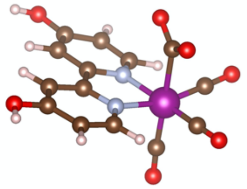

Active Catalyst	[Mn]	[CO_2]	Solvent	Proton Donor	Electrolyte	Ref.	FE_{CO}
$[Mn(CO)_3((OH\text{-}bpy))]^-$	1 mM	Sat.	MeCN	5% H_2O	0.1 M NBu_4PF_6	SCE	45 %

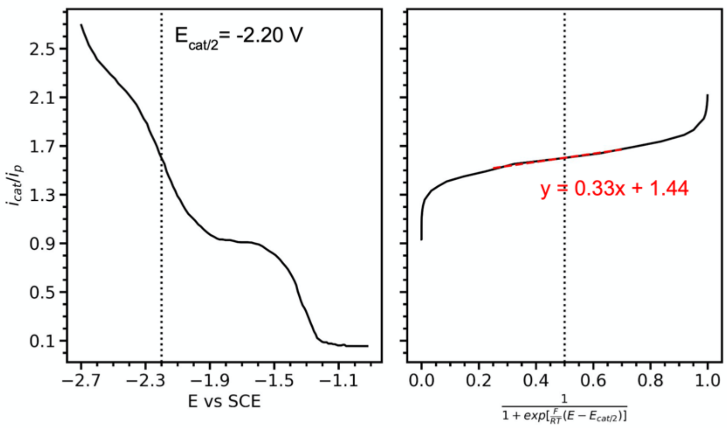

FOTW slope	0.36
$E_{cat/2}$	-2.20 V
$E^0_{CO/CO2}$	n.r.
i_p	0.39 mA/cm^2
Scan Rate	0.1 V/s
Reported TOF_{max}	n.r.
Calc. TOF_{max}	0.021 /s

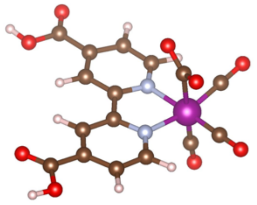

Active Catalyst	[Mn]	$[CO_2]$	Solvent	Proton Donor	Electrolyte	Ref.	FE_{CO}
$[Mn(CO)_3((COOH\text{-}bpy)]^-$	1 mM	Sat.	MeCN	5% H_2O	0.1 M NBu_4PF_6	SCE	n.r.

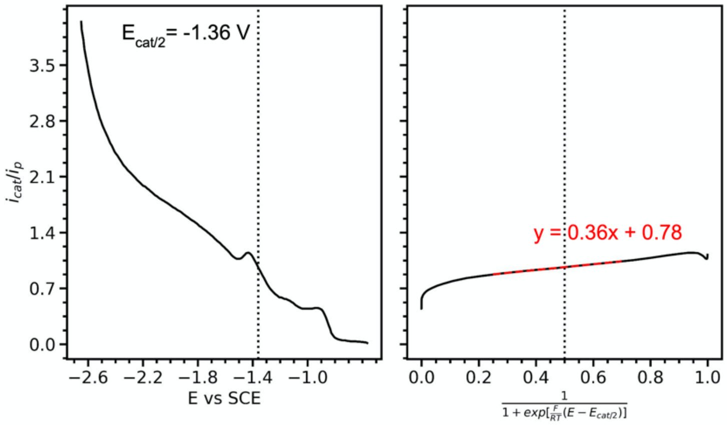

FOTW slope	0.36
$E_{cat/2}$	-1.36 V
$E^0_{CO/CO2}$	n.r.
i_p	0.24 mA/cm^2
Scan Rate	0.1 V/s
Reported TOF_{max}	n.r.
Calc. TOF_{max}	0.025 /s

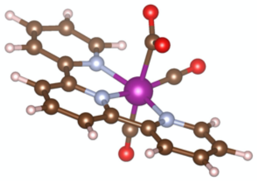

Active Catalyst	[Mn]	$[CO_2]$	Solvent	Proton Donor	Electrolyte	Ref.	FE_{CO}
$[Mn(CO)_2(k^3\text{-}tpy)]^-$	1 mM	Sat.	MeCN	2.4 M PhOH	0.1 M NBu_4PF_6	Fc/Fc^+	93 %

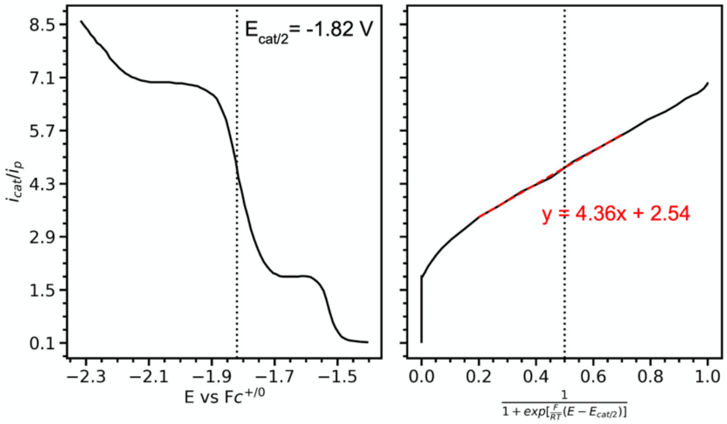

FOTW slope	4.36
$E_{cat/2}$	-1.82 V
$E^0_{CO/CO2}$	n.r.
i_p	0.31 mA/cm^2
Scan Rate	0.1 V/s
Reported TOF_{max}	n.r.
Calc. TOF_{max}	3.69 /s

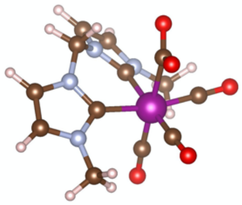

Active Catalyst	[Mn]	$[CO_2]$	Solvent	Proton Donor	Electrolyte	Ref.	FE_{CO}
$[Mn(CO)_3(\text{bis-MeNHC})]^-$	1 mM	Sat.	MeCN	0.56 M H_2O	0.1 M NBu_4PF_6	Fc/Fc^+	98 %

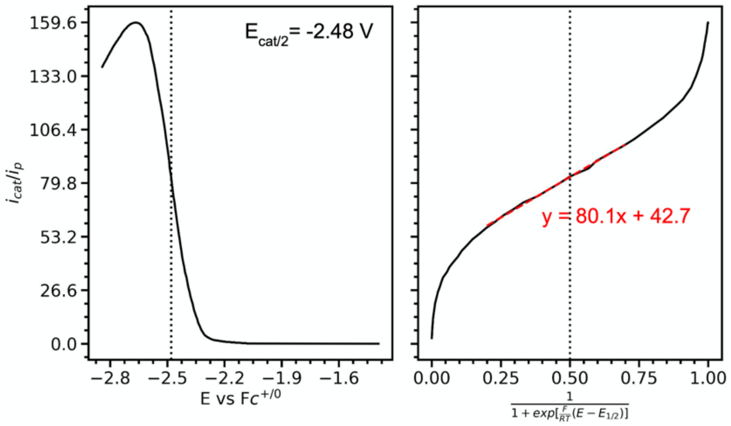

FOTW slope	80.1
$E_{cat/2}$	-2.48 V
$E^0_{CO/CO2}$	n.r.
i_p	0.44 mA/cm^2
Scan Rate	0.1 V/s
Reported TOF_{max}	320000 /s
Calc. TOF_{max}	9960 /s

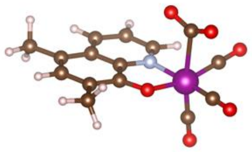

Active Catalyst	[Mn]	$[CO_2]$	Solvent	Proton Donor	Electrolyte	Ref.	FE_{CO}
$[Mn(CO)_3(Me_2OQN)]^-$ *$[Mn(CO)_3(Me_2OQN)]^{2-}$ also reported	1 mM	Sat.	MeCN	0.17 M H_2O	0.1 M NBu_4PF_6	Fc/Fc^+	88 %

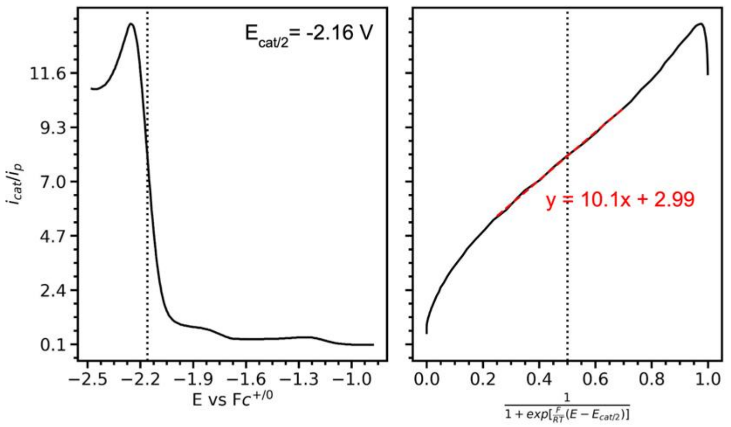

FOTW slope	10.1
$E_{cat/2}$	-2.16 V
$E^0_{CO/CO2}$	n.r.
i_p	n.r.
Scan Rate	0.1 V/s
Reported TOF_{max}	44.1 /s
Calc. TOF_{max}	19.8 /s

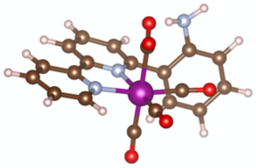

Active Catalyst	[Mn]	[CO_2]	Solvent	Proton Donor	Electrolyte	Ref.	FE_{CO}
$[Mn(CO)_3(anl1\text{-}bpy)]^-$	0.5 mM	Sat.	MeCN	15% TFE	0.1 M Bu_4NPF_6	Fc/Fc^+	83 %

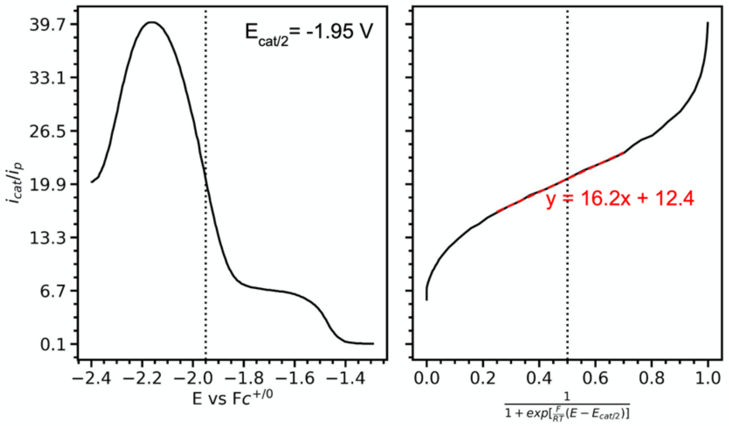

FOTW slope	16.2
$E_{cat/2}$	-1.95 V
$E^0_{CO/CO2}$	-1.36 V
i_p	15.5 μA
Scan Rate	0.1 V/s
Reported TOF_{max}	901 /s
Calc. TOF_{max}	50.9 /s

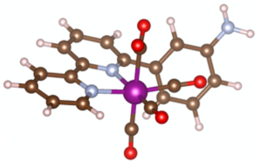

Active Catalyst	[Mn]	[CO_2]	Solvent	Proton Donor	Electrolyte	Ref.	FE_{CO}
$[Mn(CO)_3(anl2\text{-}bpy)]^-$	0.5 mM	Sat.	MeCN	6% TFE	0.1 M Bu_4NPF_6	Fc/Fc^+	76 %

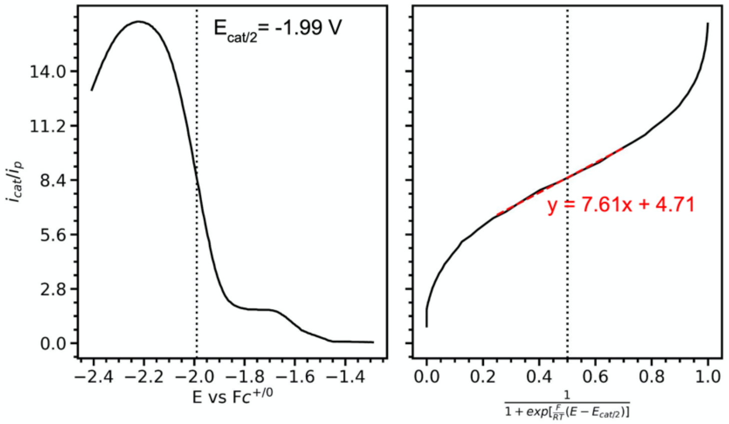

FOTW slope	7.61
$E_{cat/2}$	-1.99 V
$E^0_{CO/CO2}$	-1.36 V
i_p	22 μA
Scan Rate	0.1 V/s
Reported TOF_{max}	245.2 /s
Calc. TOF_{max}	11.2 /s

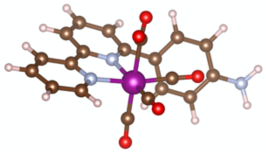

Active Catalyst	[Mn]	[CO_2]	Solvent	Proton Donor	Electrolyte	Ref.	FE_{CO}
$[Mn(CO)_3(anl3\text{-}bpy)]^-$	0.5 mM	Sat.	MeCN	15% TFE	0.1 M Bu_4NPF_6	Fc/Fc^+	93 %

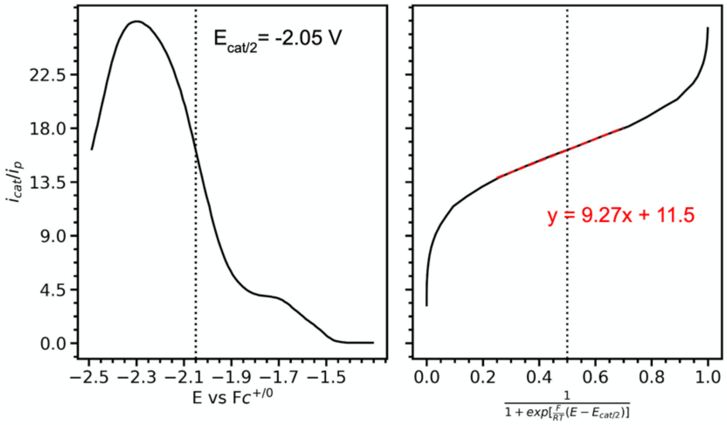

FOTW slope	9.27
$E_{cat/2}$	-2.05 V
$E^0_{CO/CO2}$	-1.36 V
i_p	14.3 μA
Scan Rate	0.1 V/s
Reported TOF_{max}	296.0 /s
Calc. TOF_{max}	16.7 /s

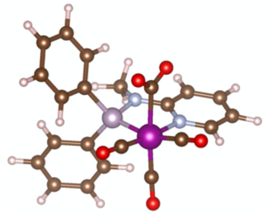

Active Catalyst	[Mn]	[CO_2]	Solvent	Proton Donor	Electrolyte	Ref.	FE_{CO}
$[Mn(CO)_3(k^2\text{-}PN)]^-$	1 mM	Sat.	MeCN	5% H_2O	0.1 M Bu_4NPF_6	Fc/Fc^+	96 %

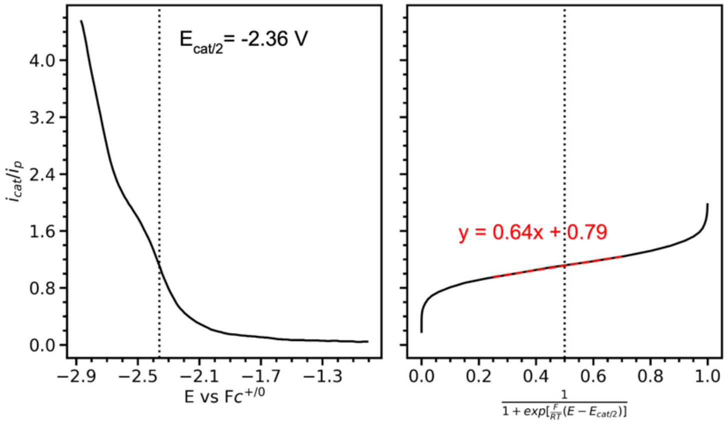

FOTW slope	0.64
$E_{cat/2}$	-2.36 V
$E^0_{CO/CO2}$	n.r.
i_p	26 μA
Scan Rate	0.1 V/s
Reported TOF_{max}	n.r.
Calc. TOF_{max}	0.08 /s

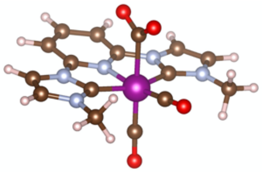

**Active Catalyst**	**[Mn]**	**$[CO_2]$**	**Solvent**	**Proton Donor**	**Electrolyte**	**Ref.**	**FE_{CO}**
$[Mn(CO)_3(CNC)]^-$	1 mM	Sat.	MeCN	“240 μL” TFE	0.1 M Bu_4NPF_6	Fc/Fc^+	94 %

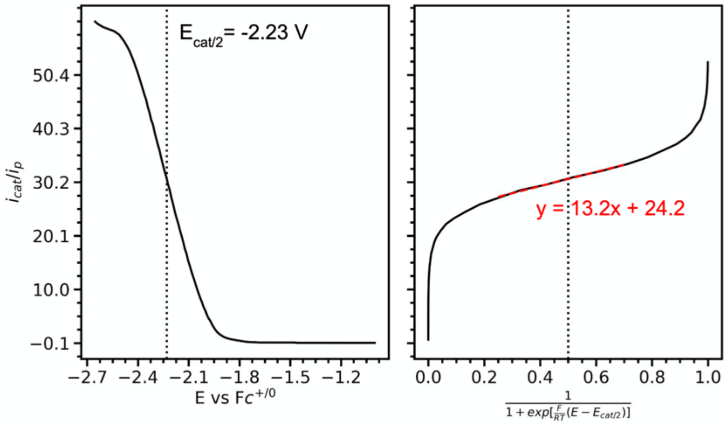

FOTW slope	13.2
$E_{cat/2}$	-2.23 V
$E^0_{CO/CO2}$	n.r.
i_p	0.45 mA/cm^2
Scan Rate	0.1 V/s
Reported TOF_{max}	454 /s
Calc. TOF_{max}	33.8 /s

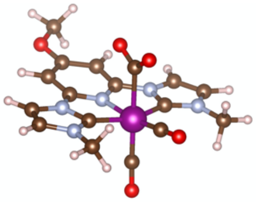

**Active Catalyst**	**[Mn]**	**$[CO_2]$**	**Solvent**	**Proton Donor**	**Electrolyte**	**Ref.**	**FE_{CO}**
$[Mn(CO)_3(CNC\text{-}OMe)]^-$	1 mM	Sat.	MeCN	“240 μL” TFE	0.1 M Bu_4NPF_6	Fc/Fc^+	107 %

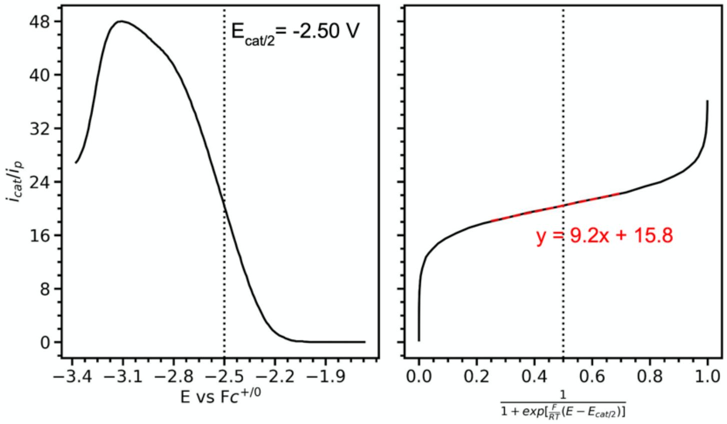

FOTW slope	9.2
$E_{cat/2}$	-2.50 V
$E^0_{CO/CO2}$	n.r.
i_p	0.44 mA/cm^2
Scan Rate	0.1 V/s
Reported TOF_{max}	408 /s
Calc. TOF_{max}	16.4 /s

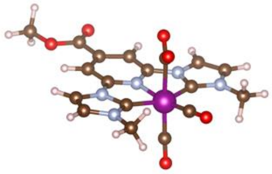

Active Catalyst	[Mn]	[CO_2]	Solvent	Proton Donor	Electrolyte	Ref.	FE_{CO}
$[Mn(CO)_3(CNC\text{-}COOMe)]^-$	1 mM	Sat.	MeCN	"240 µL" TFE	0.1 M Bu_4NPF_6	Fc/Fc$^+$	n.r.

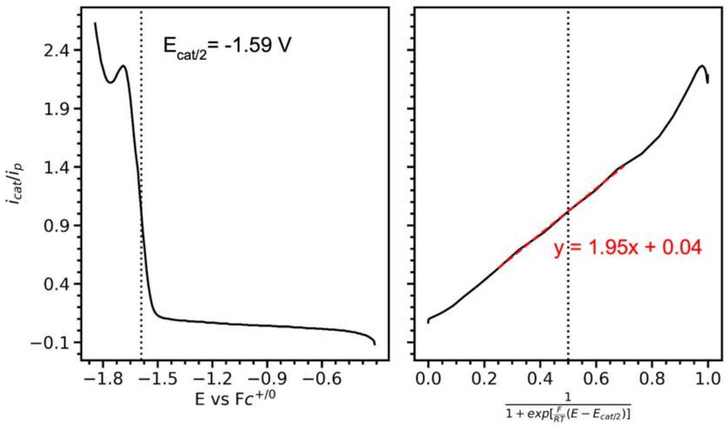

FOTW slope	1.95
$E_{cat/2}$	-1.59 V
$E^0_{CO/CO2}$	n.r.
i_p	0.26 mA/cm^2
Scan Rate	0.1 V/s
Reported TOF_{max}	n.r.
Calc. TOF_{max}	0.74 /s

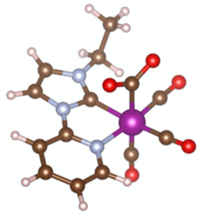

Active Catalyst	[Mn]	[CO_2]	Solvent	Proton Donor	Electrolyte	Ref.	FE_{CO}
$[Mn(CO)_3(Et\text{-}Im\text{-}py)]^-$	1 mM	Sat.	MeCN	5% H_2O	0.1 M Bu_4NClO_4	SCE	67 %

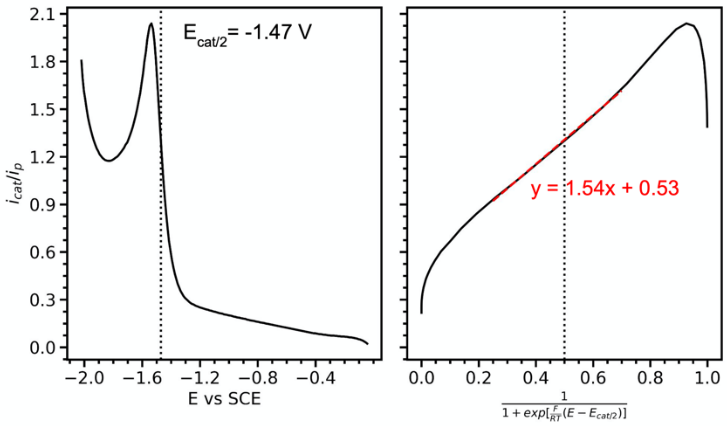

FOTW slope	1.54
$E_{cat/2}$	-1.47 V
$E^0_{CO/CO2}$	n.r.
i_p	0.69 mA/cm^2
Scan Rate	0.1 V/s
Reported TOF_{max}	0.86 /s
Calc. TOF_{max}	0.46 /s

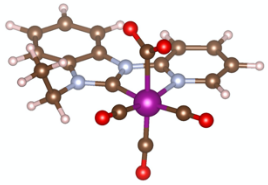

Active Catalyst	[Mn]	[CO_2]	Solvent	Proton Donor	Electrolyte	Ref.	FE_{CO}
$[Mn(CO)_3(Et\text{-}BIm\text{-}py)]^-$	1 mM	Sat.	MeCN	5% H_2O	0.1 M Bu_4NClO_4	Fc/Fc+	48 %

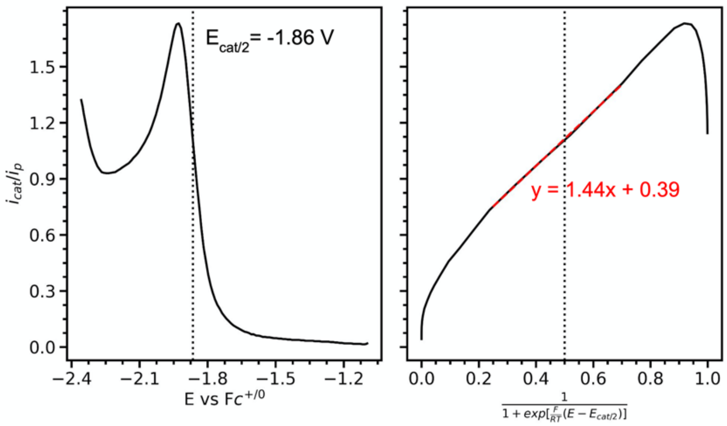

FOTW slope	1.44
$E_{cat/2}$	-1.86 V
$E^0_{CO/CO2}$	n.r.
i_p	0.50 mA/cm^2
Scan Rate	0.1 V/s
Reported TOF_{max}	4.5 /s
Calc. TOF_{max}	3.2 /s

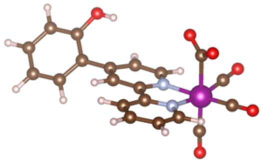

Active Catalyst	[Mn]	[CO_2]	Solvent	Proton Donor	Electrolyte	Ref.	FE_{CO}
$[Mn(CO)_3(4\text{-}HOPh\text{-}bpy)]^-$	1 mM	Sat.	MeCN	5% H_2O	0.1 M Bu_4NClO_4	SCE	57 %

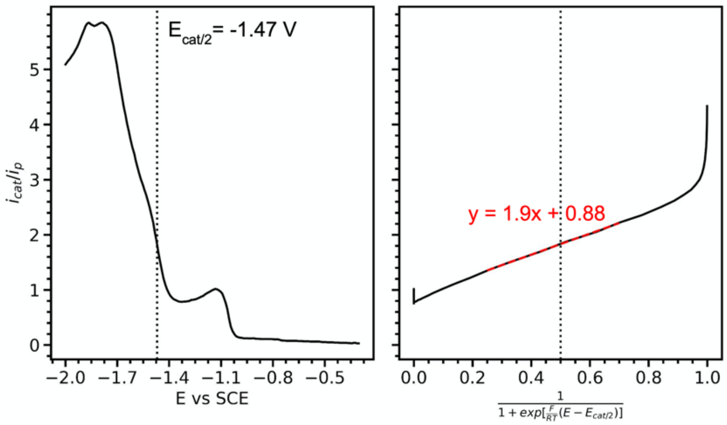

FOTW slope	1.90
$E_{cat/2}$	-1.47 V
$E^0_{CO/CO2}$	n.r.
i_p	0.50 mA/cm^2
Scan Rate	0.1 V/s
Reported TOF_{max}	n.r.
Calc. TOF_{max}	0.70 /s

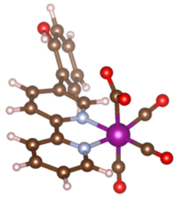

**Active Catalyst**	**[Mn]**	**[CO_2]**	**Solvent**	**Proton Donor**	**Electrolyte**	**Ref.**	**FE_{CO}**
$[Mn(CO)_3(5\text{-}HOPh\text{-}bpy)]^-$	1 mM	Sat.	MeCN	5% H_2O	0.1 M Bu_4NClO_4	SCE	n.r.

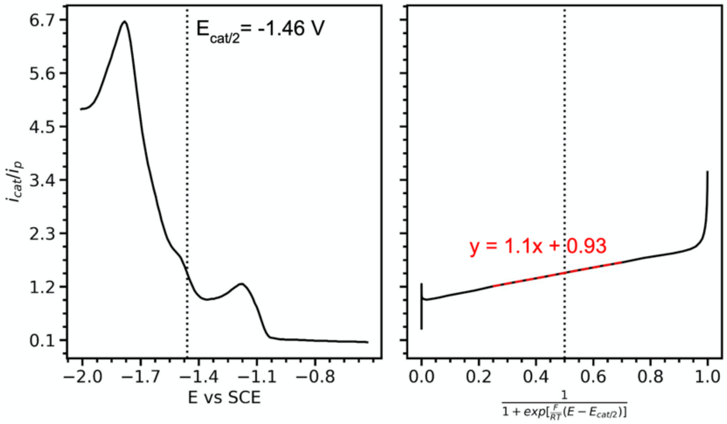

FOTW slope	1.1
$E_{cat/2}$	-1.46 V
$E^0_{CO/CO2}$	n.r.
i_p	0.45 mA/cm^2
Scan Rate	0.1 V/s
Reported TOF_{max}	n.r.
Calc. TOF_{max}	0.23 /s

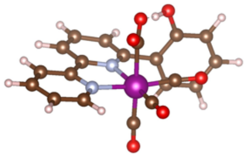

**Active Catalyst**	**[Mn]**	**[CO_2]**	**Solvent**	**Proton Donor**	**Electrolyte**	**Ref.**	**FE_{CO}**
$[Mn(CO)_3(6\text{-}HOPh\text{-}bpy)]^-$	1 mM	Sat.	MeCN	5% H_2O	0.1 M Bu_4NClO_4	SCE	77 %

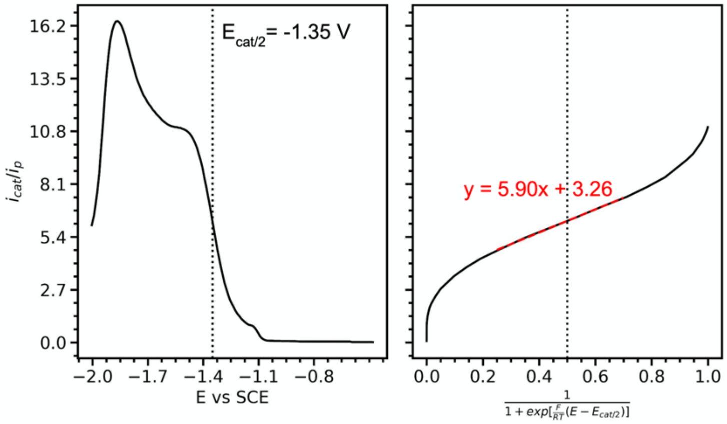

FOTW slope	5.9
$E_{cat/2}$	-1.35 V
$E^0_{CO/CO2}$	-0.86 V
i_p	0.58 mA/cm^2
Scan Rate	0.1 V/s
Reported TOF_{max}	n.r.
Calc. TOF_{max}	6.75 /s

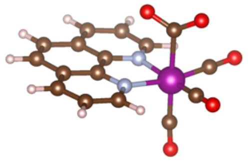

Active Catalyst	[Mn]	[CO_2]	Solvent	Proton Donor	Electrolyte	Ref.	FE_{CO}
$[Mn(CO)_3(Phen)]^-$	1 mM	Sat.	MeCN	2 M TFE	0.1 M Bu_4NPF_6	Fc/Fc^+	64 %

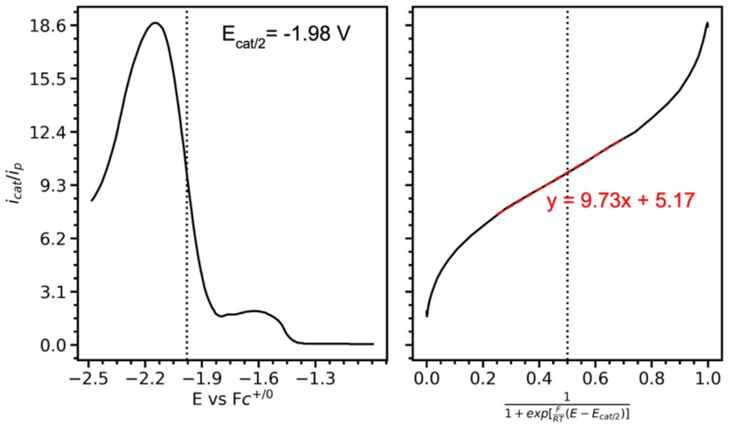

FOTW slope	9.73
$E_{cat/2}$	-1.98 V
$E^0_{CO/CO2}$	n.r.
i_p	n.r.
Scan Rate	0.1 V/s
Reported TOF_{max}	75 /s
Calc. TOF_{max}	18.4 /s

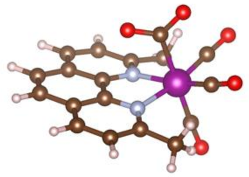

Active Catalyst	[Mn]	[CO_2]	Solvent	Proton Donor	Electrolyte	Ref.	FE_{CO}
$[Mn(CO)_3(Me\text{-}Phen)]^-$	1 mM	Sat.	MeCN	2.5 M TFE	0.1 M Bu_4NPF_6	Fc/Fc^+	15 %

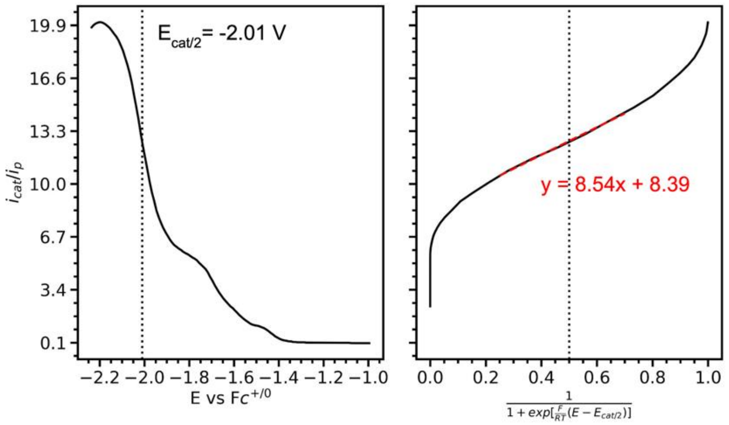

FOTW slope	8.54
$E_{cat/2}$	-2.01 V
$E^0_{CO/CO2}$	n.r.
i_p	n.r.
Scan Rate	0.1 V/s
Reported TOF_{max}	67 /s
Calc. TOF_{max}	14.15 /s

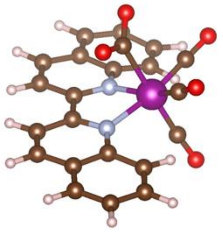

Active Catalyst	[Mn]	[CO_2]	Solvent	Proton Donor	Electrolyte	Ref.	FE_{CO}
$[Mn(CO)_3(Bqn)]^-$	1 mM	Sat.	MeCN	0.17 M H_2O	0.1 M Bu_4NPF_6	Fc/Fc^+	98 %

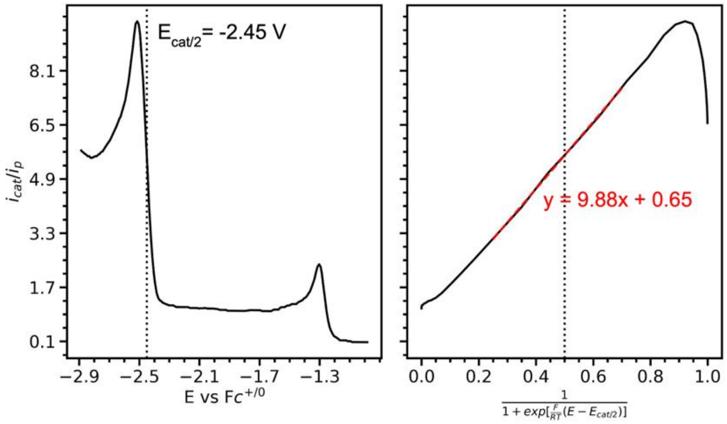

FOTW slope	9.88
$E_{cat/2}$	-2.45 V
$E^0_{CO/CO2}$	n.r.
i_p	n.r.
Scan Rate	0.1 V/s
Reported TOF_{max}	14 /s
Calc. TOF_{max}	18.94 /s

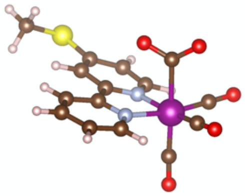

Active Catalyst	[Mn]	[CO_2]	Solvent	Proton Donor	Electrolyte	Ref.	FE_{CO}
$[Mn(CO)_3(MeS\text{-}bpy)]^-$	1 mM	Sat.	MeCN	0.5 M TFE	0.1 M Bu_4NPF_6	Fc/Fc^+	100 %

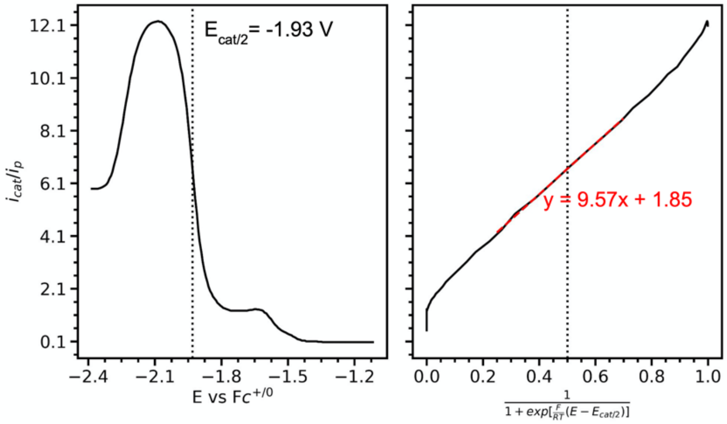

FOTW slope	9.57
$E_{cat/2}$	-1.93 V
$E^0_{CO/CO2}$	n.r.
i_p	0.45 mA/cm^2
Scan Rate	0.1 V/s
Reported TOF_{max}	460 /s
Calc. TOF_{max}	142 /s

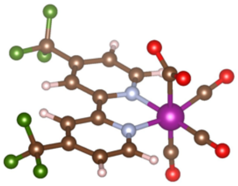

Active Catalyst	[Mn]	[CO_2]	Solvent	Proton Donor	Electrolyte	Ref.	FE_{CO}
$[Mn(CO)_3(4\text{-}CF_3\text{-bpy})]^-$	0.5 mM	Sat.	MeCN	n.r.	0.1 M Bu_4NPF_6	Fc/Fc^+	n.r.

Not catalytically active

FOTW slope	n.r.
$E_{cat/2}$	n.r.
$E^0_{CO/CO2}$	n.r.
i_p	n.r.
Scan Rate	0.2 V/s
Reported TOF_{max}	n.r.
Calc. TOF_{max}	0 /s

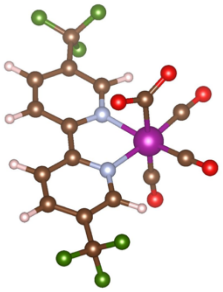

Active Catalyst	[Mn]	[CO_2]	Solvent	Proton Donor	Electrolyte	Ref.	FE_{CO}
$[Mn(CO)_3(5\text{-}CF_3\text{-bpy})]^-$	0.5 mM	Sat.	MeCN	n.r.	0.1 M Bu_4NPF_6	Fc/Fc^+	n.r.

Not catalytically active

FOTW slope	n.r.
$E_{cat/2}$	n.r.
$E^0_{CO/CO2}$	n.r.
i_p	n.r.
Scan Rate	0.2 V/s
Reported TOF_{max}	n.r.
Calc. TOF_{max}	0 /s

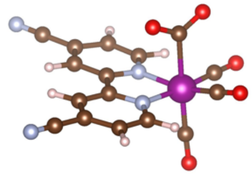

Active Catalyst	[Mn]	[CO_2]	Solvent	Proton Donor	Electrolyte	Ref.	FE_{CO}
$[Mn(CO)_3(CN\text{-}bpy)]^-$	0.5 mM	Sat.	MeCN	n.r.	0.1 M Bu_4NPF_6	Fc/Fc^+	n.r.

Not catalytically active

FOTW slope	n.r.
$E_{cat/2}$	n.r.
$E^0_{CO/CO2}$	n.r.
i_p	n.r.
Scan Rate	0.2 V/s
Reported TOF_{max}	n.r.
Calc. TOF_{max}	0 /s

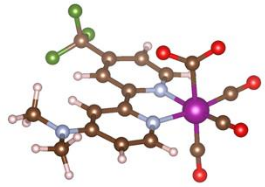

Active Catalyst	[Mn]	[CO_2]	Solvent	Proton Donor	Electrolyte	Ref.	FE_{CO}
$[Mn(CO)_3(NMe_2/CF_3\text{-}bpy)]^-$	0.5 mM	Sat.	MeCN	5% H_2O	0.1 M Bu_4NPF_6	Fc/Fc^+	84 %

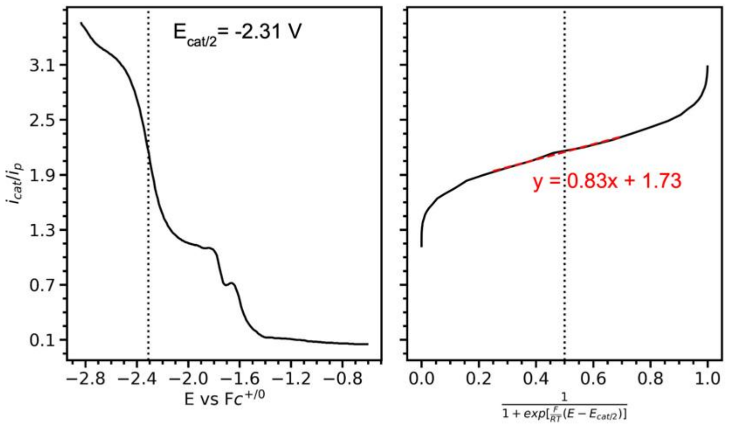

FOTW slope	0.83
$E_{cat/2}$	-2.31 V
$E^0_{CO/CO2}$	n.r.
i_p	2.42 μA
Scan Rate	0.2 V/s
Reported TOF_{max}	n.r.
Calc. TOF_{max}	0.27 /s

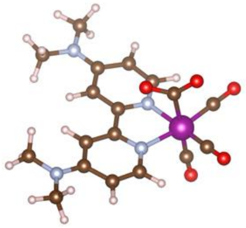

Active Catalyst	[Mn]	$[CO_2]$	Solvent	Proton Donor	Electrolyte	Ref.	FE_{CO}
$[Mn(CO)_3(NMe_2\text{-bpy})]^-$	0.5 mM	Sat.	MeCN	5% H_2O	0.1 M Bu_4NPF_6	Fc/Fc$^+$	90 %

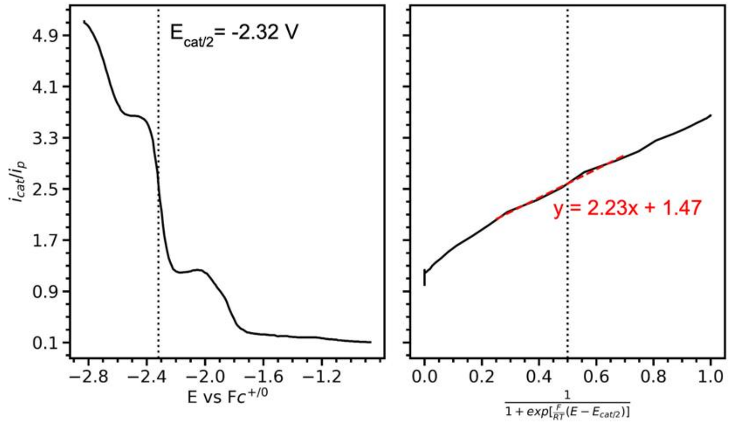

FOTW slope	2.23
$E_{cat/2}$	-2.32 V
$E^0_{CO/CO2}$	n.r.
i_p	1.84 μA
Scan Rate	0.2 V/s
Reported TOF_{max}	n.r.
Calc. TOF_{max}	1.93 /s

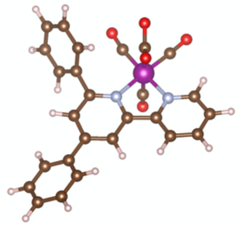

Active Catalyst	[Mn]	$[CO_2]$	Solvent	Proton Donor	Electrolyte	Ref.	FE_{CO}
$[Mn(CO)_3(Ph_2\text{-bpy})]^-$	0.5 mM	Sat.	MeCN	5% H_2O	0.1 M Bu_4NPF_6	Fc/Fc$^+$	72 %

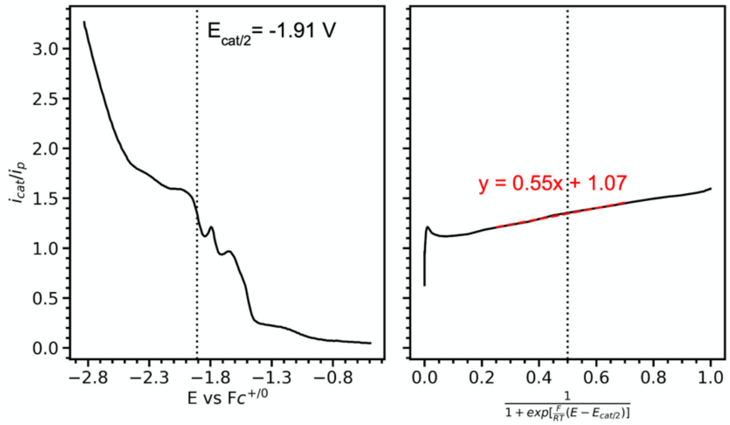

FOTW slope	0.55
$E_{cat/2}$	-1.91 V
$E^0_{CO/CO2}$	n.r.
i_p	1.83 μA
Scan Rate	0.2 V/s
Reported TOF_{max}	n.r.
Calc. TOF_{max}	0.94 /s

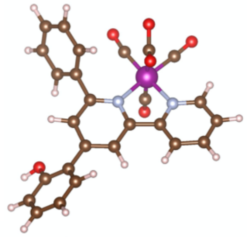

Active Catalyst	[Mn]	$[CO_2]$	Solvent	Proton Donor	Electrolyte	Ref.	FE_{CO}
$[Mn(CO)_3(Ph/PhOH\text{-}bpy)]^-$	0.5 mM	Sat.	MeCN	5% H_2O	0.1 M Bu_4NPF_6	Fc/Fc^+	64 %

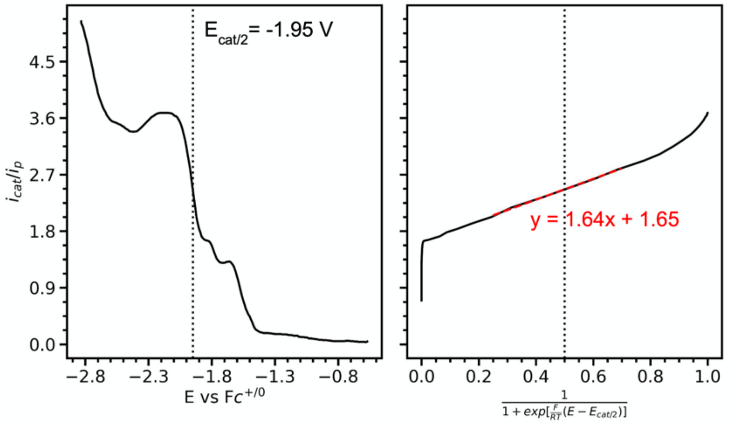

FOTW slope	1.64
$E_{cat/2}$	-1.95 V
$E^0_{CO/CO2}$	n.r.
i_p	2.03 μA
Scan Rate	0.2 V/s
Reported TOF_{max}	n.r.
Calc. TOF_{max}	8.35 /s

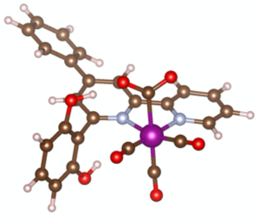

Active Catalyst	[Mn]	$[CO_2]$	Solvent	Proton Donor	Electrolyte	Ref.	FE_{CO}
$[Mn(CO)_3(pdbpy)]^-$	0.5 mM	Sat.	MeCN	2.7 M H_2O	0.1 M Bu_4NPF_6	SCE	90 %

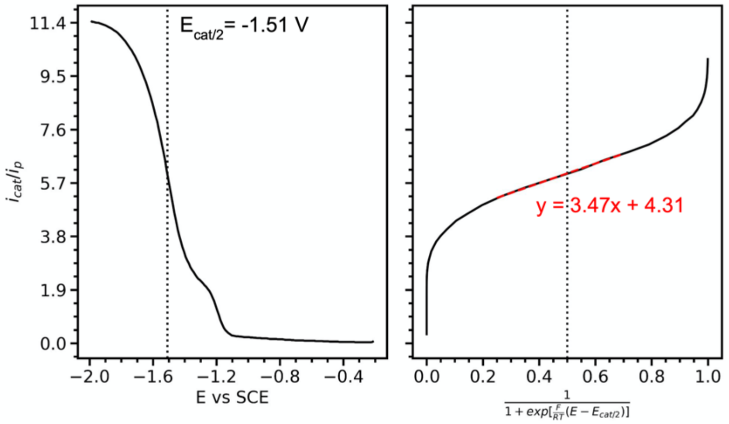

FOTW slope	3.47
$E_{cat/2}$	-1.51 V
$E^0_{CO/CO2}$	n.r.
i_p	1.5 μA
Scan Rate	0.1 V/s
Reported TOF_{max}	81 /s
Calc. TOF_{max}	18.7 /s

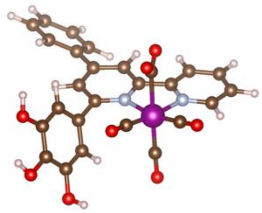

Active Catalyst	[Mn]	[CO_2]	Solvent	Proton Donor	Electrolyte	Ref.	FE_{CO}
$[Mn(CO)_3(ptbpy)]^-$	0.5 mM	Sat.	MeCN	2.7 M H_2O	0.1 M Bu_4NPF_6	SCE	74 %

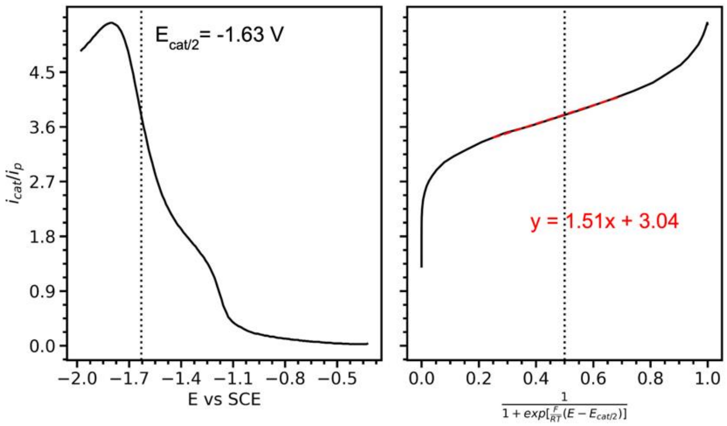

FOTW slope	1.51
$E_{cat/2}$	-1.63 V
$E^0_{CO/CO2}$	n.r.
i_p	1.7 μA
Scan Rate	0.1 V/s
Reported TOF_{max}	n.r.
Calc. TOF_{max}	0.44 /s

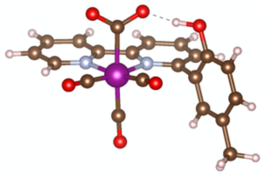

Active Catalyst	[Mn]	[CO_2]	Solvent	Proton Donor	Electrolyte	Ref.	FE_{CO}
$[Mn(CO)_3(Me\text{-}HOPh\text{-}bpy)]^-$	1 mM	Sat.	MeCN	3% H_2O	0.2 M Bu_4NPF_6	Fc/Fc^+	n.r.

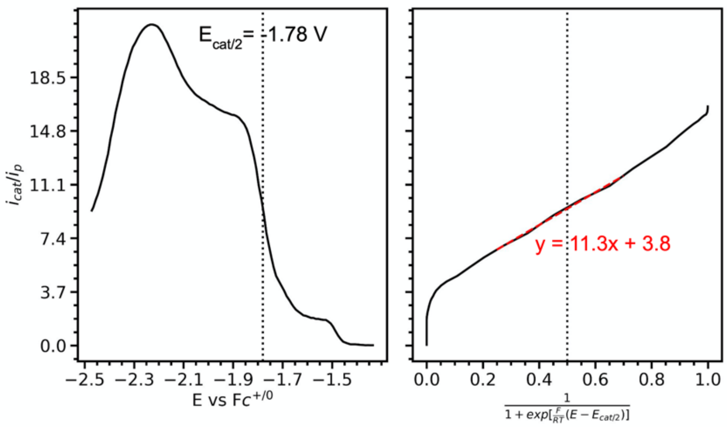

FOTW slope	11.3
$E_{cat/2}$	-1.78 V
$E^0_{CO/CO2}$	n.r.
i_p	12 μA
Scan Rate	0.1 V/s
Reported TOF_{max}	n.r.
Calc. TOF_{max}	24.8 /s

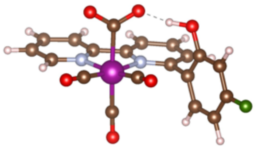

Active Catalyst	[Mn]	[CO_2]	Solvent	Proton Donor	Electrolyte	Ref.	FE_{CO}
$[Mn(CO)_3(F\text{-}HOPh\text{-}bpy)]^-$	1 mM	Sat.	MeCN	3% H_2O	0.2 M Bu_4NPF_6	Fc/Fc^+	64 %

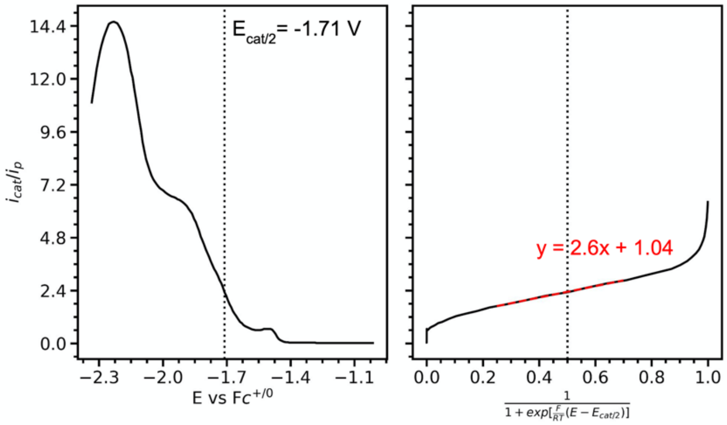

FOTW slope	2.6
$E_{cat/2}$	-1.72 V
$E^0_{CO/CO2}$	n.r.
i_p	33 μA
Scan Rate	0.1 V/s
Reported TOF_{max}	n.r.
Calc. TOF_{max}	1.31 /s

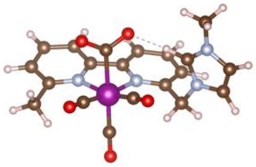

Active Catalyst	[Mn]	[CO_2]	Solvent	Proton Donor	Electrolyte	Ref.	FE_{CO}
$[Mn(CO)_3(Im1\text{-}bpy)]$	1 mM	Sat.	MeCN	4.27 M H_2O	0.1 M Bu_4NPF_6	Fc/Fc^+	70 %

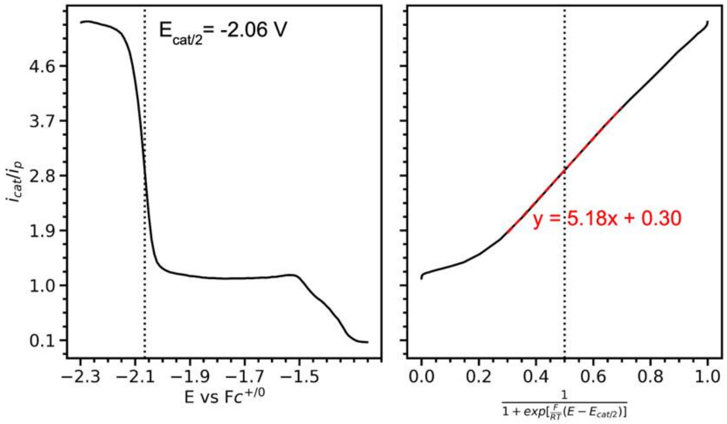

FOTW slope	5.18
$E_{cat/2}$	-2.06 V
$E^0_{CO/CO2}$	n.r.
i_p	0.61 mA/cm^2
Scan Rate	0.1 V/s
Reported TOF_{max}	n.r.
Calc. TOF_{max}	41.6 /s

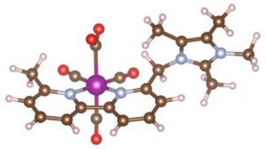

Active Catalyst	[Mn]	[CO_2]	Solvent	Proton Donor	Electrolyte	Ref.	FE_{CO}
[Mn(CO)$_3$(Im3-bpy)]	1 mM	Sat.	MeCN	4.27 M H_2O	0.1 M Bu_4NPF_6	Fc/Fc$^+$	73 %

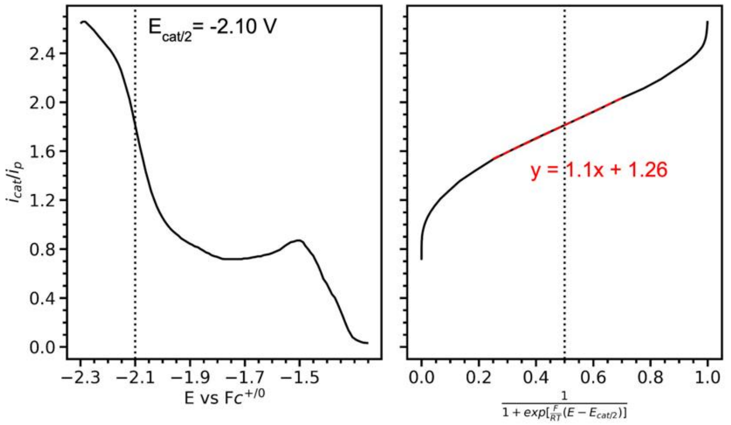

FOTW slope	1.1
$E_{cat/2}$	-2.10 V
$E^0_{CO/CO2}$	n.r.
i_p	0.58 mA/cm^2
Scan Rate	0.1 V/s
Reported TOF_{max}	n.r.
Calc. TOF_{max}	1.87 /s

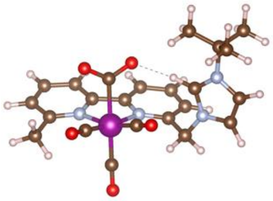

Active Catalyst	[Mn]	[CO_2]	Solvent	Proton Donor	Electrolyte	Ref.	FE_{CO}
[Mn(CO)$_3$(Im4-bpy)]	1 mM	Sat.	MeCN	5.05 M H_2O	0.1 M Bu_4NPF_6	Fc/Fc$^+$	72 %

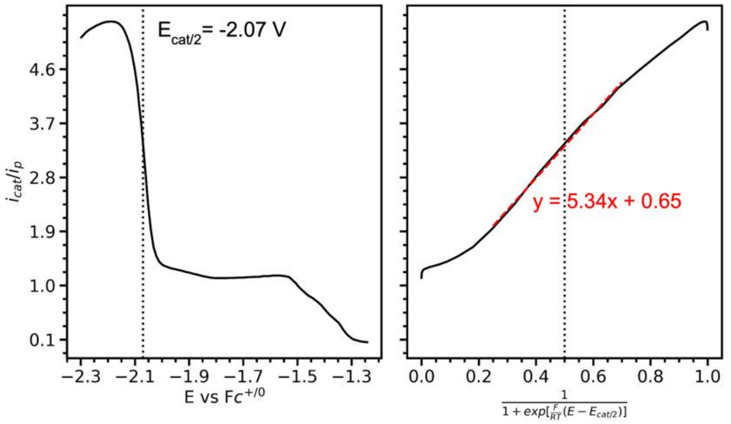

FOTW slope	5.34
$E_{cat/2}$	-2.07 V
$E^0_{CO/CO2}$	n.r.
i_p	0.62 mA/cm^2
Scan Rate	0.1 V/s
Reported TOF_{max}	n.r.
Calc. TOF_{max}	44.3 /s

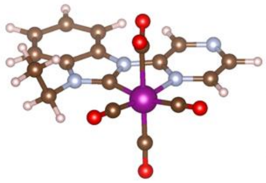

Active Catalyst	[Mn]	[CO_2]	Solvent	Proton Donor	Electrolyte	Ref.	FE_{CO}
$[Mn(CO)_3(Et\text{-}BIm\text{-}npy)]^-$	1 mM	Sat.	MeCN	5% H_2O	0.1 M Bu_4NClO_4	Fc/Fc^+	72 %

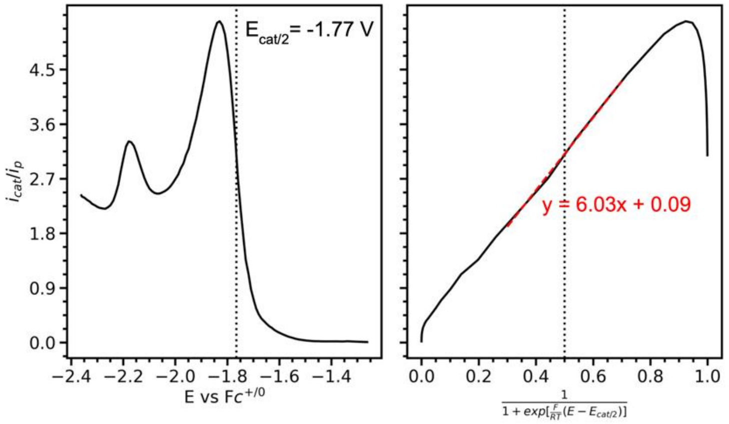

FOTW slope	6.03
$E_{cat/2}$	-1.77 V
$E^0_{CO/CO2}$	n.r.
i_p	0.55 mA/cm^2
Scan Rate	0.1 V/s
Reported TOF_{max}	42 /s
Calc. TOF_{max}	56.4 /s

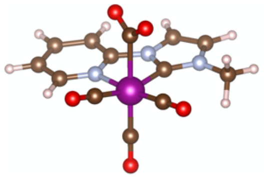

Active Catalyst	[Mn]	[CO_2]	Solvent	Proton Donor	Electrolyte	Ref.	FE_{CO}
$[Mn(CO)_3(Me\text{-}Im\text{-}py)]^-$	1 mM	Sat.	MeCN	5% H_2O	0.1 M Bu_4NClO_4	Fc/Fc^+	35 %

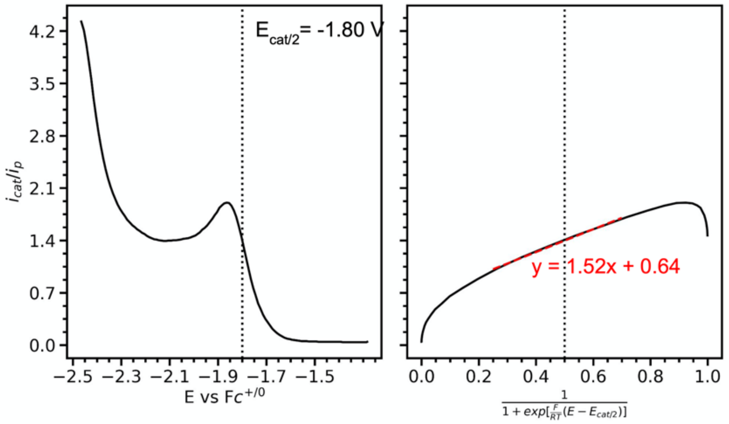

FOTW slope	1.52
$E_{cat/2}$	-1.80 V
$E^0_{CO/CO2}$	n.r.
i_p	0.23 mA/cm^2
Scan Rate	0.01 V/s
Reported TOF_{max}	0.07 /s
Calc. TOF_{max}	0.044 /s

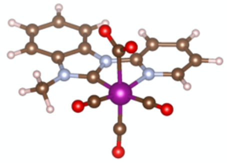

Active Catalyst	[Mn]	[CO_2]	Solvent	Proton Donor	Electrolyte	Ref.	FE_{CO}
$[Mn(CO)_3(Me\text{-}BIm\text{-}py)]^-$	1 mM	Sat.	MeCN	5% H_2O	0.1 M Bu_4NClO_4	Fc/Fc^+	n.r.

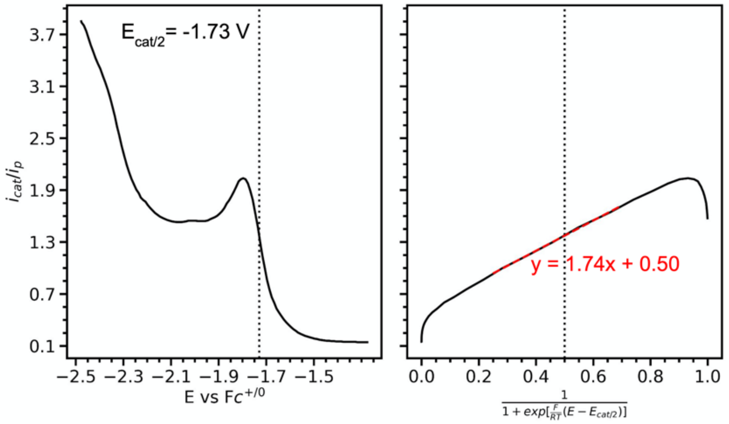

FOTW slope	1.74
$E_{cat/2}$	-1.73 V
$E^0_{CO/CO2}$	n.r.
i_p	0.18 mA/cm^2
Scan Rate	0.01 V/s
Reported TOF_{max}	0.08 /s
Calc. TOF_{max}	0.059 /s

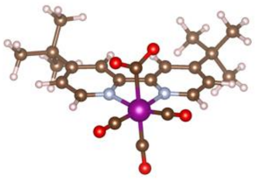

Active Catalyst	[Mn]	[CO_2]	Solvent	Proton Donor	Electrolyte	Ref.	FE_{CO}
$[Mn(CO)_3(tBu\text{-}bpy)]^-$	1 mM	Sat.	MeCN	2.65 M H_2O	0.1 M Bu_4NPF_6	Fc/Fc^+	n.r.

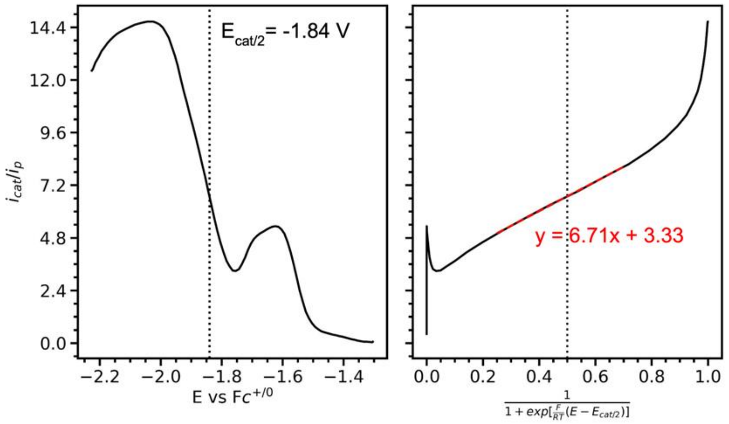

FOTW slope	6.71
$E_{cat/2}$	-1.84 V
$E^0_{CO/CO2}$	n.r.
i_p	0.40 mA/cm^2
Scan Rate	0.1 V/s
Reported TOF_{max}	120 /s
Calc. TOF_{max}	8.74 /s

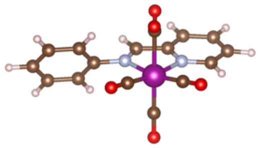

Active Catalyst	[Mn]	[CO_2]	Solvent	Proton Donor	Electrolyte	Ref.	FE_{CO}
$[Mn(CO)_3(IMP)]^-$	1 mM	Sat.	MeCN	4.7% H_2O	0.2 M Bu_4NPF_6	Fc/Fc^+	n.r.

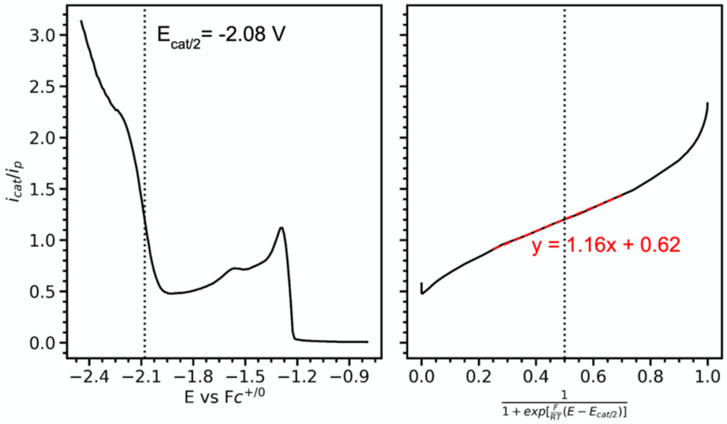

FOTW slope	1.16
$E_{cat/2}$	-2.08 V
$E^0_{CO/CO2}$	n.r.
i_p	0.045 mA
Scan Rate	0.1 V/s
Reported TOF_{max}	n.r.
Calc. TOF_{max}	0.26 /s

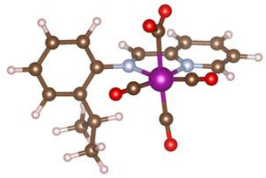

Active Catalyst	[Mn]	[CO_2]	Solvent	Proton Donor	Electrolyte	Ref.	FE_{CO}
$[Mn(CO)_3(IPIMP)]^-$	1 mM	Sat.	MeCN	4.7% H_2O	0.2 M Bu_4NPF_6	Fc/Fc^+	n.r.

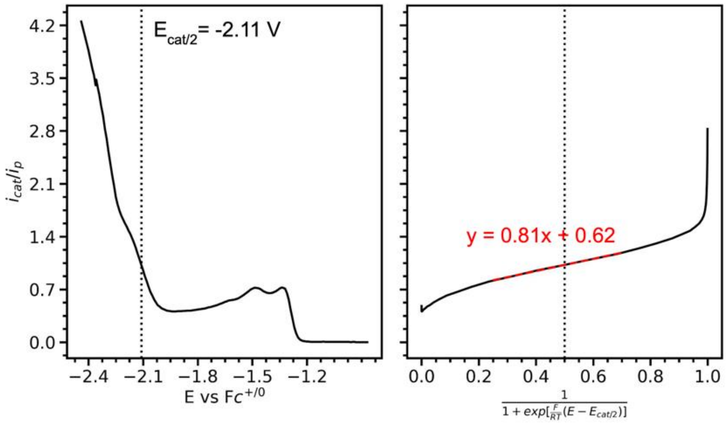

FOTW slope	0.81
$E_{cat/2}$	-2.11 V
$E^0_{CO/CO2}$	
i_p	0.052 mA
Scan Rate	0.1 V/s
Reported TOF_{max}	n.r.
Calc. TOF_{max}	0.13 /s

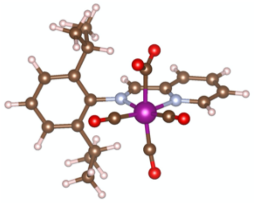

Active Catalyst	[Mn]	[CO_2]	Solvent	Proton Donor	Electrolyte	Ref.	FE_{CO}
$[Mn(CO)_3(DIPIMP)]^-$	1 mM	Sat.	MeCN	4.7% H_2O	0.2 M Bu_4NPF_6	Fc/Fc$^+$	n.r.

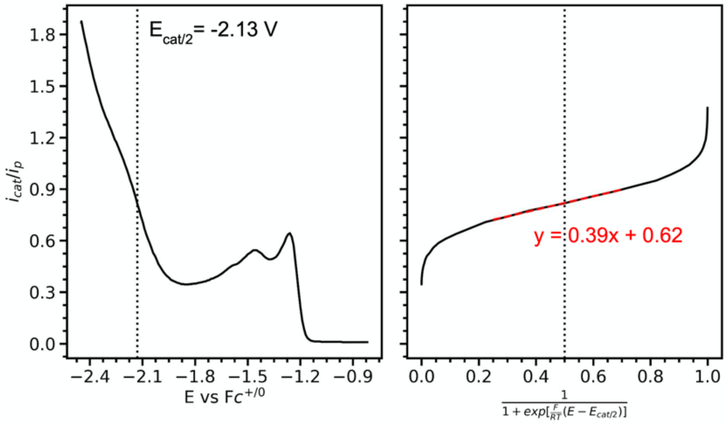

FOTW slope	0.39
$E_{cat/2}$	-2.13 V
$E^0_{CO/CO2}$	
i_p	0.050 mA
Scan Rate	0.1 V/s
Reported TOF_{max}	n.r.
Calc. TOF_{max}	0.24 /s

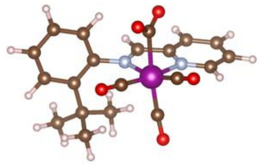

Active Catalyst	[Mn]	[CO_2]	Solvent	Proton Donor	Electrolyte	Ref.	FE_{CO}
$[Mn(CO)_3(TBIMP)]^-$	1 mM	Sat.	MeCN	4.7% H_2O	0.2 M Bu_4NPF_6	Fc/Fc$^+$	n.r.

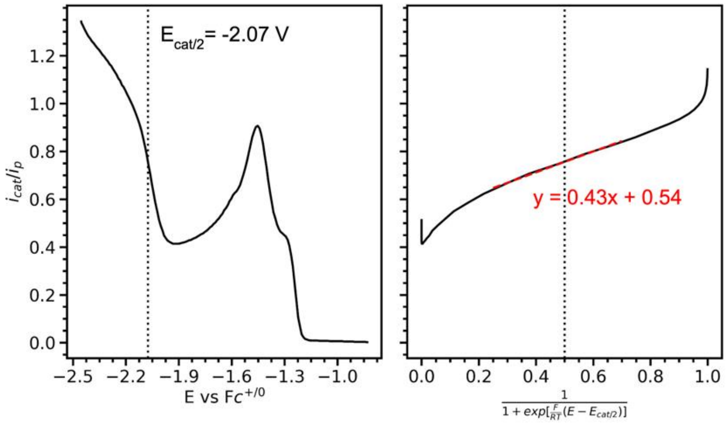

FOTW slope	0.43
$E_{cat/2}$	-2.07 V
$E^0_{CO/CO2}$	
i_p	0.052 mA
Scan Rate	0.1 V/s
Reported TOF_{max}	n.r.
Calc. TOF_{max}	0.036 /s

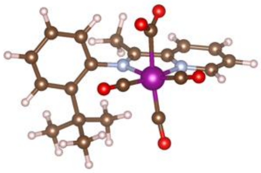

Active Catalyst	[Mn]	[CO$_2$]	Solvent	Proton Donor	Electrolyte	Ref.	FE$_{CO}$
[Mn(CO)$_3$(TBIEP)]$^-$	1 mM	Sat.	MeCN	4.7% H_2O	0.2 M Bu_4NPF_6	Fc/Fc$^+$	n.r.

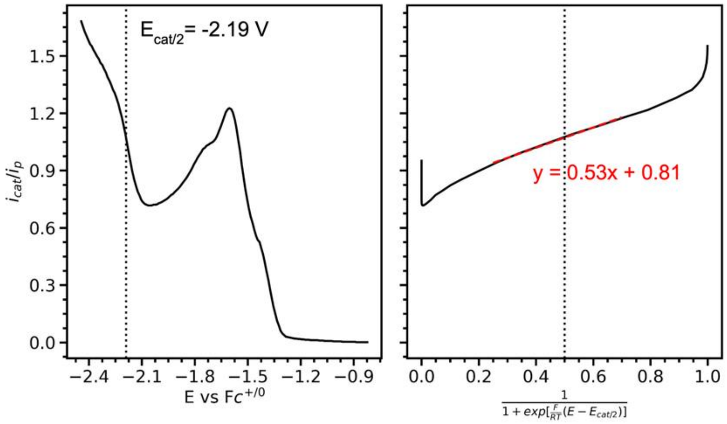

FOTW slope	0.53
E$_{cat/2}$	-2.19 V
E$^0_{CO/CO2}$	n.r.
i$_p$	0.033 mA
Scan Rate	0.1 V/s
Reported TOF$_{max}$	n.r.
Calc. TOF$_{max}$	0.054 /s

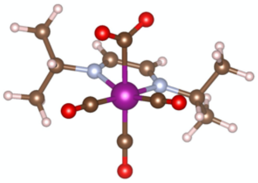

Active Catalyst	[Mn]	[CO$_2$]	Solvent	Proton Donor	Electrolyte	Ref.	FE$_{CO}$
[Mn(CO)$_3$(iPr-DAB)]$^-$	1 mM	Sat.	MeCN	5% H_2O	0.1 M Bu_4NPF_6	Fc/Fc$^+$	n.r.

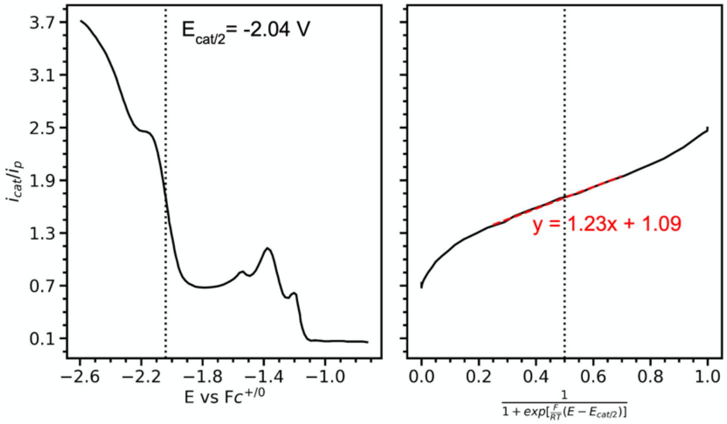

FOTW slope	1.23
E$_{cat/2}$	-2.04 V
E$^0_{CO/CO2}$	n.r.
i$_p$	74 µA
Scan Rate	0.1 V/s
Reported TOF$_{max}$	n.r.
Calc. TOF$_{max}$	0.29 /s

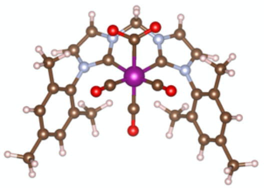

**Active Catalyst**	**[Mn]**	**[CO_2]**	**Solvent**	**Proton Donor**	**Electrolyte**	**Ref.**	**FE_{CO}**
$[Mn(CO)_3(Mes\text{-}NHC)]^-$	0.5 mM	Sat.	MeCN	0.55 M H_2O	0.1 M Bu_4NClO_4	Fc/Fc^+	95%

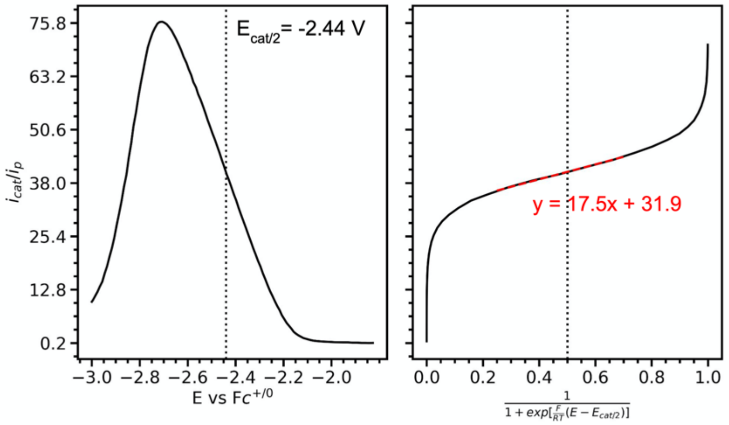

FOTW slope	17.5
$E_{cat/2}$	-2.44 V
$E^0_{CO/CO2}$	n.r.
i_p	0.30 mA/cm^2
Scan Rate	0.1 V/s
Reported TOF_{max}	3180 /s
Calc. TOF_{max}	475 /s

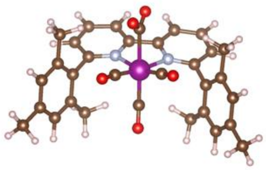

**Active Catalyst**	**[Mn]**	**[CO_2]**	**Solvent**	**Proton Donor**	**Electrolyte**	**Ref.**	**FE_{CO}**
$[Mn(CO)_3(Mes\text{-}bpy)]^-$	1 mM	Sat.	MeCN	7.2 M H_2O	0.1 M Bu_4NPF_6	Fc/Fc^+	98%

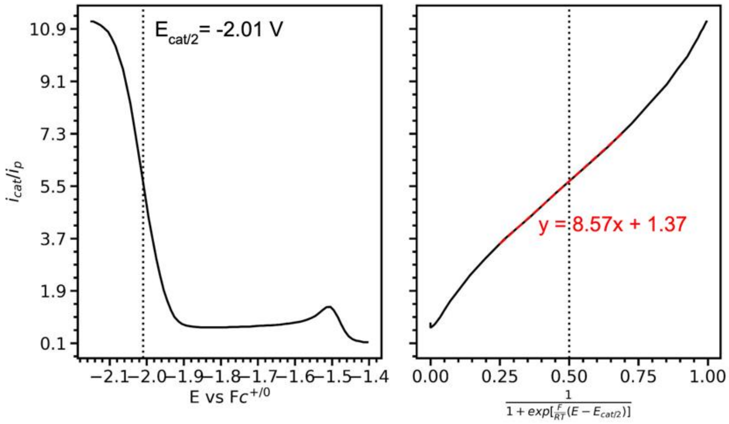

FOTW slope	8.57
$E_{cat/2}$	-2.01 V
$E^0_{CO/CO2}$	n.r.
i_p	0.58 mA/cm^2
Scan Rate	0.1 V/s
Reported TOF_{max}	n.r.
Calc. TOF_{max}	114 /s

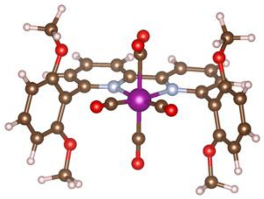

Active Catalyst	[Mn]	[CO_2]	Solvent	Proton Donor	Electrolyte	Ref.	FE_{CO}
$[Mn(CO)_3(MeOPh\text{-}bpy)]^-$	1 mM	Sat.	MeCN	6.33 M H_2O	0.1 M Bu_4NPF_6	Fc/Fc^+	61%

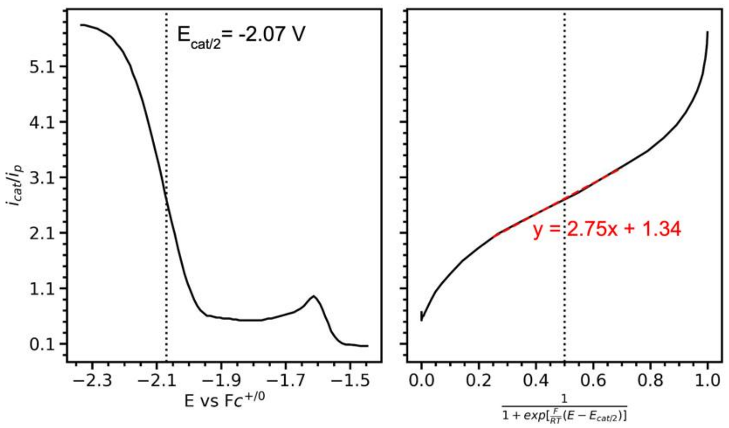

FOTW slope	2.75
$E_{cat/2}$	-2.07 V
$E^0_{CO/CO2}$	n.r.
i_p	n.r.
Scan Rate	0.5 V/s
Reported TOF_{max}	258 /s
Calc. TOF_{max}	58.7 /s

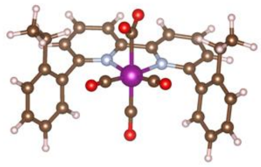

Active Catalyst	[Mn]	[CO_2]	Solvent	Proton Donor	Electrolyte	Ref.	FE_{CO}
$[Mn(CO)_3(6\text{-}EtPh\text{-}bpy)]^-$	1 mM	Sat.	MeCN	2.0 M TFE	0.1 M Bu_4NPF_6	Fc/Fc^+	94 %

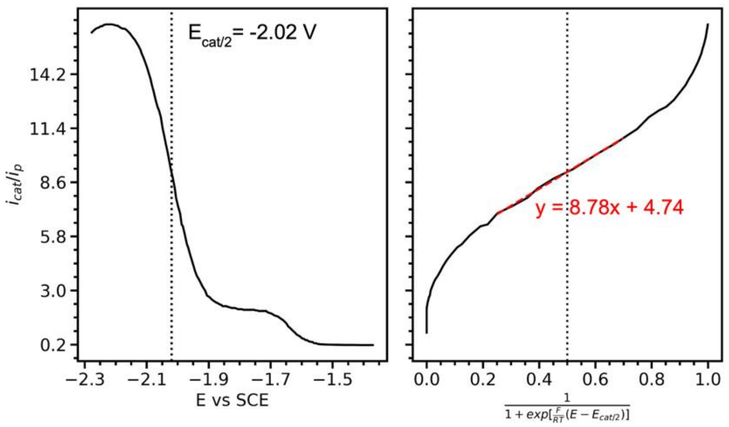

FOTW slope	8.78
$E_{cat/2}$	-2.02 V
$E^0_{CO/CO2}$	n.r.
i_p	0.7 mA/cm^2
Scan Rate	0.1 V/s
Reported TOF_{max}	700 /s
Calc. TOF_{max}	120 /s

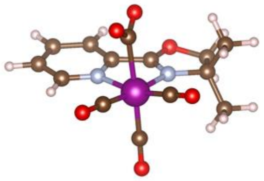

Active Catalyst	[Mn]	[CO_2]	Solvent	Proton Donor	Electrolyte	Ref.	FE_{CO}
$[Mn(CO)_3(pyrox)]^-$	1 mM	Sat.	MeCN	1.0 M TFE	0.1 M Bu_4NBF_4	NHE	99 %

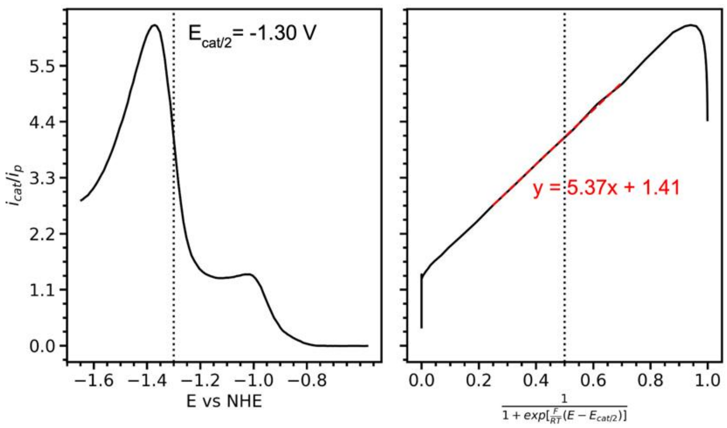

FOTW slope	5.37
$E_{cat/2}$	-1.30 V
$E^0_{CO/CO2}$	n.r.
i_p	0.75 mA/cm^2
Scan Rate	0.1 V/s
Reported TOF_{max}	40 /s
Calc. TOF_{max}	44.8 /s

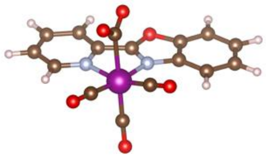

Active Catalyst	[Mn]	[CO_2]	Solvent	Proton Donor	Electrolyte	Ref.	FE_{CO}
$[Mn(CO)_3(benzox)]^-$	1 mM	Sat.	MeCN	1.0 M TFE	0.1 M Bu_4NBF_4	NHE	n.r.

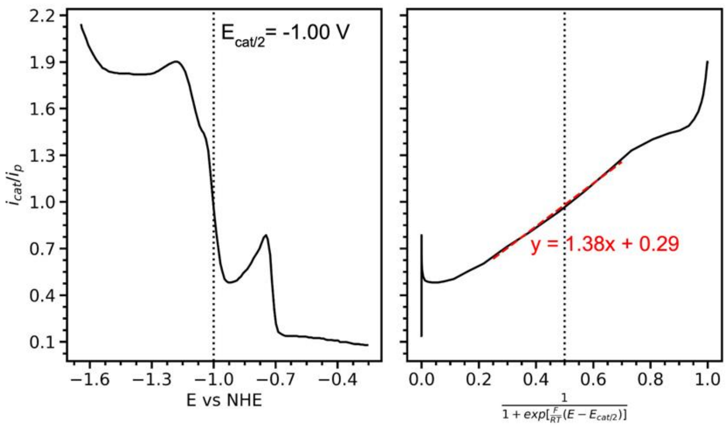

FOTW slope	1.38
$E_{cat/2}$	-1.00 V
$E^0_{CO/CO2}$	n.r.
i_p	0.84 mA/cm^2
Scan Rate	0.1 V/s
Reported TOF_{max}	n.r.
Calc. TOF_{max}	2.95 /s

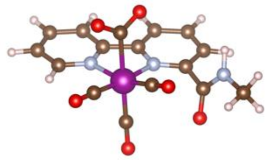

Active Catalyst	[Mn]	[CO_2]	Solvent	Proton Donor	Electrolyte	Ref.	FE_{CO}
[Mn(CO)$_3$(COHNMe-bpy)]$^-$	1 mM	Sat.	MeCN	5.5 M H_2O	0.1 M Bu_4NClO_4	Fc/Fc^+	90%

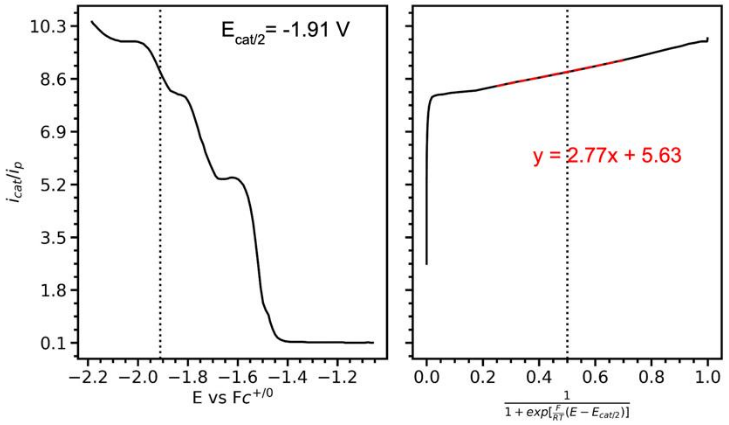

FOTW slope	1.83
$E_{cat/2}$	-1.91 V
$E^0_{CO/CO2}$	n.r.
i_p	0.35 mA/cm^2
Scan Rate	0.1 V/s
Reported TOF_{max}	79 /s
Calc. TOF_{max}	0.65 /s

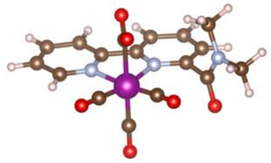

Active Catalyst	[Mn]	[CO_2]	Solvent	Proton Donor	Electrolyte	Ref.	FE_{CO}
[Mn(CO)$_3$(CONMe$_2$-bpy)]$^-$	1 mM	Sat.	MeCN	5.5 M H_2O	0.1 M Bu_4NClO_4	Fc/Fc^+	87%

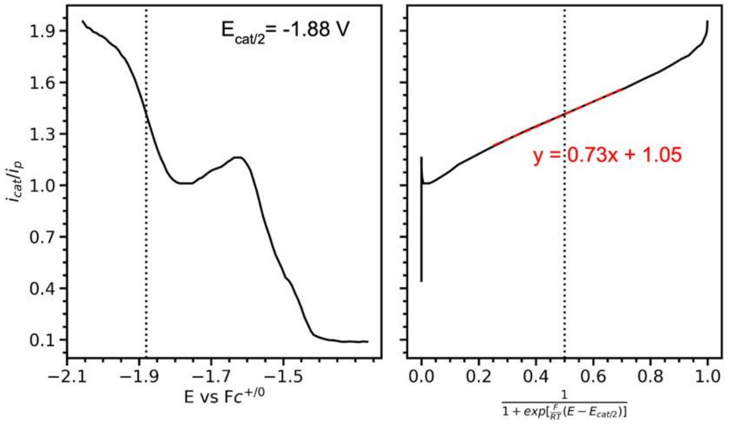

FOTW slope	0.73
$E_{cat/2}$	-1.88 V
$E^0_{CO/CO2}$	n.r.
i_p	0.59 mA/cm^2
Scan Rate	0.1 V/s
Reported TOF_{max}	n.r.
Calc. TOF_{max}	0.83 /s

References

1 E. S. Wiedner and R. M. Bullock, Electrochemical Detection of Transient Cobalt Hydride Intermediates of Electrocatalytic Hydrogen Production, *J. Am. Chem. Soc.*, 2016, **138**, 8309–8318.

2 A. M. Appel and M. L. Helm, Determining the overpotential for a molecular electrocatalyst, *ACS Catal.*, 2014, **4**, 630–633.

3 S. S. Roy, K. Talukdar and J. W. Jurss, Electro- and Photochemical Reduction of CO_2 by Molecular Manganese Catalysts: Exploring the Positional Effect of Second-Sphere Hydrogen-Bond Donors, *ChemSusChem*, 2021, **14**, 662–670.

4 I. Azcarate, C. Costentin, M. Robert and J. M. Savéant, Through-Space Charge Interaction Substituent Effects in Molecular Catalysis Leading to the Design of the Most Efficient Catalyst of CO_2-to-CO Electrochemical Conversion, *J. Am. Chem. Soc.*, 2016, **138**, 16639–16644.

5 E. S. Rountree, B. D. McCarthy, T. T. Eisenhart and J. L. Dempsey, Evaluation of homogeneous electrocatalysts by cyclic voltammetry, *Inorg. Chem.*, 2014, **53**, 9983–10002.

6 V. V. Pavlishchuk and A. W. Addison, Conversion constants for redox potentials measured versus different reference electrodes in acetonitrile solutions at 25°C, *Inorganica Chim. Acta*, 2000, **298**, 97–102.

7 N. Olmstead, Z. Margolin and F. G. Bordwell, Acidities of Water and Simple Alcohols in Dimethyl Sulfoxide Solution, *J. Org Chem.*, 1980, **45**, 3295–3299.

8 A. Kütt, S. Tshepelevitsh, J. Saame, M. Lõkov, I. Kaljurand, S. Selberg and I. Leito, Strengths of Acids in Acetonitrile, *European J. Org. Chem.*, 2021, **2021**, 1407–1419.

9 Y. C. Lam, R. J. Nielsen, H. B. Gray and W. A. Goddard, A Mn Bipyrimidine Catalyst Predicted to Reduce CO_2 at Lower Overpotential, *ACS Catal.*, 2015, **5**, 2521–2528.

10 J. M. Savéant, *Elements of Molecular and Biomolecular Electrochemistry*, Wiley-Inter, New York, 2006.

11 A. Gennaro, A. A. Isse and E. Vianello, Solubility and electrochemical determination of CO_2 in some dipolar aprotic solvents, *J. Electroanal. Chem.*, 1990, **289**, 203–215.

12 C. Costentin, S. Drouet, M. Robert and J.-M. Savéant, A Local Proton Source Enhances CO_2 Electroreduction to CO by a Molecular Fe Catalyst, *Science (80).*, 2012, **338**, 90–94.

13 K. J. Lee, N. Elgrishi, B. Kandemir and J. L. Dempsey, Electrochemical and spectroscopic methods for evaluating molecular electrocatalysts, *Nat. Rev. Chem.*, DOI:10.1038/s41570-017-0039.

14 J. M. Savéant and K. B. Su, Homogeneous redox catalysis of electrochemical reaction. Part VI. Zone diagram representation of the kinetic regimes, *J. Electroanal. Chem.*, 1984, **171**, 341–349.

15 C. Costentin, S. Drouet, M. Robert and J. M. Savéant, Turnover numbers, turnover frequencies, and overpotential in molecular catalysis of electrochemical reactions. Cyclic voltammetry and preparative-scale electrolysis, *J. Am. Chem. Soc.*, 2012, **134**, 11235–11242.

16 C. Costentin and J.-M. Savéant, Multielectron, Multistep Molecular Catalysis of

Electrochemical Reactions: Benchmarking of Homogeneous Catalysts, *ChemElectroChem*, 2014, **1**, 1226–1236.

17 C. Costentin, M. Robert and J. M. Savéant, Catalysis of the electrochemical reduction of carbon dioxide, *Chem. Soc. Rev.*, 2013, **42**, 2423–2436.

18 V. C. C. Wang and B. A. Johnson, Interpreting the Electrocatalytic Voltammetry of Homogeneous Catalysts by the Foot of the Wave Analysis and Its Wider Implications, *ACS Catal.*, 2019, **9**, 7109–7123.

19 J. D. Shipp, H. Carson, S. J. P. Spall, S. C. Parker, D. Chekulaev, N. Jones, M. Y. Mel'nikov, C. C. Robertson, A. J. H. M. Meijer and J. A. Weinstein, Sterically hindered Re- and Mn-CO_2 reduction catalysts for solar energy conversion, *Dalt. Trans.*, 2020, **49**, 4230–4243.

20 K. T. Ngo, M. McKinnon, B. Mahanti, R. Narayanan, D. C. Grills, M. Z. Ertem and J. Rochford, Turning on the Protonation-First Pathway for Electrocatalytic CO_2 Reduction by Manganese Bipyridyl Tricarbonyl Complexes, *J. Am. Chem. Soc.*, 2017, **139**, 2604–2618.

21 C. Steinlechner, A. F. Roesel, E. Oberem, A. Päpcke, N. Rockstroh, F. Gloaguen, S. Lochbrunner, R. Ludwig, A. Spannenberg, H. Junge, R. Francke and M. Beller, Selective Earth-Abundant System for CO_2 Reduction: Comparing Photo- and Electrocatalytic Processes, *ACS Catal.*, 2019, **9**, 2091–2100.

22 B. Tummers, DataThief III. 2006 <https://datathief.org>

23 F. Franco, M. F. Pinto, B. Royo and J. Lloret-Fillol, A Highly Active N-Heterocyclic Carbene Manganese(I) Complex for Selective Electrocatalytic CO_2 Reduction to CO, *Angew. Chemie - Int. Ed.*, 2018, **57**, 4603–4606.
